# The origin and evolution of plant cystatins and their target cysteine proteinases indicate a complex functional relationship

**DOI:** 10.1186/1471-2148-8-198

**Published:** 2008-07-10

**Authors:** Manuel Martinez, Isabel Diaz

**Affiliations:** 1 Laboratorio de Bioquímica y Biología Molecular, Dpto. de Biotecnología-Centro de Biotecnología y Genómica de Plantas-Universidad Politécnica de Madrid, ETS Ingenieros Agrónomos, Ciudad Universitaria s/n. 28040 Madrid, Spain

## Abstract

**Background:**

Cystatins and their putative targets, the families of cysteine proteinases C1A and C13 play key roles in plants. Comparative genomic analyses are powerful tools to obtain valuable insights into the conservation and evolution of the proteinases and their proteinaceous inhibitors, and could aid to elucidate issues concerning the function of these proteins.

**Results:**

We have performed an evolutionary comparative analysis of cysteine proteinases C1A and C13 and their putative inhibitors in representative species of different taxonomic groups that appeared during the evolution of the Viridiplantae. The results indicate that whereas C1A cysteine proteinases are present in all taxonomic groups, cystatins and C13 cysteine proteinases are absent in some basal groups. Moreover, gene duplication events have been associated to the increasing structural and functional complexities acquired in land plants.

**Conclusion:**

Comparative genomic analyses have provided us valuable insights into the conservation and evolution of the cystatin inhibitory family and their putative targets, the cysteine proteinases from families C1A and C13. Functionality of both families of proteins in plants must be the result of a coevolutionary process that might have occurred during the evolution of basal and land plants leading to a complex functional relationship among them.

## Background

Proteinaceous peptidase inhibitors are proteins that have the potential to attenuate the activities of peptidases by the formation of complexes with the enzymes. In the MEROPS database (release 8.00), 56 different families of peptidase inhibitors are included [1,2]. One of them corresponds to a family of peptidase inhibitors called cystatins, which constitute a superfamily of evolutionary related proteins able to inhibit cysteine proteinases from the papain subfamily C1A. Those from plants are called phytocystatins (PhyCys) and form an independent subfamily that cluster on a distinct branch from other cystatin families on the phylogenetic tree [3]. The cystatin inhibitory mechanism involves a wedge formed by the partially flexible N-terminus containing a glycine residue and two hairpin loops carrying a highly conserved motif QXVXG and a tryptophan residue, respectively [4,5]. Most PhyCys have a molecular mass in the 12–16 kDa range and are devoid both, of disulphide bonds and of putative glycosilation sites. However, several PhyCys with a molecular mass of ≈ 23 kDa have a carboxy-terminal extension, which has been involved in the inhibition of a second family of cysteine peptidases, the legumain peptidases C13 [6]. PhyCys have a dual role. In the plant, they have been related to the regulation of activity of endogenous cysteine proteinases during seed development and germination [7-10], and of programmed cell death [11,12]. Furthermore, a defense role has been inferred to PhyCys from their ability to inhibit exogenous proteinases such as those present in the digestive tracts of insects [13-15], the enhanced resistance against pests observed in transgenic plants overexpressing PhyCys genes [15-18], and the antifungal activities described for certain PhyCys [19-23].

The main target of PhyCys, the papain-like subfamily C1A is the most thoroughly studied among plant cysteine proteinases. Papain contains three disulfide bonds and its chain is folded to form a globular protein with two interacting domains delimiting a cleft at the surface where substrates can be bound [24]. The evolutionary highly conserved catalytic mechanism of these peptidases involves the three amino acids Cys 25, His 159 and Asn 175 (according to the papain numbering). These enzymes are synthesized as inactive precursors, which comprise an N-terminal signal peptide, a 38–250 residues prosequence, and the mature protein generally 220–260 amino acids long. Activation takes place by limited intra- or inter-molecular proteolysis cleaving off an inhibitory propeptide [25]. In plants, papain-like peptidases are involved in various physiological processes, such as the post-translational processing of storage proteins into mature forms and the liberation of amino acids to be used during germination [26-29]. An important role in the intracellular catabolism for senescence and programmed cell death has been also attributed to papain-like enzymes [11,30,31]. Moreover, a role in stress tolerance and defence against pathogens has been postulated [32-34].

The second target to cystatins is the legumain-like family C13 of cysteine proteinases. Their tertiary structure has not been reported yet, but similarities in sequence and predicted secondary structure around their active site residues Histidine and Cysteine lead Chen et al. [35] to suggest that their fold is similar to that of the caspases in family C14. Legumain is synthesized as a precursor with both N- and C-terminal propeptides. Prolegumain is directed to the plant vacuole, where activation occurs at least partially by autolysis [36]. In plants, legumains or VPE (vacuolar processing enzymes) catalyze an Asn- and Asp-specific limited proteolysis. There is abundant evidence indicating that legumains perform a protein-processing function that causes a limited proteolysis of precursor proteins [37]. Legumain from plant seeds is thought to be responsible for the post-translational processing of seed proteins prior to storage [38]. During germination, legumains contribute to the activation of cysteine proteinases to degrade storage proteins [39,40]. A role in defense against pathogens executing programmed cell death due to the caspase activity observed for several legumains has also been proposed [41,42].

Although peptidase-inhibitor interactions are crucial to many important processes in plant cells, very little information is available on the origin and evolution of these protein families in plants. To date, a comprehensive analysis of cystatin families has been only done in the completed genomes of the mono and dicotyledoneous model plants *Oryza sativa *and *Arabidopsis thaliana *[43]. Likewise, the family of papain-like cysteine proteinases from Arabidopsis was formerly described by Beers et al. [44], and different families of putative cysteine proteinases in the completely sequenced genomes of *Populus trichocarpa *and Arabidopsis have been reviewed by Garcia-Lorenzo et al. [45].

Comparative genomic analyses could provide valuable insights into the conservation and evolution of the proteinases and their proteinaceous inhibitors, which could aid to elucidate issues concerning the function of these proteins. Thus, we have performed a phylogenetic analysis of these gene families in representative species of different taxonomic groups. The results indicate that whereas papain-like cysteine proteinases are present in all taxonomic groups, cystatins and legumain-like proteins are absent in some basal groups. Moreover, gene duplication events have been associated to the increasing structural and functional complexities acquired in land plants.

## Results

### A survey of cystatins and cysteine proteinases (C1A and C13) in plants

**Figure 1:**
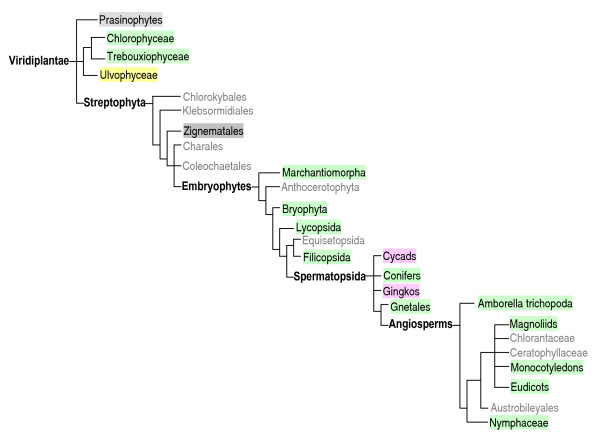
**Schematic evolutionary tree of Viridiplantae based on the Tree of Life Web Project.** Different colours indicate the presence of any modern species in the clade with at least one transcript assembly belonging to the cystatin family, the papain-like C1A, and/or the legumain-like C13 cysteine proteinase families. Green, presence of cystatin, C1A and C13 proteins. Grey, presence of C1A and C13 proteins. Yellow, presence of C1A proteins. Pink, presence of cystatin and C1A proteins. Absence of colour indicates clades without representative species in the TIGR plant transcript assemblies database.

In a first step to know the origin and evolution of cystatins and papain and legumain-like cysteine proteinases in plants, an extensive search of the TIGR plant transcript assemblies (TA) database was made. All plant species for which more than 1,000 ESTs or cDNA sequences are available are included in the database. Thus, TAs from 252 plant species from unicellular organisms to flowering plants could be explored. In addition, the Tree of Life Web Project provides information on the evolutionary phylogenetic relationships in the green plants. Based on this information, we constructed a tree with the main clades of modern plants (Fig. 1). In this tree, the presence of any species of each clade with a TA belonging to the cystatin family, or to the papain-like or legumain-like cysteine proteinase families is represented. The clades of the Embryophytes or land plants present cystatins and both types of cysteine proteinases, with the exception of Cycads and Gingkos, in which no legumain-like cysteine proteinases have been detected yet. In contrast, the algae have a great variability of detected proteinases and inhibitors. Thus, whereas cystatins have not been detected in Prasinophytes, Ulvophyceae and Zignematales, the algae groups Chlorophyceae and Trebouxiophyceae possess both proteinases and inhibitors. This approximation permitted us a survey of how these protein families could have evolved. However, the actual number of papain and legumain-like proteinases and cystatins encoded in a plant genome can only be known using the whole genome sequence.

### Number of cystatins and cysteine proteinases (C1A and C13) in completely sequenced plants

To date, four algae (two Prasinophytes, *Ostreococcus tauri *and *Ostreococcus lucimarinus*; and two Chlorophyceae, *Chlamydomonas reinhardtii *and *Volvox carteri*), one moss (*Physcomitrella patens*), one spikemoss (*Selaginella mollendorffii*), and three angiosperms (*Arabidopsis thaliana*, *Populus trichocarpa *and *Oryza sativa*) have been completely sequenced and drafts of these sequences are available on the web. These eight species and the monocot species *Hordeum vulgare*, with an extremely large number of EST and cDNA sequences available, were selected to establish the number of proteinases and inhibitors. Thus, extensive searches for C1A, C13 proteinases, and cystatins were made (Table 1). Among the algae, no cystatins were found in the two *Ostreococcus *species, whereas the Chlorophyceae species possess a unique cystatin that lacks the 3' extension related to the inhibitory properties against legumain-like proteinases. However, all these species present papain-like proteinases and the Chlorophyceae species a unique legumain proteinase. From the moss to the angiosperms, the number of cystatins gradually increased. In the moss and the spikemoss, the presence of cystatins with the 3' extension is correlated with an increase in the number of legumain-like proteins. In angiosperms, a higher number of cystatins than in the non-seed land plants is accompanied by a sharp increase in the number of papain-like cysteine proteinases.

**Table 1:** Number of C1A and C13 cysteine proteinases and their putative inhibitors.

Organism	Cystatins (with 3' extension)	Papain-like proteinases	Legumain-like proteinases
*Ostreococcus tauri*	0 (0)	9	0
*Ostreococcus lucimarinus*	0 (0)	9	0
*Chlamydomonas reinhartdii*	1 (0)	11	1
*Volvox carteri*	1 (0)	14	1
*Physcomitrella patens*	5 (3)	11	4
*Selaginella moellendorffii*	2 (1)	19	2
*Oryza sativa*	12 (1)	45	5
*Arabidopsis thaliana*	7 (2)	32	4
*Populus trichocarpa*	8 (3)	36	5
*Hordeum vulgare*	13 (1)	32	5

### Evolution of C1A cysteine-proteinases in plants

**Figure 2:**
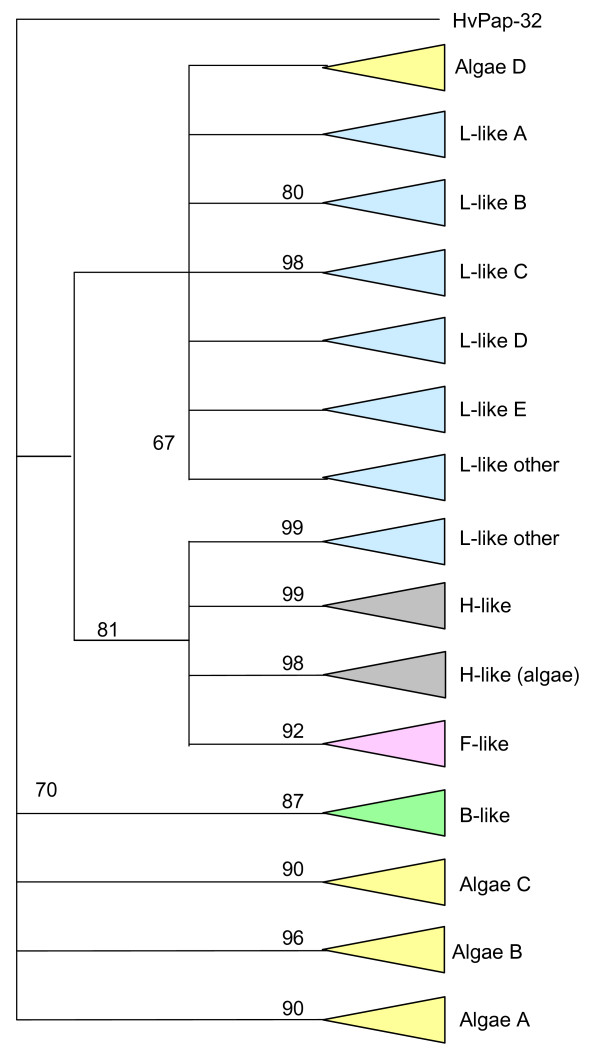
**Schematic representation of the cladogram of cysteine proteinases C1A from algae to angiosperms.** The amino acid sequences were aligned by MUSCLE and analysed with the PhyML method. Approximate likelihood-ratio test values >80% are indicated. F-like, cathepsin F-like (pink); H-like, cathepsin H-like (grey); B-like, cathepsin B-like (green); L-like, cathepsin L-like (blue). Other cysteine proteinases C1A from algae (yellow). Vc, *Volvox carteri*.

To know how papain-like cysteine proteinases could have evolved from algae to angiosperms, the C1A mature protein fragments from 25 amino acids before the putative location of the cysteine reactive residue until 18 amino acids after the asparagine reactive residue were selected. These amino acid sequences were aligned by MUSCLE (see Additional file 1, partial sequences corresponding to HvPap-23 and HvPap26 were not included), and a phylogenetic tree was constructed by the maximum likelihood PhyML method (see Additional file 2). A schematic representation of the obtained cladogram is shown in Figure 2. C1A proteinases were originally described as cathepsins in mammals, and plant cysteine proteinases have been traditionally classified based on their homology to them [44]. When the obtained C1A proteins were classified in this form, ten main groups comprising C1A proteinases homologous to the human B, F, H, and L-cathepsins were found. B-like proteins shared a similar sequence to human B-cathepsin. F-like proteinases conserved an ERFNAQ motif typical to this kind of cathepsins in their proregion. H and L-like proteins conserved the ERFNIN motif present in mammal H and L cathepsins in the proregion. Several algal papain-like proteinases (groups Algae A to D) and the barley protein HvPap-32 did not present enough similarities to mammalian cathepsins to be considered as their plant homologous proteins and were grouped separately. To deal with the extremely complex group of H and L-like cathepsins, an analysis to discover homologous motifs was made using the MEME software (data not shown). All the proteins shared a high number of identical motifs, but several ones could be used to discriminate among proteins belonging to different subgroups. From this analysis, five subgroups (A to E) of L-like proteins were defined. Two of them (B and C) correlated with well-defined branches of the phylogenetic tree and were formed by only angiosperm proteins, as well as group A which was split in several branches. Group D includes branches of L-like C1A sequences composed of algae, moss, spikemoss and angiosperm proteins. Three branches of the phylogenetic tree formed by cereal sequences could be assigned to the subgroup E. Some other branches formed by C1A proteins for all clades could not be included to any subgroup and were assigned as other L-like cathepsins (Suppl. Fig. 1). For H-like cathepsins, two subgroups could be detected, one of them included C1A proteinases from the moss to angiosperms, and the other was formed by algal proteins. In Table 2, the number of C1A proteinases belonging to each group is shown. B-like C1A are present in all analyzed species and the number did not vary during plant evolution. F-like proteinases were also maintained from algae, although they were not detected in the Chlorophyceae, and their number slightly increased in angiosperms. H-like proteinases were detected in Chlorophyceae, and were maintained from moss to angiosperms. L-like proteins showed the most striking pattern. They first appeared in algae, slightly increased in the moss, and they presented a sharp increase from the spikemoss to the angiosperms. Finally, C1A proteinases not related to mammalian proteinases (indicated as other) were mainly found in algae.

**Table 2:** Number of C1A cysteine proteinases grouped by similarity to mammalian proteinases.

Organism	B-like	F-like	H-like	L-like	Others
*Ostreococcus tauri*	3	1	0	2	3
*Ostreococcus lucimarinus*	3	1	0	2	3
*Chlamydomonas reinhartdii*	3	0	2	2	4
*Volvox carteri*	2	0	2	2	8
*Physcomitrella patens*	3	2	1	5	
*Selaginella moellendorffii*	1	3	1	14	
*Oryza sativa*	1	3	1	40	
*Arabidopsis thaliana*	3	3	2	24	
*Populus trichocarpa*	3	5	1	27	
*Hordeum vulgare*	2	3	1	25	1

### Evolution of C13 cysteine-proteinases in plants

**Figure 3:**
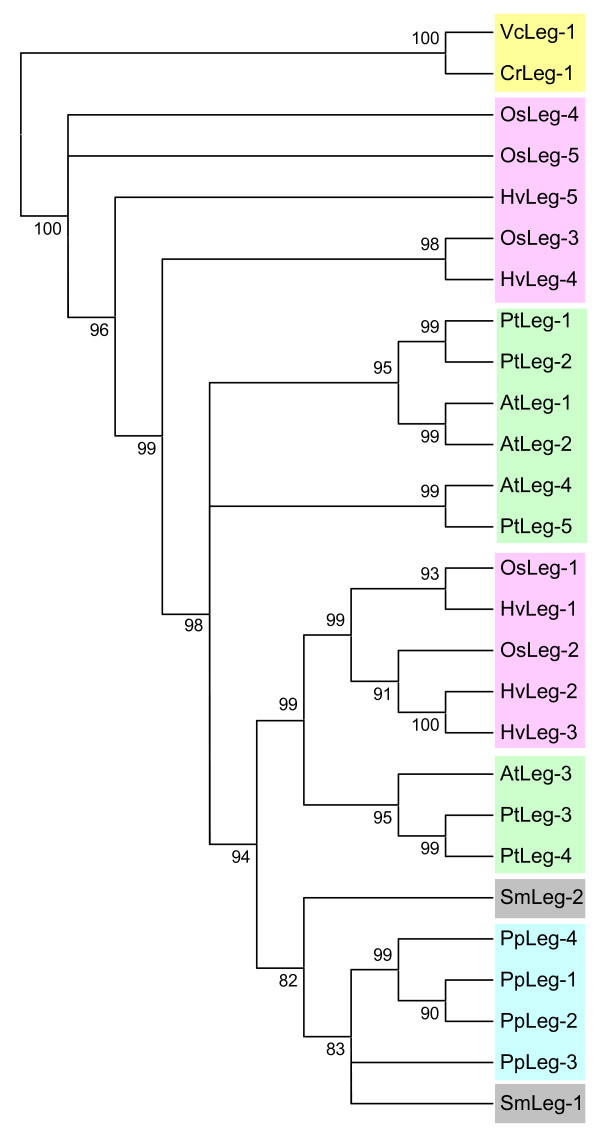
**Cladogram of the cysteine proteinases C13 from algae to angiosperms.** The amino acid sequences were aligned by MUSCLE and analysed with the PhyML method. Approximate likelihood-ratio test values >80% are indicated. Green, dicot proteins (At, *Arabidopsis thaliana*; Pt, *Populus tricocarpa*); pink, monocot proteins (Os, *Oryza sativa*; Hv, *Hordeum vulgare*); blue, moss proteins (Pp, *Physcomitrella patens*); grey, spikemoss proteins (Sm, *Selaginella moellendorffii*); yellow, algal proteins (Cr, *Chlamidomonas reinhardtii*; Vc, *Volvox carteri*).

Similarly, to know how the second target of cystatins, the legumain-like cysteine proteinases could have evolved from algae to angiosperms, the amino acid sequences of C13 proteinases were aligned by MUSCLE (see Additional file 1), and a cladogram was constructed by the maximum likelihood PhyML method (Fig. 3). The C13 proteins from Chlorophyceae were the most different and did not grouped with the other legumain sequences. Orthology relationships could be detected for angiosperm proteins located in groups supported by approximate likelihood-ratio test values (aLRT) higher than 80%, mainly among putatively orthologous monocot or dicot proteins. On the contrary, no orthology could be detected among moss and spikemoss sequences and the rest of C13 proteinases.

### Evolution of cystatins in plants

**Figure 4:**
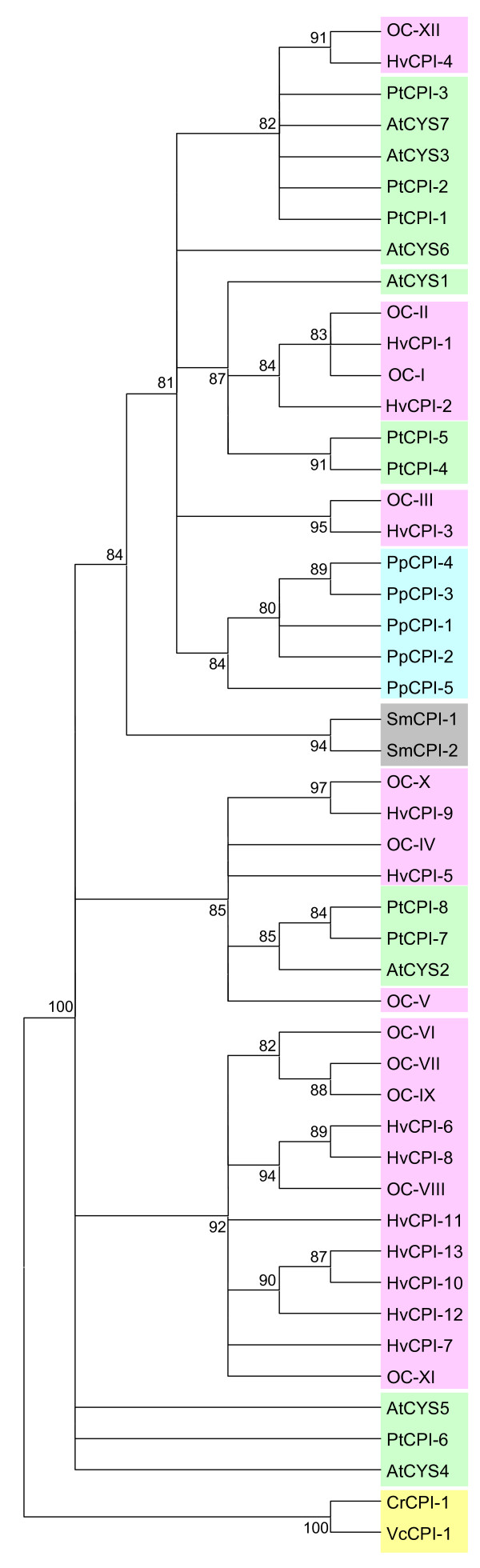
**Cladogram of the cystatins from algae to angiosperms.** The amino acid sequences were aligned by MUSCLE and analysed with the PhyML method. Approximate likelihood-ratio test values >80% are indicated. Green, dicot proteins (At, *Arabidopsis thaliana*; Pt, *Populus tricocarpa*); pink, monocot proteins (Os, *Oryza sativa*; Hv, *Hordeum vulgare*); blue, moss proteins (Pp, *Physcomitrella patens*); grey, spikemoss proteins (Sm, *Selaginella moellendorffii*); yellow, algal proteins (Cr, *Chlamidomonas reinhardtii*; Vc, *Volvox carteri*).

**Figure 5:**
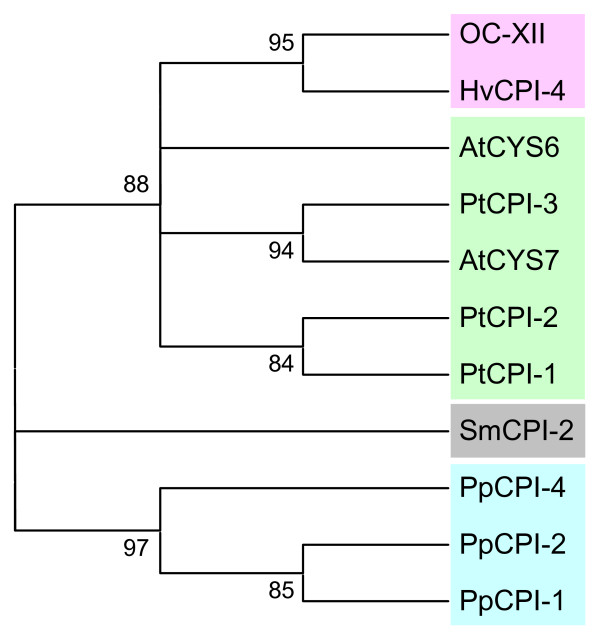
**Cladogram of the carboxy terminal domain of long cystatins from the moss to angiosperms.** The amino acid sequences were aligned by MUSCLE and analysed with the PhyML method. Approximate likelihood-ratio test values >80% are indicated. Green, dicot proteins (At, *Arabidopsis thaliana*; Pt, *Populus tricocarpa*); pink, monocot proteins (Os, *Oryza sativa*; Hv, *Hordeum vulgare*); blue, moss proteins (Pp, *Physcomitrella patens*); grey, spikemoss proteins (Sm, *Selaginella moellendorffii*).

**Figure 6:**
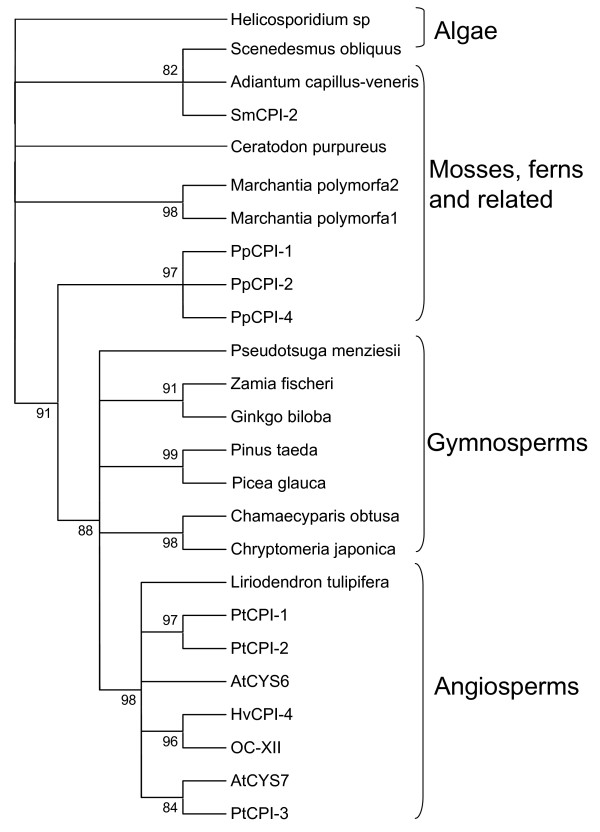
**Cladogram of the carboxy terminal domain of long cystatins deduced from transcript assemblies of different Viridiplantae clades.** The amino acid sequences were aligned by MUSCLE and analysed with the PhyML method. Approximate likelihood-ratio test values >80% are indicated. At, *Arabidopsis thaliana*; Pt, *Populus tricocarpa*; Os, *Oryza sativa*; Hv, *Hordeum vulgare*; Pp, *Physcomitrella patens*; Sm, *Selaginella moellendorffii*.

Once known the evolution of C1A and C13 cysteine proteinases along the different plant clades, we seek to know how their inhibitory proteins, the cystatins, could have evolved. Likewise, the cystatin amino acid sequences and the sequences of the carboxy terminal extensions were aligned by MUSCLE (see Additional file 1), and phylogenies were constructed by the maximum likelihood PhyML method. When the main cystatin body was compared, omitting the signal peptides and the C-terminal extensions (Fig. 4), the cystatins from Chlorophyceae were the most different to the other cystatin sequences. The rest of sequences, with the exception of PtCPI-6, AtCYS5 and AtCYS4, could be distributed in three groups, one of them containing the five cystatins from the moss, the two cystatins from the spikemoss and several cystatins from all the angiosperm species. A second group was formed by mono or dicotyledoneous sequences, and only barley and rice sequences were located in the third group. When the C-terminal extensions were compared, the cystatins from the moss and the spikemoss were grouped separately from the angiosperm sequences (Fig. 5). The low number of 3' extended cystatins in completely sequenced genomes led us to compare the sequences obtained for other plant species from the TIGR Plant Transcript Assemblies (Fig. 6). The PhyML obtained tree resembles the evolutionary relationships among plant species, from algae to angiosperms, which denotes the ancestral characteristic of these C-terminal extensions. Interestingly, when the cDNA sequences of the cystatins with a C-terminal extension of the two algae species (one Chlorophyceae and one Trebuxiophyceae) were revised, the two main domains of the protein, the cystatin and the legumain inhibitory domain were separated by a stop codon, which was not present in the rest of carboxy extended proteins from plants.

## Discussion

Nowadays, comparative genomic analysis has become as a powerful tool to discover evolutionary relationships between protein families. In this work, we analysed the evolutionary relationships among cystatins and their putative target enzymes, the cysteine proteinases C1A and C13 in different fully sequenced clades that appeared during plant evolution. To date, a comprehensive analysis of cystatin families had been only done in the completed genomes of *A. thaliana *and *O. sativa *[43]. For C1A cysteine proteinases, only the protein families in Arabidopsis and *P. trichocarpa *had been previously described [44,45]. At present, the complete protein family of C13 proteinases had not been analysed in any plant species.

The present phylogenetic study of phytocystatins indicates that this subfamily might have evolved from a common ancestor. This ancestral protein could have been lost in some algal lineages as in the Prasinophytes, and maintained in some other as the Chlorophyceae. However, the algal cystatins differ from the rest of the PhyCys in the presence of two cysteine residues, and the absence of the consensus motif [LVI]- [AGT]- [RKE]- [FY]- [AS]- [VI]-x- [EDQV]- [HYFQ]-N specific to PhyCys [3]. Later on, this protein family would have expanded in the different taxonomic groups of vascular plants by recurrent duplication events in the course of evolution. Whether the ancestral cystatin was able to inhibit both C1A and C13 cysteine proteinases remains unknown. The *C. reinhardtii *and *V. carteri *cystatins did not posses the carboxy terminal extension present in the phytocystatins that inhibit legumain-like cysteine proteinases [6]. However, some animal cystatins are able to inhibit this kind of proteinases using the main core of the cystatin domain [46]. Since both algae possess one legumain-like protein, this second possibility cannot be discarded. Interestingly, two cystatins expressed by the algae *Scenedesmus obliquus *and *Heliosporidium sp ex Simulium jonesii*, belonging to the related algae families Chlorophyceae and Trebouxiophyceae did have the carboxy terminal extension with the legumain inhibitory motif. This carboxy terminal extensions are recognized by the Pfam motif database [47] as a cystatin domain, which suggest that C-extensions of plant cystatins are degenerated parts of a dimeric cystatin that probably originated by gene duplication followed by divergent evolution. Curiously, a stop codon is located between the two domains of these algal proteins. Since they are correctly expressed, the whole protein could be translated by readthrough the stop codon. In eukaryotes, readthrough is mainly due to decoding of the internal stop as normal amino acid or a noncanonical one, as seen for *C. reinhardtii *[48], suppression by a natural non-sense suppressor tRNA [49,50], or a stable hairpin loop structure downstream of the stop codon [51].

With the exception of *C. reinhardtii *and *V. carteri*, the presence of cystatins with the C-terminal extension and legumain-like proteinases is well correlated. The Prasinophytes *O. tauri *and *O. lucimarinus *are devoid of both whereas vascular plants posses several members of these two protein families. Legumain-like cysteine proteinases are a small family in plants since no more than five members of this subfamily are present in any species. Likewise, the number of cystatins with the C-terminal extension is small, ranging from one to three. In higher plants, legumains have been involved in important physiological processes. They are implicated in the activation of papain-like cysteine proteinases to degrade storage proteins in the seed [39,40,52], and in defense against pathogens executing programmed cell death [41,42]. However, the restricted activity of these proteinases, which have not been related to an elevated number of physiological processes, could account for the small number of members of this protein family.

The case of the papain-like cysteine proteinases was significantly different. These proteins have been involved in many physiological processes in vascular plants, including protein processing in the seed, senescence and programmed cell death, pollen development, and defence against pathogens [11,27,28,31,33,53]. In algae, the large number of C1A proteinases, ranging from 9 to 14, suggests an important role of these proteins in their life cycle. However, there is only one report concerning the physiological roles of C1A cysteine proteinases. A protein, VcPap-11, is induced by the sex-inducing pheromone of the multicellular green algae *V. carteri *[54]. Interestingly, whereas the C1A proteinases from vascular plants are all related to mammalian cathepsins from the groups B, F, H and L, a large number of algal C1A members could not be assigned to these groups, including VcPap-11. This suggests specialized functions in algae for these proteins not present in vascular plants, such as the response to sex-inducing pheromones. In angiosperms, the number of putative C1A cysteine proteinases dramatically increased (mainly L-like) which could also mean a specialization of these proteinases to the new functions required in these plants. In fact, Arabidopsis AtPap-14 (XCP1) and AtPap-15 (XCP2), which belong to the angiosperm subgroup B of the L-like cathepsins, are expressed in the tracheary elements of the xylem [55]. Similarly, proteins of the L-like subgroup D formed by C1A sequences from algae to angiosperms could have evolved to achieve specific functions of higher plants. OsPap-24 and OsPap-18 (Oryzains a and b) have been involved in seed storage mobilization, and OsPap-19 (OsCPI1) in pollen development [53,56]. Unfortunately, the function of the majority of these proteinases remains enigmatic, and testing for subfunctionalization and neofunctionalization events following gene duplication exceeds the scope of this project.

## Conclusion

Comparative genomic analyses have provided us valuable insights into the conservation and evolution of the cystatin inhibitory family and their putative targets, the cysteine proteinases from families C1A and C13. A phylogenetic analysis of these gene families in representative species of different plant taxonomic groups has permitted us to state that whereas papain-like cysteine proteinases are present in all groups, cystatins and legumain-like proteins are absent in some basal groups. Moreover, in all these families, gene duplication events are associated with the increasing structural and functional complexities acquired in land plants. Thus, the complex functional relationship between both families of proteins must be the result of a coevolutionary process that might have occurred during the evolution of basal and land plants.

## Methods

### Genome databases searches

Blast searches for C1A and C13 cysteine proteinases and for cystatins were performed in publicly available genome databases. Sequences for *Oryza sativa *ssp. japonica (rice annotation release 5) were obtained at The Institute for Genomic Research (TIGR) [57]. Sequences for *Arabidopsis thaliana *were identified by searching The Arabidopsis Information Resource (TAIR) database (TAIR7 genome release) [58]. Searches for algae, moss, spikemoss and poplar sequences were carried out at the DOE Joint Genome Institute (JGI) [59], using the current releases: *Chlamydomonas reinhardtii *v3.0 [60]; *Volvox carteri *f. nagariensis v1.0 [61]; *Ostreococcus lucimarinus *v2.0 [62]; *Ostreococcus tauri *v2.0 [63]; *Physcomitrella patens *ssp. patens v1.1 [64]; *Sellaginella moellendorffii *v1.0 [65]; *Populus trichocarpa *v1.1 [66]. Information about gene models for all these proteins is compiled in Tables I, III and IV from Additional file 3.

### Screening of barley EST and cDNA libraries

C1A, C13 cysteine proteinases and cystatin sequences were used to search for ESTs or cDNA sequences in publicly available libraries of barley using the tblastn program at the DNA Data Bank of Japan [67] and the Okayama University Barley Database [68]. Selected ESTs were obtained and sequenced in both strands using vector-specific primers using an automated DNA sequencer (ABI PRISM TM 3100; Perkin Elmer-Applied Biosystems). Accession numbers are in Tables I, III and IV from additional file 3.

### Searches for expressed genes in plants

C1A, C13 cysteine proteinases and cystatin sequences were used to search the TIGR plant transcript assemblies (TA) database [69]. The sequences that are used to build the plant TAs are expressed transcripts collected from dbEST (ESTs) and the NCBI GenBank nucleotide database. Sequences are initially clustered based on an all-against-all comparisons using Megablast. The initial clusters are assembled to generate consensus sequences using CAP3. Assembly criteria include a 50 bp minimum match, 95% minimum identity in the overlap region and 20 bp maximum unmatched overhangs. Any EST/cDNA sequences that are not assembled into TAs are included as singletons. TAs from 252 plant species from unicellular organisms to flowering plants are included in this database. Transcript assembly accessions for cystatin 3' extended genes are shown in Table II from Additional file 3.

### Phylogeny of plants

The phylogenetic tree used to the survey of the evolution of C1A, C13 cysteine proteinases and cystatins was based on the Viridiplantae tree of the Tree of Life Web Project (ToL) [70], which is a collaborative effort of biologists from around the world to provide information about the diversity of organisms on Earth, their evolutionary history (phylogeny), and characteristics.

### Protein alignments and Phylogenetic trees

Alignments of the amino acid sequences were performed using the default parameters of MUSCLE version 3.6 [71]. For cystatins and cysteine proteinases C13 controversial models were not detected and the whole amino acid sequences were aligned. For cysteine proteinases C1A, presumed wrong models were detected and protein alignments were restricted to the part of the protein that was not controversial in any model and conserves the characteristic features of papain-like cysteine proteinases (from 25 amino acids before the putative location of the cysteine reactive residue until 18 amino acids after the asparagine reactive residue). Alignments ambiguities and gaps were excluded from phylogenetic analysis using GBLOCKS version 0.91b [72]. Phylogenetic and molecular evolutionary analyses were conducted using the programs PhyML and MEGA version 4.0 [73-75]. The displayed C1A, C13 cysteine proteinases and cystatin trees were derived using a maximum likelihood PhyML method using the WAG substitution model and a BIONJ starting tree. The approximate likelihood-ratio test (aLRT) based on a Shimodaira-Hasegawa-like procedure was used as statistical test for non-parametric branch support [76]. Displayed cladograms are condensed trees with cut-off aLRT values of 80%. All families were also analysed with the Maximum parsimony, Unweighted Pair Group Method with Arithmetic Mean (UPGMA), and the Neighbour-Joining algorithms, and with different gap penalties. No significant differences in the tree topologies were detected.

### Identification of conserved motifs

The deduced protein sequences of the putative H and L-like cathepsins were analysed by means of the MEME program [77,78]. Default parameters were used, except that the minimal and maximal motif widths were set to 6 and 12 amino acids, respectively, and the maximum number of motifs to find was defined as 50.

## Authors' contributions

MM designed the study, carried out the sequence analysis and interpretation of the results and drafted the manuscript. ID participated in the design of the study and the interpretation of the results and critically revised the manuscript.

## Supplementary Material

Additional File 1 Alignments performed using the MUSCLE program of the amino acid sequences corresponding to the proteins used in this study. Click here for file

Additional File 2 Complete cladogram of cysteine proteinases C1A from algae to angiosperms from which Figure 1 is derived. The amino acid sequences were aligned by MUSCLE and analysed with the PhyML method. Approximate likelihood-ratio test values >80% are indicated. Click here for file

Additional File 3 Information about gene models and accession numbers corresponding to the proteins used in this study. Click here for file
