# Supplementary material for: The origin and evolution of plant cystatins and their target cysteine proteinases indicate a complex functional relationship
Source: BMC Evol Biol. 2008 Jul 10;8:198. doi: 10.1186/1471-2148-8-198 (PMC2474614; doi:10.1186/1471-2148-8-198)
Supplement: Additional File 1 — Alignments performed using the MUSCLE program of the amino acid sequences corresponding to the proteins used in this study. [file 1471-2148-8-198-S1.doc]

**Additional file 1.**

**Figure 1.** Comparison of the amino acid sequences of the papain-like cysteine proteinases from algae to angiosperms. The alignment was generated using the MUSCLE program. At, *Arabidopsis thaliana*; Pt, *Populus tricocarpa*; Os, *Oryza sativa*; Hv, *Hordeum vulgare*; Pp, *Physcomitrella patens*; Sm, *Selaginella moellendorffii*; Cr, *Chlamidomonas reinhardtii*; Vc, *Volvox carteri*.

VcPap-14 ----PLPPMWD----SRM------KELTGDVRIISSVKN----Q-A-S--C---------

VcPap-7 ----DALSYPE----WDS-------RFSGPFNFVSPVKD----Q-G-G--C---------

CrPap-7 ----TYPV-------WDS-----RQANSTSYSFITPVKD----Q-G-S--C---------

VcPap-8 -----LYPTWD----SRS----AN-GTNNN--LISPVKD----Q-G-S--C---------

VcPap-13 ----SFPI-------WDS-----RNVTGTSYNLISPVKD----Q-G-S--C---------

VcPap-11 -----LYPVWD----SRS---------VNGSNYISPVKD----Q-G-G--C---------

VcPap-9 ----AYP-AWD----SRV---------NGSYNYVSPVKD----Q-G--------------

VcPap-10 ----TYPV-------WDS---RTANDTSYN--YISSVKD----Q-G-G--C---------

OlPap-8 ----TLPKDFDVREKWPK---------CAA--LVSEALD----Q-G-E--C---------

OtPap-9 ----RLPDTFDVREKWPK---------CAA--LVSEAVD----Q-G-A--C---------

OlPap-9 ----RLPTSFDARVAYPK---------CSR--LLGAVRD----Q-G-R--C---------

OtPap-8 ----SLPRHFDARDEYPK---------CAR--LIGTVRD----Q-G-K--C---------

VcPap-12 ----LLPPSKDLAQNWPV---------KRN-------------Q------C--------N

CrPap-9 ----DLPRDWD----WRNISDPRYVGSGGPRNYLSPVTNMHAPA-G-G--C---------

CrPap-6 ----DLPREWD----WRAVPSSANSDDRHN--YLSIIRN----Q-HIPKYC---------

VcPap-4 ----ELPATWD----WRN----ISGPNNGVRNFLSIVRN----Q-HIPEYC---------

OlPap-7 ---------------------------------------------G-K------------

OtPap-7 ----SLPDDFT----WSN------VKGHNF---LTKSLN----Q-HLPQYC---------

CrPap-10 ---------WN----WCN---------VDGVSYCVANWN----Q-HIPYYC---------

VcPap-6 ----DLPKNFN----WCN-------LDGTN--YCTANWN----Q-HIPYYC---------

CrPap-8 ----VPPDTVD----WRS---------SGV---LPPVTT----Q-G-E--C---------

CrPap-11 ----VPPDTVD----WRQ---------SAS---LPPVTN----Q-R-D--C---------

SmPap-19 ----DLPKHFDAREAWPQ---------CSS---IKNILD----Q-G-H--C---------

PpPap-10 ----EFDARKQ----WSH---------CPT---IGDILG----Q-G-H--C---------

PpPap-9 ----EFDARKH----WGH---------CST---IGAILD----Q-G-H--C---------

PpPap-11 ----EFDARKH----WSH---------CST---IGDILD----Q-G-H--C---------

AtPap-32 ----ILNNVLL----WST---------ITL---W-FWFL----L-G-H--C---------

AtPap-29 ----EFDARTA----WSQ---------CTS---IGRILD----Q-G-H--C---------

AtPap-30 ----AFDARTA----WPQ---------CTS---IGNILD----Q-G-H--C---------

PtPap-1 ----RLPEEFDARTAWPQ---------CST---IGKILD----Q-G-H--C---------

PtPap-2 ----KLPIEFDARTAWPH---------CST---IGRILD----Q-G-H--C---------

PtPap-3 ----MNPQFSN----YSV---GEFKYLLGV--KPTPGKELRGVPLG-H--C---------

HvPap-20 ----EFDARTQ----WSS---------CST---IGNILD----Q-G-H--C---------

HvPap-19 ----EFDARSK----WSG---------CST---IGKILD----Q-G-H--C---------

OsPap-45 ----MLPKEFDARSAWSQ---------CNT---IGTILD----Q-G-H--C---------

OlPap-5 ----SIPAAFS----WRT-----PPDGYGN--VVGVVHD----Q---EDLC---------

OtPap-5 ----GYPEQFT----WRS-----PPAGYGN--VVGLVHD----Q---QDMC---------

OlPap-6 ---------------------------------VTPVHE----E----------------

OtPap-6 ---------------------------------ISNSHE----E----------------

CrPap-5 MPSQQCPDAVD----NRR--------------FLPPIEN----Q-G-S--C---------

OlPap-4 -----FNESMD----WRK---------KID---IGPIYS----Q-G-M--C---------

OtPap-4 -------ESFD----WRK---------KID---LGPIYS----Q-G-M--C---------

HvPap-31 ----TTKGLVD----W-----------SGV--YTTPVKN----Q-G-Y--C---------

OlPap-3 ----NLPTSWD----WRK---------FGG---LSKVWE----Q-G-A--C---------

OtPap-3 ---------WD----WRD---------VGG---VGRVWE----Q-G-L--C---------

SmPap-13 ----AAPASID----WRR---------KGA--VVARVKD----I-GSS--C---------

SmPap-9 ----KSLESFD----WRE---------RGA---ITPIKN----V-G-S--C---------

CrPap-3 ----DLPPSVD----WRG---------TGA---DPGVKD----Q-G-M--C---------

VcPap-3 ----QLPYSVD----WRG---------TGA---DPGVKD----Q-G-M--C---------

CrPap-4 -----LPAEVD----WRG---------SGA--DGPGVKD----Q-V-S--C---------

VcPap-5 ----RLPTFVD----WRG---------TGA--DGPGVKD----Q-V-S--C---------

SmPap-17 ------------------------------------------------------------

HvPap-32 ----DPPAAAD----WRT---------AGV---VTPVRD----Q-GIN--C---------

SmPap-7 ----HVPEAVD----WRK---------EGA---VTSVKD----V-G-N--C---------

SmPap-11 ------PASID----WRA---------RGA---VTPVKS----L-GNN--C---------

SmPap-15 ----SVPKSID----WRT---------LGA---VTPVKD----RKG----C---------

SmPap-16 ---SDPPKSMD----WRR---------LGA---VTPVKD----RKG----C---------

HvPap-12 ----ALPETKD----WRE---------DGI---VSPVKN----Q-A-H--C---------

OsPap-40 ----ALPETKD----WRE---------DGI---VSPVKD----Q-G-H--C---------

AtPap-28 ----TVPDTKD----WRE---------DGI---VSPVKE----Q-G-H--C---------

AtPap-27 ----ALPETKD----WRE---------DGI---VSPVKD----Q-G-G--C---------

PtPap-4 ----LLPETKD----WRE---------EGI---VSPVKN----Q-G-H--C---------

PpPap-8 ----SLPKTKD----WRE---------EGI---VSQVKN----Q-A-S--C---------

SmPap-10 ----TPPSKKD----WRD---------DKI---VSPVKN----Q-Q-H--C---------

HvPap-22 ----DPPRQFD----WRE---------HGA---VTPAKQ----Q-G-A--C---------

HvPap-24 ----DLPDRVD----WRD---------KNA---VTSVKK----Q-GDY--C---------

OsPap-34 ----VPPATWD----WRL---------NGA---VTDVKD----Q-G-Q--C---------

OsPap-43 ----YTGAKVD-DTGVSG---------AGA---ASEADR----G-GAA--A---------

OsPap-42 ----DAPPVWD----WRD---------HGA---VTPVKD----Q-G-S--C---------

OsPap-44 ----DVAASWD----WRQ---------HGA---VTPARE----Q-G-T--C---------

OsPap-35 ----DVPAAWD----WRQ---------HGV---VTPVKD----Q-G-S--C---------

OsPap-37 ----DVPASWD----WRE---------HGA---VTAVKD----Q-D-G--C---------

OsPap-33 ----DAPPAWD----WRE---------HGA---VTRVKD----Q-G-P--C---------

OsPap-36 ----NVPLTWD----WRD---------SRA---VTPVKD----Q-G-P--C---------

OsPap-38 ----YVPPSWN----WTK---------YGV---VTPVKN----Q-L-T--CVNTIKMSMY

HvPap-16 -----LPPSVD----WRA---------KGV---VTPAKN----Q-GAT--C---------

SmPap-14 ----NLPEAFD----WRE---------HGA---VTPVKF----Q-G-R--C---------

SmPap-18 ----NLPKSFD----WRE---------HGA---MTPVKN----Q-G-S--C---------

OlPap-2 ----HLPEEFD----WRF---------KGA---VTRVKD----Q-G-Q--C---------

OtPap-2 ----HLPAEFD----WRF---------KGA---VSRVKD----Q-G-Q--C---------

HvPap-3 ----ALPASFD----WRD---------KGA---VTDVKM----Q-G-V--C---------

OsPap-22 ----GLPASFD----WRD---------RGA---VTDVKM----Q-G-A--C---------

AtPap-26 ----GLPEDFD----WRE---------KGG---VTEVKN----Q-G-A--C---------

PtPap-24 ----GLPDSFD----WRE---------KGA---VTDVKI----Q-G-S--C---------

PpPap-6 ----DLPPNFD----WRE---------KGA---VGPVKD----Q-G-G--C---------

PpPap-7 ----DLPPSFD----WRE---------KGA---VGPVKN----Q-G-S--C---------

SmPap-8 ----DLPEDFD----WRE---------HGA---VTEVKN----Q-G-A--C---------

HvPap-2 ----GLPTEFD----WRE---------HGA---VGPVKD----Q-G-S--C---------

OsPap-17 ----GLPDDFD----WRE---------HGA---VGPVKD----Q-G-S--C---------

HvPap-1 ----GLPEDFD----WRD---------HGA---VGPVKN----Q-G-S--C---------

OsPap-41 ----GLPDDFD----WRD---------HGA---VGPVKN----Q-G-S--C---------

AtPap-25 ----DLPTEFD----WRE---------QGA---VTPVKN----Q-G-M--C---------

AtPap-23 ----NLPEDFD----WRD---------HGA---VTPVKN----Q-G-S--C---------

AtPap-24 ----NLPEEFD----WRD---------RGA---VTPVKN----Q-G-S--C---------

PtPap-13 ----DLPEDFD----WRE---------KGA---VGPVKN----Q-G-S--C---------

PtPap-14 ----DLPEDFD----WRD---------KGA---VGPIKN----Q-G-S--C---------

PtPap-16 ----DLPTDYD----WRD---------HGA---VTEVKD----Q-G-S--C---------

PtPap-20 ----DLPTDFD----WRD---------HGA---VTSVKD----Q-G-S--C---------

HvPap-21 ----AVPESVD----WRA---------KGA---VTPVRN----Q-G-G--C---------

OsPap-8 ----AVWGSKD----WRD---------LGA---VTPVQD----Q-G-KNNC---------

SmPap-12 ----ATPEAVD----WRT---------RGA---VCPVRS----G-S-H--V--------M

SmPap-5 ----SLPTSLD----WRQ---------KGA---VTPIKD----Q-G-D--C---------

SmPap-6 ----SLPTSLD----WRQ---------EGA---VTPIKD----Q-G-Q--C---------

OsPap-13 ----AVPESVD----WRK---------EGA---VTPAKH----Q-G-Q--C---------

HvPap-25 -------ATID-------------------------------------------------

OsPap-27 ----DVPASVD----WRA---------QGA---VVPPKS----QTS-T--C---------

OsPap-39 ----DVPASVD----WRA---------QGA---VVPPKS----QTS-T--C---------

HvPap-15 ----SAPTSID----WRK---------RGV---VTPVKN----Q-K-Q--C---------

HvPap-13 ----GAPASVD----WRE---------RGA---VTAVKN----Q-G-Q--C---------

OsPap-26 ----GAPASVD----WRA---------SGA---VTEVKD----Q-G-R--C---------

OsPap-7 ----DVPDSVD----WRA---------RGA---VTEVKN----Q-R-S--C---------

OsPap-2 ----WMPCCID----WRF---------KGA---VTGVKD----Q-G-A--C---------

OsPap-10 ----WLPCCID----WRY---------KGA---VTDVKD----Q-G-A--C---------

OsPap-5 ----WTPCCID----WRF---------RGA---VTGVKD----Q-G-A--C---------

OsPap-6 ----WTPCCID----WRF---------RGA---VTGVKD----Q-G-A--C---------

HvPap-28 ----RKPCCVD----WRS---------QGA---VTGVKF----Q-G-T--C---------

OsPap-1 ----WLPCCVD----WRS---------SGA---VTGVKF----Q-R-S--C---------

OsPap-3 ----WLPCCVD----WRS---------SGA---VTGVKL----Q-G-S--C---------

OsPap-4 ----WQPCCVD----WRS---------SGA---VTGVKF----Q-G-N--C---------

HvPap-18 ----FKPCCVD----WRS---------SGA---VTGVKH----Q-G-T--C---------

HvPap-27 ----WKPCCVD----WRS---------SGA---VTGIKF----Q-G-S--C---------

HvPap-29 ----WKPCCVD----WRS---------SGA---VTGVKF----Q-G-S--C---------

OsPap-21 ----DVPANIN----WRD---------RGA---VTQVKN----Q-K-D--C---------

OsPap-23 ----QQPAEVD----WRQ---------QGA---VTGVKN----Q-R-S--C---------

AtPap-31 ----MEDESKD----WRD---------EGA---VTPVKY----Q-G-A--C---------

AtPap-21 ----DNGESMD----WRQ---------EGA---VTPVKY----Q-G-R--C---------

AtPap-22 ----ETGESMD----WIQ---------EGA---VTSVKH----Q-Q-Q--C---------

AtPap-19 ----VLGTNKD----WRN---------EGA---VTPVKS----Q-G-E--C---------

AtPap-18 ----MVVESKD----WRA---------EGA---VTPVKY----Q-G-Q--C---------

AtPap-20 ----AGRETKD----WRY---------EGA---VTPVKY----Q-G-Q--C---------

OlPap-1 ----EPLENVD----WVE---------RGA---VTAPKN----Q-G-Q--C---------

OtPap-1 ----DPPEAID----WVE---------LGA---VTPPKN----Q-G-Q--C---------

SmPap-4 ----DLPASVD----WRK---------HGA---VTAPKD----Q-G-S--C---------

AtPap-17 ----NVPDAVD----WRT---------QGA---VTPIRN----Q-G-K--C---------

CrPap-2 ----QTPAAVD----WRA---------KNA---VTQVKN----Q-G-Q--C---------

VcPap-2 ----DNPKAID----WRE---------KGA---VAEVKN----Q-G-Q--C---------

AtPap-7 ----VLPKSVD----WRN---------EGA---VTEVKD----Q-G-H--C---------

AtPap-8 ----VLPKSVD----WRN---------EGA---VTEVKD----Q-G-L--C---------

AtPap-6 ----VLPDEVD----WRE---------RGA--VVPRVKR----Q-G-E--C---------

AtPap-9 ----QLPESVD----WRQ---------EGA---VSEIKD----Q-G-T--C---------

CrPap-1 -----LPPAID----WRK---------KNA---VAEVKN----Q-G-Q--C---------

VcPap-1 ----QLPTEID----WRK---------KGA---VTEVKN----Q-G-Q--C---------

SmPap-2 ---ADTATSVD----WRK---------KGG---VTEIKD----Q-G-D--C---------

PtPap-30 ----DAPSSLD----WRN---------KGV---VTAVKD----Q-G-D--C---------

PtPap-21 ----DAPSSLD----WRK---------KGV---VTAVKD----Q-G-D--C---------

PtPap-23 ----DAPSSLD----WRK---------KGV---VTAVKD----Q-G-D--C---------

AtPap-16 ----ALPVSVD----WRK---------KGA---VTPIKN----Q-G-S--C---------

HvPap-17 ----DAAGSMD----WRA---------MGA---VTGVKD----Q-G-S--C---------

OsPap-12 ----AAPQSMD----WRA---------MGA---VTGVKD----Q-G-S--C---------

PtPap-5 ----AIPTSMD----WRK---------AGA---VTPVKD----Q-G-T--C---------

PtPap-12 -------------------------------------------M------V---------

PtPap-6 ----DIPTSMD----WRN---------DGA---VTPVKD----Q-G-T--C---------

PtPap-9 ----DIPTSMD----WRN---------DGA---VTPVKD----Q-G-T--C---------

OsPap-16 ----DLPASVD----WRG---------KGA---VNPVKD----Q-G-D--C---------

PtPap-18 ----AVPATMD----WRK---------KGA---VTPIKD----Q-G-Q--C---------

PtPap-15 ----AVPATMD----WRK---------KGA---VTPIKD----Q-G-Q--C---------

PtPap-22 ----AVPATMD----WRK---------KGA---VTLIKD----Q-G-Q--C---------

PtPap-11 ----AVPSTMD----WRK---------KGA---VTPIKD----Q-G-Q--C---------

PtPap-26 ----AVPSTVD----WRK---------EGA---VTPVKD----Q-G-Q--C---------

PtPap-27 ----AVPSTVD----WRK---------EGA---VTPVKD----Q-G-Q--C---------

PtPap-28 ----AVPATMD----WRK---------KGA---VTPVKD----Q-G-Q--C---------

PtPap-36 ----AVPATMD----WRK---------KGA---VTPVKD----Q-G-Q--C---------

OsPap-25 ----ALPASVD----WRT---------KGA---VTRIKD----Q-G-Q--C---------

OsPap-15 ----TLPATVD----WRT---------KGA---VTPIKD----Q-G-Q--C---------

OsPap-14 ----ALPATVD----WRT---------KGA---VTPIKD----Q-G-Q--C---------

OsPap-32 ----ALPATMD----WRT---------KGV---VTPIKD----Q-G-Q--C---------

HvPap-8 ----AVPDALD----WRQ---------SGA---VTKVKD----Q-G-S--C---------

OsPap-29 ----AVPDAVD----WRQ---------SGA---VTKVKD----Q-G-S--C---------

AtPap-10 ----KVPDSVD----WRK---------KGA---VTNVKD----Q-G-S--C---------

PtPap-34 ----DIPASID----WRN---------KGV---VTNVKD----Q-G-S--C---------

OsPap-31 ----NLPWAVD----WRK---------KGA---VTPVKN----Q-G-E--C---------

SmPap-3 ----DLPDSID----WRE---------KGA---VVGVKD----Q-G-S--C---------

PpPap-1 ----VAEEMVD----WRK---------KGA---VSDVKD----Q-G-S--C---------

PpPap-2 ----EAEPKVD----WRL---------KGA---VTDVKD----Q-G-A--C---------

PtPap-17 ----KLPESVD----WRG---------KGA---VNPIKD----Q-G-S--C---------

PtPap-19 ----KLPESVD----WRA---------KGA---VNPIKD----Q-G-S--C---------

SmPap-1 ----NLPLDTD----WRS---------KGA---VATVKN----Q-G-A--C---------

AtPap-5 ----VLPDEVD----WRA---------NGA---VVSVKD----Q-G-N--C---------

PpPap-4 ----EAPKSID----WRE---------KGA---VTSVKD----Q-G-S--C---------

PpPap-3 ----EAPESVD----WRK---------KGA---VTTVKD----Q-G-S--C---------

PpPap-5 ----EAPESVD----WRK---------NGA---VTSVKD----Q-G-S--C---------

AtPap-3 ----EVPETVD----WRQ---------KGA---VNPIKD----Q-G-T--C---------

OsPap-24 ---------------AKQ---------K-----IILCIS------G-H--CLNCSWWSHT

AtPap-4 ----SLPDAID----WRA---------KGA---VNPVKD----Q-G-S--C---------

OsPap-19 ----ALPDSVD----WRD---------KGA--VVAPVKN----Q-G-Q--C---------

HvPap-7 ----ELPEAVD----WRE---------KGA---VAPVKN----Q-G-Q--C---------

OsPap-18 ----ELPESVD----WRE---------KGA---VAPVKN----Q-G-Q--C---------

PtPap-25 ----DLPESVD----WRE---------KGA---VAPVKD----Q-G-Q--C---------

PtPap-31 ----DLPETVD----WRE---------KGA---VAPVKD----Q-G-Q--C---------

PtPap-29 ----SLPDSVD----WRK---------EGA---VAEVKD----Q-G-G--C---------

AtPap-1 ----ELPESID----WRK---------KGA---VAEVKD----Q-G-G--C---------

AtPap-2 ----ALPDSVD----WRK---------EGA---VADVKD----Q-G-S--C---------

HvPap-6 ----ELPESVD----WRK---------KGA---VGAVKD----Q-G-G--C---------

OsPap-9 ----DLPRAVD----WRR---------KGA---VTGVKD----Q-G-K--C---------

HvPap-30 ----DLPRSVD----WRQ---------KGA---VTGVKN----Q-G-K--C---------

HvPap-10 -----LPPSVD----WRQ---------KGA---VTGVKD----Q-G-K--C---------

HvPap-11 -----LPPSVD----WRQ---------KGA---VTGVKD----Q-G-K--C---------

OsPap-11 ----SLPKEVD----WRK---------KGA---VTEVKN----Q-G-Q--C---------

OsPap-20 ----EVPKEMD----WRK---------KNA---VTEVKN----Q-G-Q--C---------

HvPap-4 ----DLPKSVD----WRK---------KGA---VTEVKN----Q-G-Q--C---------

HvPap-5 ----DLPKAVD----WRK---------KGA---VTDVKN----Q-G-Q--C---------

AtPap-15 ----DLPKSVD----WRK---------KGA---VAPVKD----Q-G-Q--C---------

PtPap-33 ----DLPKSID----WRK---------KGA---VTPVKN----Q-G-S--C---------

AtPap-14 ----AVPKSVD----WRK---------KGA---VAEVKN----Q-G-S--C---------

PtPap-32 ----SIPKSVD----WRK---------KGA---VTDVKN----Q-G-S--C---------

PtPap-35 ----SIPKSVD----WRK---------KGA---VTDVKN----Q-G-S--C---------

HvPap-14 -----VPPSVD----WRQ---------KGA---VTAVKD----Q-G-Q--C---------

OsPap-30 ----DLPAAVD----WRE---------KGA---VGAVKD----Q-G-Q--C---------

AtPap-12 ----RVPSSVD----WRE---------KGA---VTEVKN----Q-Q-D--C---------

PtPap-7 ----KLPSSVD----WRK---------NGA---VTGIKD----Q-G-K--C---------

AtPap-11 ----KLPSSVD----WRK---------KGA---VTEIKN----Q-G-K--C---------

HvPap-9 -----LPPAVD----WRQ---------KGA---VTAIKD----Q-G-Q--C---------

OsPap-28 -----LPPAVD----WRE---------RGA---VTGIKD----Q-G-Q--C---------

AtPap-13 ----TLPTSVD----WRK---------NGA---VTPVKN----Q-G-Q--C---------

PtPap-8 ----KVPTSVD----WRK---------KGA---VTAVKD----Q-G-Q--C---------

PtPap-10 ----KVPASID----WRK---------KGA---VTPVKD----Q-G-K--C---------

VcPap-14 -NSCVSFAVIAAAE-TAYALGRR------------ISAAW----VD-FSEHSLFFCDSAL

VcPap-7 -GACVAFAAAGMAE-ALIA----------------TVNNASAYDID-LSEHWLFFCST--

CrPap-7 -GSCVAFAAVASAEAAVGAGSK-------------TASNS----LD-LSEQWLFFCDGTG

VcPap-8 -GSCVSFAATAGAEAAVATALK-------------SASNS----ND-FSEQWLFFCNG--

VcPap-13 -GSCVSFAVAAMAEAAVGAVSL-------------TTVNN----ND-FSEQWLFFCNG--

VcPap-11 -GSCVAFAVTGAAEAAVAAA---------------RKTTV---NSNDYSEQWLFFCNGMS

VcPap-9 -------LAETAVA---------------------VAGNMSAANID-LSEHWLFFCNGVD

VcPap-10 -GSCVAFAATAAAE---AAV---------------ATGNNTLVNTYDFSEQWLFFCNG--

OlPap-8 -GSCWAVAPAKVMA-DRLCIA--------------TNGAV----ASHLSAMQLLSCGKLE

OtPap-9 -GSCWAVAPAKAMT-DRLCIA--------------TNGAV----NTHVSAIQLLSCNSHS

OlPap-9 -GSCWAVAATEVMN-DRLCVA--------------TDGEN----ADELSPQYALSCFDSG

OtPap-8 -GSCWAVAATEIMN-DRLCI---------------SSGGK---EVAELSPQFALSCYNSG

VcPap-12 VGACVAFASTAAME-YVARS---------------CSSSC----TL-LSPRFVHYCARVM

CrPap-9 -GSCWAHGAASVLA-DRSNI---------------QRGGA--WPAAHVSIQHLIDCSG-G

CrPap-6 -GSCWAHGASSSLADRMNIK---------------MKGAW---PGTFLSVQNIIDCGG--

VcPap-4 -GSCWAHAASSSLADRMNIK---------------QKGAW--PGVF-LSVQNIIDCGH--

OlPap-7 -----------------------------------KRAQD----VN-LAIQHILNCGT-E

OtPap-7 -GSCWAHGAMSALA-DRIQI---------------ASGKKRRQDVN-LAIQYILNCGT-E

CrPap-10 -GSCWVHGTLSAIQDRLKIM---------------KKGET--PDVM-LARQTLLNCAAFE

VcPap-6 -GGCWVHGSLSMIQ-DRLKIKKR------------AKSPD----VM-LSRQTLLNCAAFE

CrPap-8 -GSCYAHAPAACIE-ALAVMEHAYTNLTSLL----GAGGA----RALLGPEQVVQCKP--

CrPap-11 -GSCYAHAPAACIQ-SLAVLGNAYTPSALMPSSPGSAGGA----RALLGPEQVVQCKP--

SmPap-19 -GSCWAFGAVEALT-DRFCI---------------LNNEN----VS-LSENDLVACCS-S

PpPap-10 -GSCWAFGAVESLT-DRFCI---------------HLNES----VS-LSENDLLACCGFE

PpPap-9 -GSCWAFGAAESLT-DRFCI---------------HMNES----VS-LSENDLLACCGFE

PpPap-11 -GSCWAFGAVESLT-DRFCI---------------HLNES----VS-LSENDLLACCGFE

AtPap-32 -GSCWAFGAVESLS-DRFCI---------------KYNLN----VS-LSANDVIACCGLL

AtPap-29 -GSCWAFGAVESLS-DRFCI---------------KYNMN----VS-LSVNDLLACCGFL

AtPap-30 -GSCWAFGAVESLS-DRFCI---------------QFGMN----IS-LSVNDLLACCGFR

PtPap-1 -GSCWAFGAVESLS-DRFCI---------------HYGMN----IS-LSVNDLLACCGFL

PtPap-2 -GSCWAFGAVESLS-DRFCI---------------HYGMN----LS-LSVNDLLACCGWM

PtPap-3 -GSCWAFGAVESLS-DRFCI---------------HYGMN----LS-LSVNDLLACCGWM

HvPap-20 -GACWAFAAVESLQ-DRFCI---------------HLNMS----VS-LSVNDLLACCGFL

HvPap-19 -GSCWAFGAVECLQ-DRFCI---------------HHNMN----IS-LSANDLVACCGFM

OsPap-45 -GSCWAFGAVECLQ-DRFCI---------------HFNMN----IS-LSVNDLVACCGFM

OlPap-5 -ASCWAFVTADSIASRIAII---------------NKGDD----APALSVKQLMACDAVD

OtPap-5 -ASCWAFVTADSIASRIAVI---------------NKGDD----APRLSVKQLMACDDID

OlPap-6 -----------------------------------------------LAVDELINCDT--

OtPap-6 -----------------------------------------------LAVDELINCDT--

CrPap-5 -GCCWAFGATAAIEAAVGKL---------------LDTQS----FS-LSKQQVATC----

OlPap-4 -SGCWAFTTAQVVG-DSKAI---------------ATGSR----MA-VSPYHLLSCDN-L

OtPap-4 -SGCWAFSTAQVVA-DSKTI---------------ASGAR----VA-VSPHHLLSCDN-L

HvPap-31 -GSCWAFSATEQIE-SDSMR---------------TLGTS----YV-LSPEQITQCAT-K

OlPap-3 -GGCWAFTTAAAVE-GVHYI---------------WTREA----VT-LSPQMLLECDP-I

OtPap-3 -GGCWAFTTAAAVE-GVHYI---------------WTKEP----VT-LSPQMLLECDP-I

SmPap-13 KGASWTYAATGAVE-GRLAI---------------DGNRL----QD-LSAQQLLDCVDEA

SmPap-9 -LGTWAFATGDSLS-SLNKI---------------LFGGL----AN-FSAQEFLDCTK-K

CrPap-3 -GSCYTFAATGTMD-GTWFV---------------ATGQR----RS-FSEQQIIDCAW-D

VcPap-3 -GSCYAFAATGAMD-GAWFV---------------ATGQR----RS-FSEQQIVDCAW-E

CrPap-4 -GSCWAFGVVGALQ-GAWWK---------------ATGQS----LS-LSEQQLVDCAW-D

VcPap-5 -GSCWAFSAAGAMQ-GAWFK---------------ATGQA----LS-FSEQQLVDCAW-D

SmPap-17 -GGSWAFAAAGAVE-SFKAI---------------QTGRM----VV-LSPQQLIDCNT--

HvPap-32 -GSCWAFATASAIE-SLYAI---------------EHGDV----IP-LSPQQLIDCDG-S

SmPap-7 TGGGWAFATAGAVE-GLNKI---------------VTGNL----VE-LSAQELIDCDV--

SmPap-11 SDGSWAFATAAAVE-GVHYI---------------ATGQL----VD-LSAQQLLDCDT-A

SmPap-15 -QSNWAFAAAEAIE-GINKI---------------ATGVL----VE-LSAQQLLDCSR-S

SmPap-16 QNGSWAFAAAEAIE-GINKI---------------ATGVL----VE-LSAQQLLDCSR-S

HvPap-12 -GSCWTFSTTGALE-AAYTQ---------------ATGKN----IS-LSEQQLVDCAG-G

OsPap-40 -GSCWTFSTTGSLE-AAYTQ---------------ATGKP----VS-LSEQQLVDCAT-A

AtPap-28 -GSCWTFSTTGALE-AAYHQ---------------AFGKG----IS-LSEQQLVDCAG-T

AtPap-27 -GSCWTFSTTGALE-AAYHQ---------------AFGKG----IS-LSEQQLVDCAG-A

PtPap-4 -GSCWTFSTTGALE-AAYHQ---------------AFGKG----IS-LSEQQLVDCAR-A

PpPap-8 -GSCWTFSTTGALE-AAHAQ---------------ATGKM----VL-LSEQQLVDCAG-E

SmPap-10 -GSCWTFSTTGALE-SAHAQ---------------ATGKM----VV-LSEQQLVDCAG-G

HvPap-22 -GCCWAFAAAATVE-SLNKI---------------NGGEL----VD-LSVQELVDCST-G

HvPap-24 -GSCWAFTAVGAVE-GITAI---------------KTGKL----ED-LSPQMLIDCDK-D

OsPap-34 -GSCWVFSAVGAVE-GINAI---------------MTGNL----LT-LSEQQVLDCSN-T

OsPap-43 -GSCWAFSAVGAVE-GINAI---------------MTGNL----LT-LSEQQVLDCFG-A

OsPap-42 -GSCWAFSAVGAVE-GVNAI---------------ATGNL----LR-LSEQQLLDCTNPN

OsPap-44 -ESCWAFSAVGAVE-GANAI---------------ATGKL----VT-LSEQQVLDCSG-A

OsPap-35 -GSCWAFSSVGAVE-SAYAI---------------ATKKL----LR-LSEQQVLDCSG-G

OsPap-37 -GSCWAFSAVGAVE-SINAI---------------ATGNL----LT-LSEQQVLDCSG-D

OsPap-33 -GSCWAFSVVEAVE-GINAI---------------MTGNL----LT-LSEQQVLDCSG-A

OsPap-36 -GSCWAFSVVGAVE-SINKI---------------RTGIL----LT-LSEQQVLDCSG-A

OsPap-38 EGSCWAFSVAAAVE-SINMI---------------RTGNL----LT-LSEQQILDCSG-A

HvPap-16 -FSCWAFTSVATME-SAQAI---------------STGGS----PPVLSEQQLVDCST-L

SmPap-14 -GSCWTFSTTGVVE-GANFL---------------KTGKL----IS-LSEEQLIDCDY--

SmPap-18 -GSCWTFSSTGAVE-GAHFL---------------KSREL----IS-LREEQLVDCDR--

OlPap-2 -GSCWTFSTTGAIE-GAHFI---------------STGKL----VE-LSEQQLVDCDV-G

OtPap-2 -GSCWTFSTTGAIE-GAHFI---------------STGKL----VE-LSEQQLLDCDV-G

HvPap-3 -GSCWAFSTTGAVE-GANFV---------------ATGKL----LD-LSEQQLVDCDH-T

OsPap-22 -GSCWAFSTTGAVE-GANFL---------------ATGNL----LD-LSEQQLVDCDH-T

AtPap-26 -GSCWAFSTTGAAE-GAHFV---------------STGKL----LS-LSEQQLVDCDQAV

PtPap-24 -GSCWAFSTTGSVE-GANFI---------------ATGKL----LN-LSEQQLVDCDR-V

PpPap-6 -GSCWAFSTTGAVE-GAHFL---------------NSGKL----VS-LSEQQLVDCDH-Q

PpPap-7 -GSCWAFSTTGAVE-GAHFL---------------ATGKL----LS-LSEQQLVDCDH-Q

SmPap-8 -GSCWAFSTTGAIE-GAHFL---------------ETGKL----IS-LSEQQLVDCDH-S

HvPap-2 -GSCWSFSTSGALE-GANYL---------------ATGKL----EV-LSEQQLVDCDH-E

OsPap-17 -GSCWSFSTSGALE-GAHFL---------------ATGKL----EV-LSEQQMVDCDH-E

HvPap-1 -GSCWSFSASGALE-GANYL---------------ASGKM----EV-LSEQQLVDCDH-E

OsPap-41 -GSCWSFSASGALE-GANYL---------------ATGKM----DV-LSEQQMVDCDH-E

AtPap-25 -GSCWSFSAIGALE-GAHFL---------------ATKEL----VS-LSEQQLVDCDH-E

AtPap-23 -GSCWSFSATGALE-GANFL---------------ATGKL----VS-LSEQQLVDCDH-E

AtPap-24 -GSCWSFSTTGALE-GAHFL---------------ATGKL----VS-LSEQQLVDCDH-E

PtPap-13 -GSCWSFSTTGALE-GAHFL---------------ATGEL----VS-LSEQQLVDCDH-E

PtPap-14 -GSCWSFSATGALE-GAHFL---------------ATGEL----VS-LSEQQLVDCDH-E

PtPap-16 -GSCWSFSATGALE-GAHYL---------------ATGEL----AS-LSEQQLVDCDH-E

PtPap-20 -GSCWSFSATGALE-GAHYL---------------ATGEL----VS-LSEQQLVDCDH-E

HvPap-21 -EASVAFAALAAVE-GLYQI---------------KTGKL----VS-MSVQELVDCDS-L

OsPap-8 -NSCWAFAVVAATE-GLIKI---------------ETGNV----TP-LSAQQVLDCTG-G

SmPap-12 TGSSVVFSAAAAAE-GANAI---------------ATGEL----KC-LFEEAFFKCSK-N

SmPap-5 -GSCWAFSAIASIE-SAHFL---------------ATKEL----VS-LSEQQLMDCDT-V

SmPap-6 -GSCWAFSAIASIE-SAHFL---------------ATKEL----VS-LSEQQLIDCDT-V

OsPap-13 -AACWAFAAVAAIE-SLHKI---------------KGGDL----IS-LSEQELVDCDD-T

HvPap-25 ---------------TLNWI---------------KTGKL----VA-LSEQQLVDCDH--

OsPap-27 -SSCWAFVTAATIE-SLNMI---------------KTGKL----VS-LSEQQLVDCDS--

OsPap-39 -------STTATIE-SLNMI---------------KTRRL----VS-LSEQQLVDCDS--

HvPap-15 -GSCWAFPTVATIE-GIHKI---------------KRGTL----VS-LSEQQLIDCDY-L

HvPap-13 -GSCWAFSTVAVIE-GIHQI---------------QTGKL----AS-LSEQELVDCDK-L

OsPap-26 -GSCWAFSTVAVVE-GIQKI---------------KKGKL----VS-LSEQELVDCDT-L

OsPap-7 -GSCWAFAAVAATE-GLVQL---------------ATGNL----VS-LSEQQVLDCTG-G

OsPap-2 -GSSWAFAAVAAME-GLMKI---------------RTGQL----TP-LSEQELVDCVD-G

OsPap-10 -GSCWAFAAVAAIE-GLTQI---------------RTGKL----TP-LSEQELVDCDT-G

OsPap-5 -GSCWAFAAVAAIE-GLTKI---------------RTGQL----TP-LSEQELVDCDT-N

OsPap-6 -GSCWAFAAVAAIE-GLTKI---------------RTGQL----TP-LSEQELVDCDT-N

HvPap-28 -LSCWAFAAVAAIE-GMNKI---------------RTGEL----VS-LSEQQLVDCDT-R

OsPap-1 -ASCWAFAAAAAIE-GLNKI---------------RTGEL----VS-LSEQVMVDCDT-G

OsPap-3 -ASCWAFAAVAAIE-GLHRI---------------KTGEL----VS-LSEQVMVDCDT-G

OsPap-4 -ASCWAFASAAAIE-GLHKI---------------KTGEL----VS-LSEQVMVDCDT-G

HvPap-18 -GSCWAFAAVAAIE-GMNKI---------------RTGEL----VS-LSEQVLVDCDT-V

HvPap-27 -LSCWAFAAVAAIE-GMNKI---------------RTGEL----VS-LSEQQLVDCDT-G

HvPap-29 -LSCWAFAAVAAIE-GMNKI---------------RTGEL----VS-LSEQQLVDCDT-G

OsPap-21 -ASCWAFSAVAAVE-GIHQI---------------RSHNL----VA-LSTQQLLDCST-G

OsPap-23 -GCCWAFSTVAAVE-GIHQI---------------TTGEL----VS-LSEQQLLDCAD--

AtPap-31 ---------------RLTKI---------------SGKNL----LT-LSEQQLIDCDI-E

AtPap-21 -GGCWAFSAVAAVE-GITKI---------------TKGEL----VS-LSEQQLLDCDR-D

AtPap-22 -GCCWAFSAVAAVE-GMTKI---------------ANGEL----VS-LSEQQLLDCST-E

AtPap-19 -GGCWAFSAIAAVE-GLTKI---------------ARGNL----IS-LSEQQLLDCTR-E

AtPap-18 -GCCWAFSAVAAVE-GVAKI---------------AGGNL----VS-LSEQQLLDCDR-E

AtPap-20 -GCCWAFSSVAAVE-GLTKI---------------VGNNL----VS-LSEQQLLDCDR-E

OlPap-1 -GSCWAFSTTGAIE-GINQI---------------RTGRL----VS-LSEQELVSCST-Q

OtPap-1 -GSCWAFSTTGAVE-GITKI---------------RTGRL----VS-LSEQEMVSCSK-Q

SmPap-4 -GGCWAFATTGAIE-GINQI---------------VTGQL----MS-LSEQELIDCDK-K

AtPap-17 -GGCWAFSAVAAIE-GINKI---------------KTGNL----VS-LSEQQLIDCDV-G

CrPap-2 -GSCWAFSAVGSIE-GANAL---------------ATGQL----VA-LSEQQLVDCDT-A

VcPap-2 -GSCWAFSTTGAIE-GINAI---------------VTGQL----QS-LSEQQLVDCDT-G

AtPap-7 -RSCWAFSTVGAVE-GLNKI---------------VTGEL----VT-LSEQDLINCNK-E

AtPap-8 -RSCWAFSTVGAVE-GLNKI---------------VTGEL----VT-LSEQDLINCNK-E

AtPap-6 -GSCWAFAATGAVE-GINQI---------------TTGEL----VS-LSEQELIDCDR-G

AtPap-9 -NSCWAFSTVAAVE-GLNKI---------------VTGEL----IS-LSEQELVDCNL-V

CrPap-1 -GSCWAFATTGSVE-GINAI---------------VTGSL----VS-LSEQELVDCDT-E

VcPap-1 -GSCWAFATTGSVE-GINAI---------------VTGEL----AS-LSEQELVDCDT-D

SmPap-2 -GSCWAFSAVAAVE-GLTFL---------------STGTL----VS-LSEQELVDCDT-T

PtPap-30 -GSCWSFSTTGAIE-AINAI---------------VTGDL----IS-LSEQELVDCDT-T

PtPap-21 -GSCWSFSTTGAIE-GINAI---------------VTSDL----IS-LSEQELVDCDT-T

PtPap-23 -GSCWSFSTTGAIE-GINAI---------------VTGDL----IS-LSEQELVDCDT-T

AtPap-16 -GCCWAFSAVAAIE-GATQI---------------KKGKL----IS-LSEQQLVDCDT-N

HvPap-17 -GCCWAFSAVAAVE-GLTKI---------------RTGRL----VS-LSEQQLVDCDV-Y

OsPap-12 -GCCWAFSAVAAVE-GLAKI---------------RTGQL----VS-LSEQELVDCDV-R

PtPap-5 -GCCWAFSAVAAIE-GIIKL---------------KTGKL----IS-LSEQQLVDCDV-K

PtPap-12 -GCCWAFSAVAAIE-GIIKL---------------KTGNL----IS-LSKQQLVNRDV-G

PtPap-6 -GCCWAFSTVAAIE-GIIKL---------------QTGNL----IS-LSEQQLVDCTA-G

PtPap-9 -GCCWAFSTVAAIE-GIIKL---------------QTGNL----IS-LSEQQLVDCTA-G

OsPap-16 -GCCWAFSAVAAME-GAVKL---------------ATGKL----VS-LSEQQLVSCDV-K

PtPap-18 -GSCWAFSTVAATE-GINQL---------------TTGKL----VS-LSEQELVDCDT-Q

PtPap-15 -GSCWAFSTVAATE-GINQL---------------TTGKL----VS-LSEQELVDCDN-Q

PtPap-22 -GSCWAFSTVAATE-GINQL---------------TTGKL----VS-LSEQELVDCDI-Q

PtPap-11 -GCCWAFSAVAATE-GITQL---------------STGKL----IS-LSEQELVDCDT-S

PtPap-26 -GCCWAFSAVAAME-GINKL---------------TTGKL----IS-LSEQEVVDCDT-K

PtPap-27 -GCCWAFSAVAAME-GINKL---------------TTGKL----IS-LSEQEVVDCDT-K

PtPap-28 -GCCWAFSAVAAME-GINQL---------------TTGKL----IS-LSEQEVVDCDT-K

PtPap-36 ---------VAAME-GINQL---------------TTGKL----IS-LSEQEVVDCDT-K

OsPap-25 -GCCWAFSAVAAME-GFVKL---------------STGKL----IS-LSEQELVDCDV-D

OsPap-15 -GCCWAFSAVAAME-GIVKL---------------STGKL----IS-LSEQELVDCDV-H

OsPap-14 -GCCWAFSAVAAME-GIVKL---------------STGKL----IS-LSEQELVDCDV-H

OsPap-32 -GCCWAFSAVAAME-------------------------------------ELVDCDV-H

HvPap-8 -GACWSFSATGAME-GINKI---------------TTGSL----LS-LSEQELIDCDR-S

OsPap-29 -GACWSFSATGAME-GINKI---------------KTGSL----IS-LSEQELIDCDR-S

AtPap-10 -GACWSFSATGAME-GINQI---------------VTGDL----IS-LSEQELIDCDK-S

PtPap-34 -GACWSFSATGAIE-GINKI---------------VTGSL----VS-LSEQELIECDK-S

OsPap-31 -GSCWAFSTVAAVE-GINQI---------------VTGKL----VS-LSEQELMDCDN--

SmPap-3 -GSCWAFSAVAAIE-GVNKL---------------ATGEL----VS-LSEQELVDCDK-G

PpPap-1 -GSCWAFSAIGSVE-GVNAI---------------VTGEL----IS-LSEQELVDCDR-G

PpPap-2 -GSCWAFSAVGSVE-GVNAI---------------KTGEL----VS-LSEQELVDCDR-K

PtPap-17 -GSCWAFSTVAAVE-GINQI---------------VTGEL----IS-LSEQELVDCDR-F

PtPap-19 -GSCWAFSTVAAVE-GINQI---------------VTGEL----IS-LSEQELVDCDR-T

SmPap-1 -GSCWAFSTVAAVE-GVNQI---------------VTGEL----VS-LSEQELVDCDK-Q

AtPap-5 -GSCWAFSAVGAVE-GINQI---------------TTGEL----IS-LSEQELVDCDR-G

PpPap-4 -GSCWAFSAVGSVE-GINAI---------------RTGDA----IS-LSVQELVDCDK-K

PpPap-3 -GSCWAFSAIGSVE-GINAI---------------RTGEA----VS-LSEQELVDCDL-E

PpPap-5 -GSCWAFSAVGSVE-GINAI---------------RNGEA----VS-LSEQELVDCDL-E

AtPap-3 -GSCWAFSTTAAVE-GINKI---------------VTGEL----IS-LSEQELVDCDK-S

OsPap-24 SGSCWAFSAIAAVE-GINQI---------------VTGDL----IS-LSEQELVDCDT-S

AtPap-4 -GSCWAFSAIGAVE-GINQI---------------KTGEL----IS-LSEQELVDCDT-S

OsPap-19 -GSCWAFSAVAAVE-GINKI---------------VTGEL----VS-LSEQELVECAR-N

HvPap-7 -GSCWAFSAVSTVE-SINQI---------------VTGEM----VT-LSEQELVECDI-N

OsPap-18 -GSCWAFSAVSTVE-SINQL---------------VTGEM----IT-LSEQELVECST-N

PtPap-25 -GSCWAFSTVGAVE-GINQI---------------VTGNL----TS-LSEQELVDCDK-V

PtPap-31 -GSCWAFSTVGAVE-GINQI---------------VTGNL----TS-LSEQELVDCDK-T

PtPap-29 -GSCWAFSTIAAVE-GINKI---------------VTGDL----IA-LSEQELVDCDT-S

AtPap-1 -GSCWAFSTIGAVE-GINQI---------------VTGDL----IT-LSEQELVDCDT-S

AtPap-2 -GSCWAFSTIGAVE-GINKI---------------VTGDL----IS-LSEQELVDCDT-S

HvPap-6 -GSCWAFSAIAAVE-GINQI---------------VTGDM----IP-LSEQELVDCDT-S

OsPap-9 -GSCWAFSTVVSVE-GINAI---------------RTGRL----VS-LSEQELIDCDT-A

HvPap-30 -GSCWAFSTVVSVE-GINAI---------------RTGKL----VS-LSEQELIDCDT-A

HvPap-10 -GSCWAFSTVVSVE-GINAI---------------RTGSL----VS-LSEQELIDCDT-A

HvPap-11 -GSCWAFSTVVSVE-GINAI---------------RTGSL----VS-LSEQELIDCDT-A

OsPap-11 -GSCWAFSTVAAVE-GINAI---------------VTGNL----TR-LSEQELIDCDT-D

OsPap-20 -GSCWAFSTVAAVE-GINAI---------------VTGNL----TS-LSEQELIDCST-D

HvPap-4 -GSCWAFSTVAAVE-GINAI---------------VTGNL----TA-LSEQELIDCSV-D

HvPap-5 -GSCWAFSTVAAVE-GINAI---------------VTGNL----TA-LSEQELIDCSV-D

AtPap-15 -GSCWAFSTVAAVE-GINQI---------------TTGNL----SS-LSEQELIDCDT-T

PtPap-33 -GSCWAFSTVAAVE-GINQI---------------VAGNL----TS-LSEQQLIDCDT-S

AtPap-14 -GSCWAFSTVAAVE-GINKI---------------VTGNL----TT-LSEQELIDCDT-T

PtPap-32 -GSCWAFSTVAAVE-GINQI---------------VTGNL----TS-LSEQELVDCDTTN

PtPap-35 -GSCWAFSTVAAVE-GINQI---------------VTGNL----TS-LSEQELVDCDT-T

HvPap-14 -GSCWAFSTIAAVE-GINAI---------------RTKNL----TS-LSEQQLVDCDT-K

OsPap-30 -GSCWAFSTIAAVE-GINAI---------------RTSNL----TA-LSEQQLVDCDT-K

AtPap-12 -GSCWAFSTVAAVE-GINKI---------------RTNKL----VS-LSEQELVDCDT-E

PtPap-7 -GSCWAFSTVAAVE-GINKI---------------KTGEL----IS-LSEQELVDCDS-D

AtPap-11 -----------AVE-GINKI---------------KTNKL----VS-LSEQELVDCDTKQ

HvPap-9 -GSCWAFSTIVAVE-GINKI---------------RTGKL----VS-LSEQELMDCDNVN

OsPap-28 -GSCWAFSTVAAVE-GVNKI---------------KTGRL----VT-LSEQELVDCDT-G

AtPap-13 -GSCWAFSTVVAVE-GINQI---------------RTKKL----TS-LSEQELVDCDT-N

PtPap-8 -GSCWAFSTIVAVE-GINYI---------------KTNEL----VS-LSEQELVDCDTTE

PtPap-10 -GSCWAFSTIVAVE-GINFI---------------KTNKL----IS-LSEQELVDCNT-G

VcPap-14 ---------------------------AASPPNC-NY-GWNV-PDAL-A--ALQR--SG-

VcPap-7 -------------------------SSG---PLC-EE-GWYA-SSAV----TVIA--NQS

CrPap-7 ---------------------------S---PSC-GG-GWYA-S-AA-T--SVIV--SKN

VcPap-8 -------------------------LYN---PTC-DT-GWTA-DEAA-D--VLAK--FS-

VcPap-13 -------------------------MFV---PSC-EY-GWFARD-AT----TVVV--NKN

VcPap-11 ---------------------------SAAFPNC-DS-GWFA-T-AA-A--KVVV--TKN

VcPap-9 --------------------------TA---ASC-DN-GWYA-TSAT-KALAVKT-----

VcPap-10 -------------------------LYT---PSC-ST-GWYA-NQAA-D--VIVK--KNM

OlPap-8 -----------------NGTFDAGSTYS---GSC-DG-GFPN-E-AY-EKARTSGIVSGG

OtPap-9 --------------NSAYTYDENLAGGS---GGC-MG-GYPT-E-AY-ETAHRVGVVSGG

OlPap-9 -------------------------------SGC-DG-GDVL-D-TL-RIAFTKGIPYGG

OtPap-8 -------------------------------AGC-EG-GDVV-D-TL-TLALAKGVPHGG

VcPap-12 ------------------------TTGSEPSDDT-AG-THIS-T-AM-A--ALRM--FGA

CrPap-9 -------------------------------GSCRDG-GDEV-A-AY-K--YAAE--TGV

CrPap-6 ---------------------------A---GSC-NG-GDDR-L-VY-V--YGAK--HGI

VcPap-4 ---------------------------A---GSC-NG-GDDR-M-VY-V--YANK--HGI

OlPap-7 --------------------------IA---GSC-HG-GSHT-G-AY-Q--FVHD--TGF

OtPap-7 --------------------------VA---GSC-HG-GSHT-G-AY-Q--FVKD--SGF

CrPap-10 -------------------------GYG---NGC-DG-GDTV-D-VF-G--YMTD--FGL

VcPap-6 -------------------------GYG---HGC-DG-GDTV-D-VF-S--YMAE--FGL

CrPap-8 -------------------------------GGC-AG-GWVG-D----T--YIQA--MRG

CrPap-11 -------------------------------GGC-AG-GWVG-D----T--YIQA--MRG

SmPap-19 --------------------------CG---FGC-DG-GYPY-A-AW-E--YFAQ--TGV

PpPap-10 --------------------------CG---YGC-EG-GYPI-R-AW-K--YFKH--SGV

PpPap-9 --------------------------CG---DGC-DG-GYPI-R-AW-R--YFKR--TGV

PpPap-11 --------------------------CG---DGC-EG-GYPI-R-AW-Q--YFKR--TGV

AtPap-32 --------------------------CG---FGC-NG-GFPM-G-AW-L--YFKY--HGV

AtPap-29 --------------------------CG---QGC-NG-GYPI-A-AW-R--YFKH--HGV

AtPap-30 --------------------------CG---DGC-DG-GYPI-A-AW-Q--YFSY--SGV

PtPap-1 --------------------------CG---SGC-NG-GYPI-S-AW-R--YFVH--HGV

PtPap-2 --------------------------CG---AGC-DG-GSPI-D-AW-R--YFVQ--SGV

PtPap-3 --------------------------CG---DGC-DG-GYPI-D-AW-R--YFVQ--SGV

HvPap-20 --------------------------CG---SGC-NG-GYPI-S-AW-R--YFRR--SGV

HvPap-19 --------------------------CG---DGC-DG-GYPI-S-AW-Q--YFVQ--NGV

OsPap-45 --------------------------CG---DGC-DG-GYPI-M-AW-R--YFVR--NGV

OlPap-5 -------------------------------HGC-ST-GNMY-T-AY-E--WIGQ--YGG

OtPap-5 -------------------------------HAC-ST-GNMY-T-AY-E--WLGQ--HGG

OlPap-6 --------------------------YD---SGCATG-NMFT---AF-E--WIET--KGG

OtPap-6 --------------------------YD---SGCATG-NMFT---AF-E--WIET--VGG

CrPap-5 ---------------------------------------------LY-------------

OlPap-4 ---------------------------D---SAC-NT-GNMA-T-AY-A--WINVQPKG-

OtPap-4 ---------------------------D---SAC-NT-GNMA-T-AY-S--WINVQPKG-

HvPap-31 ---------------------------A---YGC-DG-GWTE-V-AY-A--YVKN--AGG

OlPap-3 ---------------------------D---QDC-TG-GNMV-T-GY-Q--YAVM--KGG

OtPap-3 ---------------------------D---QDC-VG-GNMV-T-GY-Q--YAVM--KGG

SmPap-13 -------------------------------NKC----TTLD-P-VF-ALDYVAE--NG-

SmPap-9 ---------------------------A---KGC-RS-GSPS-D-AF-E--YART--HS-

CrPap-3 -------------------------YGP---NGC-FG-GYYQ-P-VL-N--YVAE--QGG

VcPap-3 -------------------------YGP---NGC-FG-GYYQ-P-VF-N--YVAE--NGG

CrPap-4 -------------------------YGN---DGC-MG-GYIE-P-TL-A--YVID--GGG

VcPap-5 -------------------------YGN---DGC-AG-GFVE-P-TL-Q--YVVD--AGG

SmPap-17 --------------------------YN---SGC-NEKGTADPYHAL-N--YIRL--HG-

HvPap-32 ---------------------------N---RNC-RG-GNPA-E-AF-Q--TIKL--GDG

SmPap-7 --------------------------YN---GGC-DY-GFPQ-D-SF-A--YIQK--TG-

SmPap-11 -------------------------YGN---SGC-SK-GFPQ-N-SF-P--YLEE--GAG

SmPap-15 -------------------------YGT---DGC-HS-GFPQ-N-AF-E--YLVD--TGN

SmPap-16 -------------------------YGT---DGC-HS-GFPQ-N-AF-D--YLDD--TGN

HvPap-12 -------------------------FNN---FGC-NG-GLPS-Q-AF-E--YIKY--NGG

OsPap-40 -------------------------YNN---FGC-SG-GLPS-Q-AF-E--YIKY--NGG

AtPap-28 -------------------------FNN---FGC-HG-GLPS-Q-AF-E--YIKY--NGG

AtPap-27 -------------------------FNN---YGC-NG-GLPS-Q-AF-E--YIKS--NGG

PtPap-4 -------------------------FNN---FGC-NG-GLPS-Q-AF-E--YIKF--NGG

PpPap-8 -------------------------FNN---FGC-GG-GLPS-Q-AF-E--YIRY--NGG

SmPap-10 -------------------------YNN---FGC-NG-GLPS-Q-AF-E--YIRY--NGG

HvPap-22 -------------------------VFS---SPC--GYGWPK-S-AL-Q--WIKS--KGG

HvPap-24 ---------------------------N---RGC-RC-GMVW-R-AF-D--FIKK--NG-

OsPap-34 -------------------------------GDCLKG-GDPR-A-AL-Q--YIVK--NGV

OsPap-43 -------------------------------GDC-SG-GWPD-Q-AQ-Q--YIVK--NGI

OsPap-42 -------------------------------DDCING-GRAE-R-AM-Q--YVVN--NGI

OsPap-44 -------------------------------GDCIGG-GSYF-P-VLHG--YAVK--QG-

OsPap-35 -------------------------------GDC-GG-GYTS-T-VLSE--FAVK--KGI

OsPap-37 -------------------------------GDC-NG-GWPN-L-VLSG--YAVE--QGI

OsPap-33 -------------------------------GDC-SG-GYTS-Y-AF-D--YAVS--NGI

OsPap-36 -------------------------------GDC-VF-GYPK-D-AF-N--HIVN--TGV

OsPap-38 -------------------------------GDC-NG-GYPY-D-AF-D--YVIK--TGI

HvPap-16 ---------------------------H---HGC-GR-GWMD-D-AF-K--WVIM--NGG

SmPap-14 --------------------------KD---NGC-EG-GDML-S-AY-E--YV-K--ARG

SmPap-18 --------------------------MD---GGC-KG-GDML-N-AY-E--YI-K--AKG

OlPap-2 ------------------CDPDVPNACD---SGC-NG-GLPS-N-AM-E--YIVE--HGG

OtPap-2 ------------------CDPDVPNACD---SGC-NG-GLPS-N-AM-E--YIVE--HGG

HvPap-3 ------------------CDAVAKTECN---SGC-SG-GLMT-N-AY-R--YLMS--SGG

OsPap-22 ------------------CDAEKKTECD---SGC-GG-GLMT-N-AY-A--YLMS--SGG

AtPap-26 ------------------CFPKDKKACD---NGC-GG-GLMT-N-AY-E--YLME--AGG

PtPap-24 ------------------CDKTDKASCD---DGC-GG-GLMT-N-AY-R--YLIE--AGG

PpPap-6 ------------------CDREEADACD---AGC-NG-GFMT-N-AY-Q--YVEA--AGG

PpPap-7 ------------------CDPEEAQACD---AGC-GG-GLMT-N-AY-K--YVEE--AGG

SmPap-8 ------------------CDPTDKVSCD---AGC-NG-GLMT-N-AY-D--YVMK--SGG

HvPap-2 ------------------CDPSEPRACD---AGC-NG-GLMT-T-AF-S--YLAK--AGG

OsPap-17 ------------------CDASESRACD---SGC-NG-GLMT-T-AF-S--YLMK--SGG

HvPap-1 ------------------CDPSEPDSCD---AGC-NG-GLMT-S-AF-S--YLLK--SGG

OsPap-41 ------------------CDSSEPDSCD---AGC-NG-GLMT-N-AF-S--YLLK--SGG

AtPap-25 ------------------CDPAQANSCD---SGC-SG-GLMN-N-AF-E--YALK--AGG

AtPap-23 ------------------CDPEEADSCD---SGC-NG-GLMN-S-AF-E--YTLK--TGG

AtPap-24 ------------------CDPEEEGSCD---SGC-NG-GLMN-S-AF-E--YTLK--TGG

PtPap-13 ------------------CDPEEPGSCD---SGC-NG-GLMN-S-AF-E--YTLK--AGG

PtPap-14 ------------------CDPEEPGSCD---SGC-NG-GLMN-S-AF-E--YTLK--AGG

PtPap-16 ------------------CDPEEYGACD---SGC-DG-GLMN-N-AF-E--YALK--AGG

PtPap-20 ------------------CDPEEYGACD---SGC-SG-GLMN-N-AF-E--YALK--AGG

HvPap-21 ---------------------------S---THC-NPGGTPA-A-AL-S--YIQR--NGG

OsPap-8 ---------------------------D---NTC-KG-GHIH-E-AL-R--YIATASAGG

SmPap-12 ---------------------------D---L------GHYD-K-AF-E--FIIE--NGG

SmPap-5 ---------------------------D---AGC-DG-GLME-T-AF-K--FVVK--NGG

SmPap-6 ---------------------------D---QGC-QG-GFPE-D-AF-K--FVVE--NGG

OsPap-13 --------------------------GE---ATC-SK-GYSD-D-AF-L--WVSK--NKG

HvPap-25 --------------------------YD---GGC-NK-GSFH-R-AY-N--WIVE--NGG

OsPap-27 --------------------------YD---GGC-NL-GSYG-R-AY-K--WVVE--NGG

OsPap-39 --------------------------YD---GGC-NL-GSYG-R-AY-K--WVVE--NGG

HvPap-15 ---------------------------D---NGC-KG-GLVT-R-AF-Q--WIKK--NGG

HvPap-13 ---------------------------D---HGC-NG-GVSY-R-AL-Q--WITS--NGG

OsPap-26 ---------------------------D---SGC-DG-GVSY-R-AL-E--WITA--NGG

OsPap-7 ---------------------------A---NTC-SG-GDVS-A-AL-R--YIAA--SGG

OsPap-2 ------------------------GGDS---DGC-GG-GHTD-A-AF-Q--LVVD--KGG

OsPap-10 ---------------------------S---SGC-AG-GHTD-R-AF-E--LVAA--KGG

OsPap-5 ---------------------------S---NGC-GG-GHTD-R-AF-E--LVAS--KGG

OsPap-6 ---------------------------S---NGC-GG-GHTD-R-AF-E--LVAS--KGG

HvPap-28 ---------------------------S---SGC-SG-GRSD-T-AM-G--LAPXGRQST

OsPap-1 ---------------------------S---SGC-SG-GRAD-T-AL-G--LVAA--RGG

OsPap-3 ---------------------------S---NGC-GG-GRSD-T-AL-G--LVAS--RGG

OsPap-4 ---------------------------S---FGC-SG-GHSD-T-AL-N--LVAS--RGG

HvPap-18 ---------------------------S---TGC-GG-GHSD-S-AM-A--LVAA--RGG

HvPap-27 ---------------------------S---SGC-SG-GRSD-T-AL-G--LAAT--RGG

HvPap-29 ---------------------------S---SGC-SG-GRTD-T-AL-G--LVAA--RGG

OsPap-21 -------------------------RNN---HGC-NR-GDMD-E-AF-R--YITS--NGG

OsPap-23 ---------------------------N---GGC-TG-GSLD-N-AF-Q--YMAN--SGG

AtPap-31 --------------------------KN---GGC-NG-GEFE-E-AF-K--YIIK--NGG

AtPap-21 --------------------------YN---QGC-RG-GIMS-K-AF-E--YIIK--NQG

AtPap-22 ---------------------------N---NGC-GG-GIMW-K-AF-D--YIKE--NQG

AtPap-19 --------------------------QN---NGC-KG-GTFV-N-AF-N--YIIK--HRG

AtPap-18 --------------------------YD---RGC-DG-GIMS-D-AF-N--YVVQ--NRG

AtPap-20 --------------------------RD---NGC-NG-GIMS-D-AF-S--YIIK--NRG

OlPap-1 ---------------------------N---MAC-NG-GLMD-N-AF-K--WVQK--NGG

OtPap-1 ---------------------------N---MGC-NG-GLMD-Y-AF-R--WIVK--NGG

SmPap-4 --------------------------AD---KGC-DG-GLME-N-AY-Q--FIVE--NGG

AtPap-17 -------------------------TYN---KGC-SG-GLME-T-AF-E--FIKT--NGG

CrPap-2 --------------------------SN---MGC-SG-GLMD-D-AF-K--YVLD--NGG

VcPap-2 KRTVTRSKRSCTVILPSYSSNSCRNESN---MGC-SG-GLMD-D-AF-K--YVIQ--NGG

AtPap-7 ---------------------------N---NGC-GG-GKLE-T-AY-E--FIMK--NGG

AtPap-8 ---------------------------N---NGC-GG-GKVE-T-AY-E--FIMN--NGG

AtPap-6 -------------------------NDN---FGC-AG-GGAV-W-AF-E--FIKE--NGG

AtPap-9 ---------------------------N---NGC-YGSGLMD-T-AF-Q--FLIN--NNG

CrPap-1 --------------------------QD---KGC-SG-GLMD-Y-AY-A--WIIK--NKG

VcPap-1 --------------------------ED---RGC-SG-GLMD-Y-AY-Q--WIIK--NGG

SmPap-2 --------------------------VN---QGC-DG-GIMD-Y-AF-Q--YMIR--NGG

PtPap-30 -------------------------N-N---YGC-EG-GDMD-S-AF-Q--WVIG--NGG

PtPap-21 ---------------------------N---YGC-EG-GYMD-Y-AF-E--WVIN--NGG

PtPap-23 ---------------------------N---YGC-EG-GYMD-Y-AF-E--WVIN--NGG

AtPap-16 ---------------------------D---FGC-EG-GLMD-T-AF-E--HIKA--TGG

HvPap-17 -------------------------GDD---EGC-AG-GLMD-N-AF-E--YMIN--RGG

OsPap-12 -------------------------GED---QGC-EG-GLMD-T-AF-Q--YIAR--RGG

PtPap-5 -------------------------GVD---QGC-GG-GLMD-N-AF-Q--FILR--NGG

PtPap-12 ---------------------------N---KGC-HG-GLMD-T-AF-Q--YIIR--NEG

PtPap-6 ---------------------------N---KGC-QG-GLMD-T-AF-Q--YIIR--NGG

PtPap-9 ---------------------------N---KGC-QG-GLMD-T-AF-Q--YIIR--NGG

OsPap-16 -------------------------GED---QGC-EG-GLMD-D-AF-D--FIIK--NGG

PtPap-18 -------------------------GED---QGC-EG-GLME-D-GF-E--FIIK--NHG

PtPap-15 -------------------------GED---QGC-EG-GLME-D-GF-E--FIIK--NHG

PtPap-22 -------------------------GED---QGC-EG-GLME-D-GF-E--FIIK--NHG

PtPap-11 -------------------------GED---QGC-NG-GLMD-D-AF-D--FIIQ--NKG

PtPap-26 -------------------------GED---QGC-NG-GLMD-D-AF-K--FIEQ--NKG

PtPap-27 -------------------------GED---QGC-NG-GLMD-D-AF-K--FIEQ--NKG

PtPap-28 -------------------------GED---QGC-NG-GLMD-D-AF-K--FIEQ--NKG

PtPap-36 -------------------------GED---QGC-NG-GLMD-D-AF-K--FIEQ--NKG

OsPap-25 -------------------------GND---QGC-EG-GEID-G-AF-Q--FILS--NGG

OsPap-15 -------------------------GED---QGC-EG-GLMD-D-AF-K--FIIK--NGG

OsPap-14 -------------------------GED---QGC-EG-GLMD-D-AF-K--FIIK--NGG

OsPap-32 -------------------------GED---QGC-EG-GLMD-D-AF-K--FIIK--NGG

HvPap-8 --------------------------YN---TGC-GG-GLMT-Y-AY-K--FVIK--NGG

OsPap-29 --------------------------YN---SGC-GG-GLMD-Y-AY-K--FVVK--NGG

AtPap-10 --------------------------YN---AGC-NG-GLMD-Y-AF-E--FVIK--NHG

PtPap-34 --------------------------YN---DGC-GG-GLMD-Y-AF-Q--FVIN--NHG

OsPap-31 -------------------------TFN---HGC-RG-GLMD-F-AF-A--YIMG--NQG

SmPap-3 --------------------------ED---EGC-NG-GLMD-Y-AF-G--FVIK--NGG

PpPap-1 --------------------------QN---QGC-NG-GLMD-Y-AF-D--FIIK--NGG

PpPap-2 --------------------------QN---QGC-NG-GLMD-Y-AF-E--FIIK--NGG

PtPap-17 --------------------------YN---AGC-NG-GLMD-Y-AF-Q--FIIN--NGG

PtPap-19 --------------------------YN---AGC-NG-GLMD-Y-AF-Q--FIIN--NGG

SmPap-1 --------------------------KN---QGC-NG-GLMD-S-AF-E--FIIQ--NGG

AtPap-5 -------------------------FVN---AGC-DG-GIMN-Y-AF-E--FIMK--NGG

PpPap-4 --------------------------YN---QGC-NG-GLMD-Y-AF-D--FVIQ--NGG

PpPap-3 --------------------------YN---QGC-NG-GLMD-Y-AF-D--FILE--NGG

PpPap-5 --------------------------YN---QGC-NG-GLMD-Y-AF-D--FIIQ--NGG

AtPap-3 --------------------------YN---QGC-NG-GLMD-Y-AF-Q--FIMK--NGG

OsPap-24 --------------------------YN---EGC-NG-GLMD-Y-AF-D--FIIN--NGG

AtPap-4 --------------------------YN---DGC-GG-GLMD-Y-AF-K--FIIE--NGG

OsPap-19 -------------------------GQN---SGC-NG-GIMD-D-AF-A--FIAR--NGG

HvPap-7 -------------------------GQS---SGC-NG-GLMD-D-AF-E--FIIK--NGG

OsPap-18 -------------------------GQN---SGC-NG-GLMD-D-AF-D--FIIK--NGG

PtPap-25 --------------------------YN---QGC-NG-GLMD-Y-AF-E--FIMK--NGG

PtPap-31 --------------------------YN---LGC-NG-GLMD-Y-AF-D--FIIE--NGG

PtPap-29 --------------------------YN---EGC-NG-GLMD-Y-AF-E--FIIN--NGG

AtPap-1 --------------------------YN---EGC-NG-GLMD-Y-AF-E--FIIK--NGG

AtPap-2 --------------------------YN---QGC-NG-GLMD-Y-AF-E--FIIK--NGG

HvPap-6 --------------------------YN---QGC-NG-GLMD-Y-AF-E--FIIN--NGG

OsPap-9 -------------------------D-N---SGC-QG-GLME-N-AF-E--YIKH--SGG

HvPap-30 -------------------------D-N---DGC-EG-GLMD-N-AF-E--YIQK--NGG

HvPap-10 -------------------------D-N---DGC-QG-GLMD-N-AF-E--YIKN--NGG

HvPap-11 -------------------------D-N---DGC-QG-GLMD-N-AF-E--YIKN--NGG

OsPap-11 -------------------------G-N---NGC-SG-GLMD-Y-AF-S--YIAA--NGG

OsPap-20 -------------------------G-N---NGC-NG-GLMD-Y-AF-S--YIAS--TGG

HvPap-4 -------------------------G-N---SGC-NG-GLMD-Y-AF-S--YIAS--SGG

HvPap-5 -------------------------G-N---SGC-NG-GMMD-Y-AF-S--YIAS--SGG

AtPap-15 --------------------------FN---SGC-NG-GLMD-Y-AF-Q--YIIS--TGG

PtPap-33 --------------------------FN---NGC-NG-GLMD-Y-AF-E--FIVN--NGG

AtPap-14 --------------------------YN---NGC-NG-GLMD-Y-AF-E--YIVK--NGG

PtPap-32 ---------------------------N---YGC-NG-GLMD-Y-AF-S--YIIS--NGG

PtPap-35 --------------------------YN---NGC-NG-GLMD-Y-AF-A--YIIS--NGG

HvPap-14 -------------------------S-N---AGC-NG-GLMD-Y-AF-Q--YIAK--HGG

OsPap-30 -------------------------TGN---AGC-DG-GLMD-N-AF-Q--YIAK--HGG

AtPap-12 -------------------------E-N---QGC-AG-GLME-P-AF-E--FIKN--NGG

PtPap-7 ---------------------------N---HGC-NG-GLME-D-AF-N--FIKQ--IGG

AtPap-11 ---------------------------N---EGC-NG-GLME-I-AF-E--FIKK--NGG

HvPap-9 ---------------------------N---QGC-EG-GLMD-Y-AF-Q--FIQK--N-G

OsPap-28 --------------------------DN---QGC-DG-GLMD-Y-AF-Q--FIKR--NGG

AtPap-13 --------------------------QN---QGC-NG-GLMD-L-AF-E--FIKE--KGG

PtPap-8 ---------------------------N---QGC-NG-GLME-Y-AF-E--FIKK--KRG

PtPap-10 --------------------------EN---HGC-NG-GLMD-Y-AF-E--FITK--QKG

VcPap-14 -V-Y---LDCC-------R--------------E-YNPTL--------------------

VcPap-7 -I-P---FEFN-------Y--------------P-Y-RPD--------------------

CrPap-7 -L-P---QEPF-------Y--------------P-Y-KAA--------------------

VcPap-8 -I-P---QETN-------Y--------------P-Y-KGT--------------------

VcPap-13 -I-P---YEVN-------F--------------P-Y-LGI--------------------

VcPap-11 -I-P---YELN-------Y--------------P-Y-TGS--------------------

VcPap-9 -I-P---YEAN-------Y--------------P-Y-NPS--------------------

VcPap-10 ---P---YEKN-------Y--------------P-YIQSP--------------------

OlPap-8 LFGD---DKTC-------M--------------P-Y-AFA--------------------

OtPap-9 LNGD---QDTC-------M--------------P-YPFAP--------------------

OlPap-9 ML-D---SNAC-------L--------------P-Y-EFE--------------------

OtPap-8 ML-D---KGAC-------L--------------P-Y-QFE--------------------

VcPap-12 -------APEV-------YTYIVWAAA---TLCP-H-SAP--------------------

CrPap-9 ---P---PETC-------S--------------P-YVAMD--------------------

CrPap-6 ---P---PDTC-------N--------------L-Y-VAA--------------------

VcPap-4 ---P---PDTC-------N--------------L-Y-VAH--------------------

OlPap-7 -V-P---YDTC-------L--------------P-Y-EAC--------------------

OtPap-7 -V-P---YDTC-------L--------------P-Y-EAC--------------------

CrPap-10 ---P---DEGC-------M--------------T-Y-NAT--------------------

VcPap-6 ---P---DEGC-------M--------------T-Y-NAT--------------------

CrPap-8 LL-----YQEE-------WAKAVALTTGAVSGAP-SANGT--------------------

CrPap-11 LL-----YQEE-------WAKAVAAAG------P-D-AST--------------------

SmPap-19 -V-T---SQCD----------------------P-YFDGK--------------------

PpPap-10 -V-T---NKCD----------------------P-YFDQK--------------------

PpPap-9 -V-T---SKCD----------------------P-YFDQI--------------------

PpPap-11 -V-----TSKC-------D--------------P-YFDQK--------------------

AtPap-32 -V-T---QECD----------------------P-YFDNT--------------------

AtPap-29 -V-T---EECD----------------------P-YFDNT--------------------

AtPap-30 -V-T---EECD----------------------P-YFDNT--------------------

PtPap-1 -V-T---EECD----------------------P-YFDDI--------------------

PtPap-2 -V-T---EECD----------------------P-YFDDI--------------------

PtPap-3 -V-T---EECD----------------------P-YFDDI--------------------

HvPap-20 -V-T---EECD----------------------P-YFDQT--------------------

HvPap-19 -V-T---EECD----------------------P-YFDQV--------------------

OsPap-45 -V-----TDEC-------D--------------P-YFDQV--------------------

OlPap-5 -I-S---SKAD-------Y--------------NAKVPGD--------------------

OtPap-5 -I-S---TMED-------Y--------------NEKVPGE--------------------

OlPap-6 -I-A---TNKN-------F--------------NGLLDGI--------SKKPESWFSKVQ

OtPap-6 -L-S---GNDK-------F--------------ASVLADVGSRPLNWFTSDEFKTKARSN

CrPap-5 -------STAQ-------Y--------------P-F-TGL--------------------

OlPap-4 -V-L---VATD-------F--------------P-E------------------------

OtPap-4 -V-L---LASD-------F--------------P-E------------------------

HvPap-31 -I-V---TEAN-------YPYT-----------S-F-AGK--------------------

OlPap-3 -I-S---AAED-------YPVH-----------P-Y-TIK--------------------

OtPap-3 -I-S---AAGD-------YPVH-----------P-Y-TLK--------------------

SmPap-13 -L-T---TEKL-------Y--------------P-Y-TGT--------------------

SmPap-9 -I-A---AAAD-------Y--------------P-F-VGE--------------------

CrPap-3 -M-A---LEQD-------Y--------------T-Y-RGE--------------------

VcPap-3 -I-A---LEQD-------Y--------------S-Y-RSE--------------------

CrPap-4 -I-A---QEAD-------Y--------------P-Y-LAQ--------------------

VcPap-5 -I-A---QESE-------Y--------------P-Y-LAQ--------------------

SmPap-17 -I-T---TDSK-------Y--------------P-Y-TGV--------------------

HvPap-32 -I-S---RWVD-------Y--------------P-Y-TGA--------------------

SmPap-7 -L-E---ASAS-------Y--------------P-Y-IGK--------------------

SmPap-11 -L-H---KEAD-------Y--------------P-F-TGS--------------------

SmPap-15 GL-S---IASD-------Y--------------H-F-TGF--------------------

SmPap-16 GL-S---TASD-------Y--------------R-F-TGF--------------------

HvPap-12 -I-D---TEES-------Y--------------P-Y-KGV--------------------

OsPap-40 -L-D---TEEA-------Y--------------P-Y-TGV--------------------

AtPap-28 -L-D---TEEA-------Y--------------P-Y-TGK--------------------

AtPap-27 -L-D---TEKA-------Y--------------P-Y-TGK--------------------

PtPap-4 -L-D---TEEA-------Y--------------P-Y-TGK--------------------

PpPap-8 -I-D---TEDS-------Y--------------P-Y-NAK--------------------

SmPap-10 -L-D---TEDS-------Y--------------P-Y-TGH--------------------

HvPap-22 -L-L---TEAE-------Y--------------P-Y-VAK--------------------

HvPap-24 -I-A---TERA-------Y--------------P-Y-DGI--------------------

OsPap-34 TL-DQCGKLPY-------Y--------------PGY-EAK--------------------

OsPap-43 TL-DRCGKEPY-------Y--------------PAY-DAT--------------------

OsPap-42 AL-DASCPGPY-------Y--------------PPY-EAE--------------------

OsPap-44 -I-S---PAGS-------Y--------------PPY-EAK--------------------

OsPap-35 AL-DASGNPPY-------Y--------------PPY-QAK--------------------

OsPap-37 AL-DNIGDPAY-------Y--------------PPY-VAK--------------------

OsPap-33 TL-DQCFSPPTTGENYFYY--------------PAY-EAV--------------------

OsPap-36 SL-DSRGKPPY-------Y--------------PPY-EAQ--------------------

OsPap-38 SL-DNRGNPPY-------Y--------------PPY-ENQ--------------------

HvPap-16 -I-T---TEAA-------Y--------------P-Y-TGK--------------------

SmPap-14 -L-E---ADED-------Y--------------P-Y-EEL--------------------

SmPap-18 -L-E---AEED-------Y--------------P-Y-QEE--------------------

OlPap-2 -I-D---TEKS-------Y--------------P-Y-VGE--------------------

OtPap-2 -I-D---TEKS-------Y--------------P-Y-VGE--------------------

HvPap-3 -L-M---EQAA-------Y--------------P-Y-TGA--------------------

OsPap-22 -L-M---EQSA-------Y--------------P-Y-TGA--------------------

AtPap-26 -L-E---EERS-------Y--------------P-Y-TGK--------------------

PtPap-24 -L-Q---EESS-------Y--------------P-Y-TGK--------------------

PpPap-6 -L-E---LESD-------Y--------------P-Y-EGR--------------------

PpPap-7 -L-E---LESD-------Y--------------P-Y-KGR--------------------

SmPap-8 -L-E---TETD-------Y--------------P-Y-TGN--------------------

HvPap-2 -L-E---TEKD-------Y--------------P-Y-TGR--------------------

OsPap-17 -L-Q---SEKD-------Y--------------P-Y-AGR--------------------

HvPap-1 -L-E---REKD-------Y--------------P-Y-TGK--------------------

OsPap-41 -L-E---SEKD-------Y--------------P-Y-TGR--------------------

AtPap-25 -L-M---KEED-------Y--------------P-Y-TGR--------------------

AtPap-23 -L-M---KEED-------Y--------------P-Y-TGK--------------------

AtPap-24 -L-M---REKD-------Y--------------P-Y-TGT--------------------

PtPap-13 -L-M---REED-------Y--------------P-Y-TGM--------------------

PtPap-14 -L-M---REED-------Y--------------P-Y-TGT--------------------

PtPap-16 -L-E---REED-------Y--------------P-Y-TGT--------------------

PtPap-20 -L-E---REKD-------Y--------------P-Y-TGN--------------------

HvPap-21 -I-A---AAAD-------Y--------------P-Y-TAQ--------------------

OsPap-8 RL-S---TDKS-------YR-------------P-Y-DGE--------------------

SmPap-12 -I-D---SE-G-------F--------------G-L-NFR--------------------

SmPap-5 -V-T---TEAA-------Y--------------P-Y-TGS--------------------

SmPap-6 -V-T---TEEA-------Y--------------P-Y-TGF--------------------

OsPap-13 -I-A---SDLI-------Y--------------P-Y-VGH--------------------

HvPap-25 -I-T---TAAE-------Y--------------P-Y-KAV--------------------

OsPap-27 -L-T---TEAD-------Y--------------P-Y-TAR--------------------

OsPap-39 -L-T---TEAD-------Y--------------P-Y-TAR--------------------

HvPap-15 -I-T---STSS-------Y--------------K-Y-KAV--------------------

HvPap-13 -I-T---SQDD-------Y--------------P-Y-TAK--------------------

OsPap-26 -I-T---TRDD-------Y--------------P-Y-TAA--------------------

OsPap-7 -L-Q---TEAA-------Y--------------A-Y-GGQ--------------------

OsPap-2 -I-T---AESE-------Y--------------R-Y-EGY--------------------

OsPap-10 -I-T---AESG-------Y--------------R-Y-EGY--------------------

OsPap-5 -I-T---AESD-------Y--------------R-Y-EGF--------------------

OsPap-6 -I-T---AESD-------Y--------------R-Y-EGF--------------------

HvPap-28 -V-Q---REEG-------T--------------P-Y-SGF--------------------

OsPap-1 -V-A---SEEE-------Y--------------P-Y-TGV--------------------

OsPap-3 -V-T---SEER-------Y--------------P-Y-AGA--------------------

OsPap-4 -I-T---SEEK-------Y--------------P-Y-TGV--------------------

HvPap-18 -I-T---SEER-------Y--------------P-Y-AGF--------------------

HvPap-27 -I-T---SEAK-------Y--------------P-Y-SGI--------------------

HvPap-29 -I-T---SEER-------Y--------------P-Y-SGF--------------------

OsPap-21 -I-A---AESD-------Y--------------P-YEDRA--------------------

OsPap-23 -V-T---TEAA-------Y--------------A-Y-QGA--------------------

AtPap-31 -V-S---LETE-------Y--------------P-Y-QVK--------------------

AtPap-21 -I-T---TEDN-------Y--------------P-YQESQ--------------------

AtPap-22 -I-T---TEDN-------Y--------------P-Y-QGA--------------------

AtPap-19 -I-S---SENE-------Y--------------P-Y-QVK--------------------

AtPap-18 -I-A---SEND-------Y--------------S-Y-QGS--------------------

AtPap-20 -I-A---SEAS-------Y--------------P-Y-QAA--------------------

OlPap-1 -I-D---SEFQ-------Y--------------P-Y-AAE--------------------

OtPap-1 -I-D---SEFQ-------Y--------------P-Y-SAE--------------------

SmPap-4 -L-D---TETD-------Y--------------P-Y-HAS--------------------

AtPap-17 -L-A---TETD-------Y--------------P-Y-TGI--------------------

CrPap-2 -I-D---TEED-------Y--------------S-YWSGG--------------------

VcPap-2 -L-D---TEQD-------Y--------------A-YWSGY--------------------

AtPap-7 -L-G---TDND-------Y--------------P-Y-KAV--------------------

AtPap-8 -L-G---TDND-------Y--------------P-Y-KAL--------------------

AtPap-6 -I-V---SDEV-------Y--------------G-Y-TGE--------------------

AtPap-9 -L-D---SEKD-------Y--------------P-Y-QGT--------------------

CrPap-1 -I-N---TEED-------Y--------------P-Y-TAM--------------------

VcPap-1 -L-D---TEDD-------Y--------------P-Y-TAE--------------------

SmPap-2 -I-T---SQSN-------Y--------------P-Y-RAL--------------------

PtPap-30 -I-D---TEAD-------Y--------------P-Y-TGV--------------------

PtPap-21 -I-D---TEAN-------Y--------------P-Y-TGV--------------------

PtPap-23 -I-D---TEAN-------Y--------------P-Y-TGV--------------------

AtPap-16 -L-T---TESN-------Y--------------P-Y-KGE--------------------

HvPap-17 -L-T---TESS-------Y--------------P-Y-RGT--------------------

OsPap-12 -L-A---AESS-------Y--------------P-Y-RGV--------------------

PtPap-5 -L-T---SEAT-------Y--------------P-Y-QGV--------------------

PtPap-12 -L-T---SEDN-------Y--------------P-Y-QGV--------------------

PtPap-6 -L-T---SEDN-------Y--------------P-Y-QGV--------------------

PtPap-9 -L-T---SEDN-------Y--------------P-Y-QGV--------------------

OsPap-16 -L-A---AESD-------Y--------------P-Y-TAS--------------------

PtPap-18 -I-T---TEAN-------Y--------------P-Y-QAA--------------------

PtPap-15 -I-T---TEAN-------Y--------------P-Y-QAA--------------------

PtPap-22 -I-T---TEAN-------Y--------------P-Y-QAA--------------------

PtPap-11 -L-T---TEAN-------Y--------------P-Y-QGA--------------------

PtPap-26 -L-T---TEAN-------Y--------------P-Y-KGT--------------------

PtPap-27 -L-T---TEAN-------Y--------------P-Y-KGT--------------------

PtPap-28 -L-T---TEAN-------Y--------------P-Y-TGT--------------------

PtPap-36 -L-T---TEAN-------Y--------------P-Y-TGT--------------------

OsPap-25 -L-T---AEAN-------Y--------------P-Y-TAE--------------------

OsPap-15 -L-T---TESK-------Y--------------P-Y-TAA--------------------

OsPap-14 -L-T---TESN-------Y--------------P-Y-AAA--------------------

OsPap-32 -L-T---TESN-------Y--------------P-Y-AAV--------------------

HvPap-8 -I-D---TEDD-------Y--------------P-F-REA--------------------

OsPap-29 -I-D---TEAD-------Y--------------P-Y-RET--------------------

AtPap-10 -I-D---TEKD-------Y--------------P-Y-QER--------------------

PtPap-34 -I-D---TEED-------Y--------------P-Y-RAR--------------------

OsPap-31 -I-Y---TEED-------Y--------------P-Y-LME--------------------

SmPap-3 -L-D---TEAD-------Y--------------P-Y-KGY--------------------

PpPap-1 -I-D---TEED-------Y--------------P-Y-KAT--------------------

PpPap-2 -I-D---TEKD-------Y--------------P-Y-KAR--------------------

PtPap-17 -L-D---TEKD-------Y--------------P-Y-LGN--------------------

PtPap-19 -L-D---TEKD-------Y--------------P-Y-VGD--------------------

SmPap-1 -L-D---SEAD-------Y--------------P-Y-KAV--------------------

AtPap-5 -I-E---TDQD-------Y--------------P-Y-NAN--------------------

PpPap-4 -I-D---TEKD-------Y--------------P-Y-QGY--------------------

PpPap-3 -I-D---TEND-------Y--------------P-Y-KGL--------------------

PpPap-5 -I-D---TEKD-------Y--------------P-Y-KGF--------------------

AtPap-3 -L-N---TEKD-------Y--------------P-Y-RGF--------------------

OsPap-24 -I-D---TEDD-------Y--------------P-Y-KGK--------------------

AtPap-4 -I-D---TEED-------Y--------------P-Y-IAT--------------------

OsPap-19 -L-D---TEED-------Y--------------P-Y-TAM--------------------

HvPap-7 -I-D---TEDD-------Y--------------P-Y-KAV--------------------

OsPap-18 -I-D---TEDD-------Y--------------P-Y-KAV--------------------

PtPap-25 -I-D---TEED-------Y--------------P-Y-KAV--------------------

PtPap-31 -I-D---TEED-------Y--------------P-Y-KAI--------------------

PtPap-29 -I-D---TEDD-------Y--------------P-Y-LGR--------------------

AtPap-1 -I-D---TDKD-------Y--------------P-Y-KGV--------------------

AtPap-2 -I-D---TEAD-------Y--------------P-Y-KAA--------------------

HvPap-6 -I-D---SEED-------Y--------------P-Y-KER--------------------

OsPap-9 -I-T---TESA-------Y--------------P-Y-RAA--------------------

HvPap-30 -L-T---TEAA-------Y--------------P-Y-RAA--------------------

HvPap-10 -L-I---TEAA-------Y--------------P-Y-RAA--------------------

HvPap-11 -L-I---TEAA-------Y--------------P-Y-RAA--------------------

OsPap-11 -L-H---TEES-------Y--------------P-Y-LME--------------------

OsPap-20 -L-R---TEEA-------Y--------------P-Y-AME--------------------

HvPap-4 -L-H---TEEA-------Y--------------P-Y-LME--------------------

HvPap-5 -L-H---TEEA-------Y--------------P-Y-LME--------------------

AtPap-15 -L-H---KEDD-------Y--------------P-Y-LME--------------------

PtPap-33 -L-H---KEED-------Y--------------P-Y-LME--------------------

AtPap-14 -L-R---KEED-------Y--------------P-Y-SME--------------------

PtPap-32 -L-H---KEVD-------Y--------------P-Y-IME--------------------

PtPap-35 -L-H---KEED-------Y--------------P-Y-IME--------------------

HvPap-14 -V-A---AEDA-------Y--------------P-Y-KAR--------------------

OsPap-30 -V-A---ASSA-------Y--------------P-Y-RAR--------------------

AtPap-12 -I-K---TEET-------Y--------------P-Y-DSS--------------------

PtPap-7 -L-T---SENT-------Y--------------P-Y-RAK--------------------

AtPap-11 -I-T---TEDS-------Y--------------P-Y-EGI--------------------

HvPap-9 -I-T---TESN-------Y--------------P-Y-QGE--------------------

OsPap-28 -I-T---TESN-------Y--------------P-Y-RAE--------------------

AtPap-13 -L-T---SELV-------Y--------------P-Y-KAS--------------------

PtPap-8 -I-T---TEST-------Y--------------P-Y-KAE--------------------

PtPap-10 -I-T---TEAN-------Y--------------P-Y-RAQ--------------------

VcPap-14 -------------------------------GSSKC-------ESPA-------------

VcPap-7 -------------------------------APS-C-------KLLS-------------

CrPap-7 ----------P--------------------T-T-C-------TPTS-------------

VcPap-8 ----------K----------------------T-C-------VLNA------------K

VcPap-13 ----------P--------------------G---C-------TLRS-------------

VcPap-11 ----------R--------------------G---C-------ALGS-------------

VcPap-9 ----------V--------------------P-T-C-------TLHS-------------

VcPap-10 ----------S------------------------C-------TLMS-------------

OlPap-8 ---------------------------------P-CQHPC--NPNHVAQCPTTC-RNKNV

OtPap-9 -------------------------------CHHPC-------EPNHNAVCPRTCQRSAT

OlPap-9 -------------------------------A---C------------------------

OtPap-8 ---------------------------------P-CDHPCMIPGTSP-------------

VcPap-12 ----------G--------------------DFQIC-------EEPS------------K

CrPap-9 ----------H--------------------G-A-C-------TPLQ--QCYTCWPHCRP

CrPap-6 ----------N----------------------QKC-------HAMQQCFTCWPDSTCEP

VcPap-4 ----------N----------------------QQC-------HDKEQCYTCWPDEGCIP

OlPap-7 ----------SAESTEGNCARGGDYTCTAMNTCRTCSTFAEFGGFCS------------A

OtPap-7 ----------SKESTEGNCGNGGDYTCTAMNTCRTCSTFSEWGGFCS------------A

CrPap-10 ----------D---------------HTKFPGHKRC-------PAHG-------------

VcPap-6 ----------D---------------HTKFPGVSHC-------PVEG-------------

CrPap-8 -----------------------------------C-------PVATIQRLWTDLNSTAL

CrPap-11 -----------------------------------C-------PKAPLDKLASDLDSANI

SmPap-19 -------------------------------G---CKHPGCEPEYDT-PVCVKQCVDNEQ

PpPap-10 -------------------------------G---CAHPGCYPTYET-PKCEKQCVDDEF

PpPap-9 -------------------------------G---CGHPGCYPTYRT-PKCVKHCVDDEL

PpPap-11 -------------------------------G---CGHPGCYPTYDT-PKCFKRCVDDEL

AtPap-32 -------------------------------G---CSHPGCEPTYPTPKCERKCVSRNQL

AtPap-29 -------------------------------G---CSHPGCEPAYPTPKCARKCVSGNQL

AtPap-30 -------------------------------G---CSHPGCEPAYPTPKCSRKCVSDNKL

PtPap-1 -------------------------------G---CSHPGCEPGYPTPKCARKCVNKNQL

PtPap-2 -------------------------------G---CSHPGCEPGFPTPKCERKCADKNKL

PtPap-3 -------------------------------G---CSHPGCEPGFPTPKCERKCADKNKL

HvPap-20 -------------------------------G---CQHPGCEPAYPTPKCHRKCKVENQV

HvPap-19 -------------------------------G---CKHPGCEPAYPTPVCEKKCKVQNQV

OsPap-45 -------------------------------G---CKHPGCEPAYPTPVCEKKCKVQNQV

OlPap-5 -----RDDAPD----------------------AKC-------DASV-------------

OtPap-5 -------REDD--------------------PEAQC-------AAEA-------------

OlPap-6 AHAGPIGYANDDLAPEFSTRPEPHFESALKVE-PGV-------TLEK--VQEQMCMASEK

OtPap-6 ELQDEFEAMPDPHFETMPQTKPGVTLEKIQEA-A-C-------SSAV------------R

CrPap-5 ----------G-----------------IAPGPAAC-------RTSS-------------

OlPap-4 -------------------------------G-SSC-------EVVK------------S

OtPap-4 -------------------------------G-SSC-------EVAN-------------

HvPap-31 ----------T--------------------G-T-C-------AVNT-------------

OlPap-3 ----------T-----------------SEVG-P-C-------RSNT-------------

OtPap-3 ----------T-----------------SEVG-P-C-------RTNT-------------

SmPap-13 ----------I--------------------G-Q-C-------KPLP-------------

SmPap-9 ----------A--------------------G-A-C-------KHKE-------------

CrPap-3 ----------P--------------------G-Y-C-----RWGASN------------H

VcPap-3 ----------V--------------------G-F-C-----R--SAN------------R

CrPap-4 ----------N----------------------S-C-------KGGN------------K

VcPap-5 ----------N--------------------S-F-CNDVTQRGGPAT------------V

SmPap-17 ----------K--------------------S-S-C-------KPFV-------------

HvPap-32 ----------K--------------------G-W-C-------YPAR-------------

SmPap-7 ----------N--------------------S-T-C---------HI-------------

SmPap-11 ----------S--------------------G-S-C-------KKKD-------------

SmPap-15 ----------N--------------------A-T-C-----RKHVST------------C

SmPap-16 ----------N--------------------G-T-C-------RKHV-------------

HvPap-12 ----------N--------------------G-V-C-------HYKA------------E

OsPap-40 ----------N--------------------G-I-C-------HYKP------------E

AtPap-28 ----------D--------------------G-G-C-------KFSA------------K

AtPap-27 ----------D--------------------E-T-C-------KFSA------------E

PtPap-4 ----------D--------------------D-A-C-------KFSS------------E

PpPap-8 ----------D--------------------S-Q-C-------RFHK------------N

SmPap-10 ----------D--------------------G-K-C-------TYNQ------------N

HvPap-22 ----------R--------------------G-R-C-------KVHD-------------

HvPap-24 ----------E----------------------HRC-------YMKS-------------

OsPap-34 ----------K--------------------L-A-C-------RTVA-------------

OsPap-43 ----------K----------------------HPC-------RTVA-------------

OsPap-42 ----------K--------------------L-P-C-------RTEP-------------

OsPap-44 ----------D----------------------RAC-------RRNT-------------

OsPap-35 ----------K----------------------LAC-------RTVA-------------

OsPap-37 ----------K----------------------MAC-------RTVA-------------

OsPap-33 ----------Q--------------------E-P-C-------RFDP-------------

OsPap-36 ----------K--------------------K-Q-C-------RFDL-------------

OsPap-38 ----------K--------------------Q-K-C-------RFDP-------------

HvPap-16 ----------A--------------------G-N-C-------QTGK-------------

SmPap-14 ---GYRHKPVR--------------------G-P-C-------RYQP-------------

SmPap-18 ----------N--------------YKEYMFPHHRC-------HFRP-------------

OlPap-2 ----------K--------------------G-E-C-------KAKK------------G

OtPap-2 ----------K--------------------G-E-C-------KADE------------G

HvPap-3 ----------Q--------------------G-P-C-------RFDR------------G

OsPap-22 ----------Q--------------------G-T-C-------RFDA------------N

AtPap-26 ----------R--------------------G-H-C-------KFDP------------E

PtPap-24 ----------S--------------------G-E-C-------KFDP------------E

PpPap-6 ----------D--------------------G-K-C-------KFDS------------N

PpPap-7 ----------D--------------------G-K-C-------QFNP------------N

SmPap-8 ----------S-------------------NG-K-C-------QFNA------------N

HvPap-2 ----------N----------------------SAC-------KFDK-------------

OsPap-17 ----------E--------------------N-T-C-------KFDK------------S

HvPap-1 ----------D--------------------G-T-C-------KFDK------------S

OsPap-41 ----------D--------------------G-T-C-------KFDK------------S

AtPap-25 ----------D-------------------HT-A-C-------KFDK------------S

AtPap-23 ----------D-------------------GK-T-C-------KLDK------------S

AtPap-24 ----------D-------------------GG-S-C-------KLDR------------S

PtPap-13 ----------D-------------------RG-A-C-------KFDK------------N

PtPap-14 ----------D-------------------RD-A-C-------KFDK------------N

PtPap-16 ----------D-------------------GG-T-C-------KFDK------------S

PtPap-20 ----------D-------------------RG-A-C-------KFEK------------S

HvPap-21 ----------E--------------------G-V-C-------NTDV------------P

OsPap-8 ----------K--------------------G-T-C-------AAGS----------GSA

SmPap-12 ----------N--------------------K-T-C-----------------------F

SmPap-5 ----------V--------------------G-S-C-------NANK-------------

SmPap-6 ----------A--------------------G-S-C-------NAN--------------

OsPap-13 ----------K--------------------E-S-C-------KKQL-------------

HvPap-25 ----------R--------------------G-A-C-------SNSV-------------

OsPap-27 ----------R--------------------G-P-C-------NRAK-------------

OsPap-39 ----------R--------------------GPR---------NRAK-------------

HvPap-15 ----------R--------------------G-R-C-------LRNR-------------

HvPap-13 ----------D--------------------D-T-C-------DTTK-------------

OsPap-26 ----------A--------------------SAA-C-------DRAK-------------

OsPap-7 ----------Q--------------------G-A-C-------RAGG------------F

OsPap-2 ----------K--------------------G-R-C-------RVDD------------M

OsPap-10 ----------R--------------------G-K-C-------RADD-------------

OsPap-5 ----------Q--------------------G-K-C-------RVDD-------------

OsPap-6 ----------Q--------------------G-K-C-------RVDD-------------

HvPap-28 ----------Q--------------------G-----------HLRR-------------

OsPap-1 ----------R--------------------G-G-C-------DVGK------------L

OsPap-3 ----------R--------------------G-G-C-------DVGK------------L

OsPap-4 ----------Q--------------------G-S-C-------DVGK------------L

HvPap-18 ----------Q--------------------G-K-C-------DVDK-------------

HvPap-27 ----------Q--------------------G-T-C-------NVDK------------L

HvPap-29 ----------K--------------------G-T-C-------DVDK-------------

OsPap-21 ----------L--------------------G-T-C-------RASG-------------

OsPap-23 ----------Q--------------------G-A-C-----QFDASS------------S

AtPap-31 ----------K--------------------E-S-CR-----ANARR-------------

AtPap-21 ----------Q----------------------T-C-------SSST-----------TL

AtPap-22 ----------Q----------------------QTC-------ESNH-------------

AtPap-19 ----------E--------------------G-P-C-------RSNA-------------

AtPap-18 ----------D--------------------G-G-C-------RSNA-------------

AtPap-20 ----------E--------------------G-T-C---------RY-------------

OlPap-1 ----------K----------------------LSC-------NKFK-------------

OtPap-1 ----------A--------------------L-A-C-------NRWK------------L

SmPap-4 ----------E----------------------SHC-------NMKK-------------

AtPap-17 ----------E--------------------G-T-C-------DQEK-------------

CrPap-2 ---------AN--------------------GLG---------GGLQ-------------

VcPap-2 ----------G-------------------LG-FWC-------NKRK------------Q

AtPap-7 ----------N--------------------G-V-C-------DGRL-------------

AtPap-8 ----------N--------------------G-V-C-------EGRL-------------

AtPap-6 ----------D--------------------T-AAC-------KAIE-------------

AtPap-9 ----------Q--------------------G-S-C-------NRKQ-------------

CrPap-1 ----------D--------------------G-Q-C-------DVAK------------M

VcPap-1 ----------D--------------------G-V-C-------VAAK-------------

SmPap-2 ----------R--------------------G-A-C-------DKDK-------------

PtPap-30 ----------D--------------------G-T-C-------NTAK------------E

PtPap-21 ----------D--------------------G-T-C-------NTAK-------------

PtPap-23 ----------D--------------------G-T-C-------NTTK-------------

AtPap-16 ----------D----------------------ATC-------NSKK-------------

HvPap-17 ----------D--------------------G-S-C-------RRSA-------------

OsPap-12 ----------D--------------------G-A-C-------RAAA-------------

PtPap-5 ----------D--------------------G-T-C-------KSKK-------------

PtPap-12 ----------D--------------------G-T-C-------SSEK-------------

PtPap-6 ----------D--------------------G-T-C-------SSEK-------------

PtPap-9 ----------D--------------------G-T-C-------SSEK-------------

OsPap-16 ----------D--------------------D-K-C-------ATAG-------------

PtPap-18 ----------D--------------------G-T-C-------NSKK-------------

PtPap-15 ----------D--------------------G-T-C-------NSKK-------------

PtPap-22 ----------D--------------------G-T-C-------NSKK-------------

PtPap-11 ----------D--------------------G-A-C-------NSGK-------------

PtPap-26 ----------D--------------------G-T-C-------NTKK-------------

PtPap-27 ----------D--------------------G-T-C-------NTNK-------------

PtPap-28 ----------D--------------------G-T-C-------NTQK-------------

PtPap-36 ----------D--------------------G-T-C-------NTQK-------------

OsPap-25 ----------D--------------------G-R-C-------KTTA-------------

OsPap-15 ----------D--------------------G-K-C-------NGGS-------------

OsPap-14 ----------D---------------------DK-C-------KSVS-------------

OsPap-32 ----------D--------------------D-----------KFKS-------------

HvPap-8 ----------D--------------------G-T-C-------NKNK-------------

OsPap-29 ----------D--------------------G-T-C-------NKNK-------------

AtPap-10 ----------D--------------------G-T-C-------KKDK-------------

PtPap-34 ----------D--------------------G-T-C-------NKDR-------------

OsPap-31 ----------E--------------------G-Y-C-------REKQ------------P

SmPap-3 -------------------------------G-TRC-------DRSK-------------

PpPap-1 ----------D--------------------G-Q-C-------DEAR-------------

PpPap-2 ----------D--------------------G-R-C-------DEGR-------------

PtPap-17 ----------D--------------------D-T-C-------DRDK-------------

PtPap-19 ----------D--------------------D-K-C-------DKDK-------------

SmPap-1 ----------S--------------------G-S-C-------DESR-------------

AtPap-5 ----------D-------------------LG-L-C-------NADK-------------

PpPap-4 ----------D--------------------G-R-C-------DVNK-------------

PpPap-3 ----------D--------------------G-R-C-------DNNK-------------

PpPap-5 ----------D--------------------G-R-C-------DNSK-------------

AtPap-3 ----------G--------------------G-K-C-------NSFL-------------

OsPap-24 ----------D--------------------E-R-C-------DVNR--VSFVFFAPLVF

AtPap-4 ----------D-------------------VN-V-C-------NSDK-------------

OsPap-19 ----------D--------------------G-K-C-------NLAK-------------

HvPap-7 ----------D--------------------G-R-C-------DVLR-------------

OsPap-18 ----------D--------------------G-K-C-------DINR-------------

PtPap-25 ----------D--------------------S-M-C-------DPNR-------------

PtPap-31 ----------D--------------------S-M-C-------DPNR-------------

PtPap-29 ----------D--------------------G-R-C-------DTYR-------------

AtPap-1 ----------D--------------------G-T-C-------DQIR-------------

AtPap-2 ----------D--------------------G-R-C-------DQNR-------------

HvPap-6 ----------D--------------------N-R-C-------DANK-------------

OsPap-9 ----------N--------------------G-T-C-------DAVR-------------

HvPap-30 ----------N--------------------G-T-C-------KAAR-----------VA

HvPap-10 ----------R--------------------G-T-C-------NVAR-----------AA

HvPap-11 ----------R--------------------G-T-C-------NVAR-----------AA

OsPap-11 ----------E--------------------G-T-CRRGSTEGDDDG-------------

OsPap-20 ----------E--------------------G-D-C-------DEGK-------------

HvPap-4 ----------E--------------------G-S-C------GDGKK-------------

HvPap-5 ----------E--------------------G-S-C------GDGKK-------------

AtPap-15 ----------E--------------------G-I-C-------QEQK-------------

PtPap-33 ----------E--------------------G-T-C-------DEKR-------------

AtPap-14 ----------E--------------------G-T-C-------EMQK-------------

PtPap-32 ----------E--------------------G-T-C-------EMRK-------------

PtPap-35 ----------E--------------------G-T-C-------EMRK-------------

HvPap-14 ----------Q--------------------A-SSC-------NKKP-------------

OsPap-30 ----------Q--------------------S-S-C-------KSSA-------------

AtPap-12 ----------D-------------------VQ-F-C-------RANS------------I

PtPap-7 ----------E----------------------EPC-------DSNK-------------

AtPap-11 ----------D--------------------G-K-C-------DASK-------------

HvPap-9 ----------Q--------------------G-S-C-------DQAK-------------

OsPap-28 ----------Q--------------------G-R-C-------NKAK-------------

AtPap-13 ----------D--------------------E-T-C-------DTNK-------------

PtPap-8 ----------D--------------------G-H-C-------DAAK-------------

PtPap-10 ----------D--------------------G-H-C-------DANK-------------

VcPap-14 ---RPGSCRAGRQVTDCRP---------HPGRWEVREVLWGSAAAANVSAAKELVMSAGS

VcPap-7 ---PPERRPGGV-FKYIYI-----------------TD--LTAAKQHIRTYGSVTTFFAV

CrPap-7 ---TPVLYAGGA-FTRTTI-----------------YD--LTAAKAHIRTYGSLLTYFAV

VcPap-8 PESRPAGRFQ---YISI-------------------TN--LDTAKDHIRLYGAVLTYFAV

VcPap-13 ---PPLRYAGGMFWKTT-----------F-------SN--LTLAKQHIRMYGSVTSYFAV

VcPap-11 ---PPERRAEGG-FKDT-A---------Y-------TD--ITQAKQHIRMYGAVTSYFAV

VcPap-9 ---APERRAGGI-FKKAYI-----------------TD--LTVAKNHIRTSGAVITYFAV

VcPap-10 ---PPEQRAGGNFSRFV-----------Y-------LD--ISMAKDHIRTSGAVMTYFAV

OlPap-8 NLSSQRYEVTS--LVTCGT-----------------ND--FNCMALELFYHGPVSSYVGD

OtPap-9 QTANTTRYAVG--HLVQCG---------L-------ND--YDCMASEIFERGPVTTFVGD

OlPap-9 ---DHPCMVAGTTPQSC-P---------A-----------KCADGSALSFVHPTSEPYTC

OtPap-8 -EACPATCADGSKFQLVYP-KNLPYTCPP-------DD--IACIAKEIKNRGSVAVTFGP

VcPap-12 AAFAAAVEFQV--LRAVRI---------D-------MD--NQSLKAAIAAGMPVMAGFTC

CrPap-9 VTAFPRLTVSE--YGRV-------------------RG--RLQMKAEIYARGPISCGI-A

CrPap-6 IYDYKRLTVSE--HGRV-------------------RG--RYQMKAEIYKRGPISCGIDA

VcPap-4 VYEYKRLTVSE--YGRL-------------------KG--AHQMKAEIFRRGPISCGVDA

OlPap-7 LSTFPNATVAE--YGMI-------------------SG--EKEIMAEIFARGPVSAGIDA

OtPap-7 LSTFPNATVAE--YGTI-------------------RG--EKAIMAEIYARGPVAAGIDA

CrPap-10 -ECNNCMPLDG--VETCWPIHRPIRYYLTAWGQ---VDKGVEAMMSEIYHRGPITCGQVC

VcPap-6 -QCLNCMPING--VDTCWPIERPVKYYLNAWGNLD-KS--VEAMMSEIYHRGPITCGIAC

CrPap-8 RYSPTGLRIEA--WETI-P-----------------AS--EFALIQALANQ-PVVALLHA

CrPap-11 TYSPTGLRIQA--WETI-P-----------------PS--EFALIQALANQ-PVVALLHA

SmPap-19 WRDSKHFTVQT--YAVN-------------------SD--IYDIQAEIYKNGPVEVSYTV

PpPap-10 WVQSKHLGVNA--YEMS-------------------ME--PEDLMAELYTNGPVEVAFEV

PpPap-9 WVKSKHLSVNA--YEVS-------------------KE--PEDLMAELYTNGPIEVSFEV

PpPap-11 WVSSKHLGVSA--YEVS-------------------ME--PEELMAELFTNGPIEVAFDV

AtPap-32 WGESKHYGVGA--YRIN-------------------PD--PQDIMAEVYKNGPVEVAFTV

AtPap-29 WRESKHYGVSA--YKVR-------------------SH--PDDIMAEVYKNGPVEVAFTV

AtPap-30 WSESKHYSVST--YTVK-------------------SN--PQDIMAEVYKNGPVEVSFTV

PtPap-1 WKKSKHYGVKP--YRID-------------------SD--PESIMAEIYKNGPVEVAFTV

PtPap-2 WAESKHFSVNA--YRID-------------------SD--PHSIMAEVSSNGPVEVAFTV

PtPap-3 WAESKHFSVNA--YRID-------------------SD--PHSIMAEVSMNGPVEVAFTV

HvPap-20 WKKNKHFSVNA--YRVH-------------------SN--PHDIMAEVYKNGPVEVAFTV

HvPap-19 WQEKKHFSIDA--YQVN-------------------SD--PHDIMAEVYKNGPVEVAFTV

OsPap-45 WLEKKHFSVNA--YRVN-------------------SD--PHDIMAEVYQNGPVEVAFTV

OlPap-5 ---KKVYDTPA--MCDLAQ---------V-------AG--EEPLYRAIFERGPVAVGINA

OtPap-5 EHKYNTPAMCD--LEQV-------------------LG--EEPLYRAIYERGPVAVGINA

OlPap-6 PDWPRVAQVHG--YCELSL---------S-------GG--EKELMHALSKS-PVAIGVNA

OtPap-6 PQIPRKVQVLG--YCELSL---------S-------GG--EKELMYALMKS-PVAIGVNA

CrPap-5 ---RDGIRLRG--YVGL-----------V-------TT--ESAMFKALCSGVPALTVIVN

OlPap-4 -DQSRGVKIDG--YCEIPP---------L-------EG--ESTLINLMRALQQQTVAVGV

OtPap-4 EPSTRGVKIDG--YCEIPP---------L-------QG--VPTVLNIMRALKQQTVAVGL

HvPap-31 --AAAVIKVNG--YTTV-------------------SG--ESAMASYVQTTGPLSVCLDA

OlPap-3 -ARKHAASIDD--YIVL-----------E-------NT--WDDLKSAIYMQ-PVSVAVNA

OtPap-3 -ARKHAASIDD--YIIL-----------D-------ND--WKDLKSAIYMQ-PVSVAVNA

SmPap-13 ---SSLVTISG--VERIYS---------S-------SD--DTELLNAVAKQ-PVAVIVDG

SmPap-9 -KQGK-VSLKE--FESIEG---------T-------DKAIERNMLKAIHNQ-PLTAVIDG

CrPap-3 ---TRVGLFSG--YMNV-E---------S-------RN--ELALMEAVAKYGPIAVSVNA

VcPap-3 ---SMVGQFSG--YWAV-E---------S-------RN--EEALMEAVWKYGPVAVSVDA

CrPap-4 AAAPLVARFSD--FVNV-P---------P-------RD--ESALMEAVYLHGPVAVLMNA

VcPap-5 ---PVSARFSG--YLNI-P---------S-------RD--EAALMEAVALHGPVAVLMNA

SmPap-17 -CHTNDVSMSQ--IGTV-P---------G-------TS--DSFLVEAVSKQ-PVIARIDG

HvPap-32 ----KGVGIRG--WARVEP-----------------RN--ENALMWSVFKH-PVTVGIDA

SmPap-7 ---G--VFIDG--FDTLRGSLCAPGVSAS-------DI--EEELKMRVAQQ-PVTALIDG

SmPap-11 ---GLVVTIDG--FDNLWG---------S-------SS--DAEMVERVAKQ-PVTALVDG

SmPap-15 TINRYSVGIDD--YDNLYG---------S-------SD--DTDMVAQVAKQ-PVTALIDG

SmPap-16 -SACVLLCIDN--YDYIGS---------S-------SD--DTEIVKQVAKQ-PVTALIDG

HvPap-12 ---NAAVQVLD--SVNI-T---------L-------NA--EDELKNAVGLVRPVSVAFQV

OsPap-40 ---NVGVKVLD--SVNI-T---------L-------GA--EDELKNAVGLVRPVSVAFQV

AtPap-28 ---NIGVQVRD--SVNI-T---------L-------GA--EDELKHAVGLVRPVSVAFEV

AtPap-27 ---NVGVQVLN--SVNI-T---------L-------GA--EDELKHAVGLVRPVSIAFEV

PtPap-4 ---NVGVRVVE--SVNI-T---------L-------GA--EDELKHAVAFVRPVSVAFEV

PpPap-8 ---TIGAQVWD--VVNI-T---------E-------GA--ETQLKHAIATMRPVSVAFEV

SmPap-10 ---SIGAKVYD--VVNI-T---------E-------GA--EDELIHAVAFNRPVSIAYEV

HvPap-22 -AARRIGKITG--VQDVQP---------G-------SN--EDALALAVLRT-PVTVQIDG

HvPap-24 DGLSRFASTER--FRVV-----------Y-------SN--ERALMAAVAVQ-PVTVDI-G

OsPap-34 -GKPPIVKVDA--VKPV-A---------N-------T---EAALLLKVFQQ-PISVGIDA

OsPap-43 -GKQPIITVDD--VKWV-----------N-------KS--EAALLLKVYQQ-PISVALDA

OsPap-42 -GR-QAVTLDC--IRQL-P---------L-------DN--EAALKERVYIQ-PVSVAVDA

OsPap-44 -PAVPVVKMDG--AVDV-----------T-------AS--EAALKRSVYRA-PVAVSIEA

OsPap-35 --GKPVVKMDG--AASV-P---------S-------SN--EVALKQSVYKQ-PVSVLIEA

OsPap-37 --GKPVVKTDG--TLQV-----------A-------SS--ETALKQSVYGQ-PVSVLIEA

OsPap-33 -NKAPIVKIDS--YSFV-D---------P-------ND--EEALKQAVYSQGPVSVLIEA

OsPap-36 -EKPPFVKIDG--ICFA-Q---------S-------GD--ETALKLAVLSQ-PVSVIIQI

OsPap-38 -RKPPFVKIDG--ECLV-P---------S-------GN--ETALKLAVLSQ-PVSVVITI

HvPap-16 ---PVAVRLRS--YKKVTP---------P-------GN--EAGLKEAVAQQ-PVAVSFDY

SmPap-14 --SKVVATIAN--YSRV-----------S-------ED--EDQIAANLVKNGPLSIALRG

SmPap-18 --SKVAATIAN--YSTV-----------S-------ED--EDQIAANLVKNGPLSIALNA

OlPap-2 ---KLGATLKN--FSFV-S---------D-------DE--KQMAAALVKYG-PLSIGINA

OtPap-2 ---TLGATLKN--FSYV-S---------S-------DE--KQMAAALVKHG-PLSIGINA

HvPap-3 ---KVAVRVAN--FTAV-P---------L-------DE--DQMRAALVRGG-PLAVGLNA

OsPap-22 ---RVAVRVAN--FTVV-A---------PPGGNDGDGD--AQMRAALVRHG-PLAVGLNA

AtPap-26 ---KVAVRVLN--FTTI-P---------L-------DE--NQIAANLVRHG-PLAGDIYA

PtPap-24 ---KIAVKVAN--FTSI-A---------V-------DE--NQIAANLVHHG-PLAIGLNA

PpPap-6 ---KVAVKVSN--FTNI-P---------V-------DE--DQVAAYLIKSG-PLAIGINA

PpPap-7 ---KVAAKVSN--FTNI-P---------I-------DE--DQVAAYLIKSG-PLAIGINA

SmPap-8 ---KIVASVAN--FSTV-S---------L-------DE--DQIAANLVKHG-PLAIGINA

HvPap-2 --SKIAAQVKN--FSTV-----------A-------ID--EDQIAANLVKHGPLAIGINA

OsPap-17 ---KIVAQVKN--FSVI-S---------V-------NE--DQIAANLVKHG-PLAIAINA

HvPap-1 ---KIAASVQN--YSVV-A---------V-------DE--EQIAANLVKYG-PLAIGINA

OsPap-41 ---KIVTSVQN--FSVV-S---------V-------DE--DQIAANLVKHG-PLAIGINA

AtPap-25 ---KIVASVSN--FSVV-S---------S-------DE--DQIAANLVQHG-PLAIAINA

AtPap-23 ---KIVASVSN--FSVI-S---------I-------DE--EQIAANLVKNG-PLAVAINA

AtPap-24 ---KIVASVSN--FSVV-S---------I-------NE--DQIAANLIKNG-PLAVAINA

PtPap-13 ---KVAAGVAN--FSAV-S---------L-------DE--DQIAANLVKNG-PLAVAINA

PtPap-14 ---KVAARVAN--FSVV-S---------L-------DE--DQIAANLVKNG-PLAVAINA

PtPap-16 ---KVVASVSN--FSVV-S---------I-------DE--DQIAANLVKHG-PLSVAINA

PtPap-20 ---KVAASVSN--FSVV-S---------L-------DE--DQIAANLVKHG-PLSVAINA

HvPap-21 ---LVAVSLRG--YRKL-P---------Y-------NEQSEQKLLEAVAQQ-PVAVAVDA

OsPap-8 SSSSVAVVIRG--VQKVTP-----------------HD--KDALRAAVERQ-PVAADMDS

SmPap-12 -FLERDFTIDG--YEHV-L---------P-------NN--EEALKKAVAHQ-PVSVMIDA

SmPap-5 -AKNKVAEITG--FKVV-T---------E-------DS--ADALMKAVSKT-PVTVSICG

SmPap-6 --KNKVVEITG--YKDV-T---------K-------DS--ADALMKAVSKT-PVTVGICG

OsPap-13 -LGVHNATVRG--VVTL-P---------E-------NR--EDLIMAAVARQ-PVAVVFDA

HvPap-25 ---RNVVKILG--GGVI-P---------P-------RN--EAEMQVAVAGQ-PIGVAIEV

OsPap-27 -SAHHAAKITG--FGKV-P---------P-------RN--EAALQAAVARQ-PVAVAIEV

OsPap-39 -SAHHAAKITG--FGKVTP-----------------RN--EAALQAAVARQ-PVAVAIEV

HvPap-15 ---KPAAKIVG--FRKV-K---------S-------NS--EVSLMNAVANQ-PVAVSISS

HvPap-13 -LSHHAASISG--FQRV-A---------T-------RS--ELSLTNAVAMQ-PVAVSIEA

OsPap-26 -LGHHAATIAG--LRRV-A---------T-------RS--EASLANAAAAQ-PVAVSIEA

OsPap-7 AAPNSAAAVGG--ARWA-R---------L------YGD--EGALQALAAGQ-PVVVVVEA

OsPap-2 -LFNHAARVGG--YRAV-P---------P-------AD--ERQLATAVARQ-PVTAYVDA

OsPap-10 ALFNHAARIGG--HRAV-P---------P-------GD--ERQLATAVARQ-PVTAYIDA

OsPap-5 MLFNHAARIGG--YRAV-P---------P-------ND--ERQLATAVARQ-PVTVYIDA

OsPap-6 MLFNHAASIGG--YRAV-P---------P-------ND--ERQLATAVARQ-PVTVYIDA

HvPap-28 --XQACCSTTRRPSRDSRP---------C-------RPTTSGSLALAVARQ-PVTVYIDA

OsPap-1 -LSGHSASLSG--FRAV-P---------P-------ND--ERQLALAVARQ-PVTAYIDA

OsPap-3 -LSDHSASVSG--FAAV-P---------P-------ND--ERQLALAVARQ-PVTVYIDA

OsPap-4 -LFDHSASVSG--FAAV-P---------P-------ND--ERQLALAVARQ-PVTVYIDA

HvPap-18 LMFDHQASIKG--FKAV-P---------S-------NN--EAQLAIAVAMQ-PVTVYIDA

HvPap-27 -LFDNQASVKG--FKAV-P---------P-------ND--EHQLALAVARQ-PVTVYIDA

HvPap-29 LLFDHQASVKG--FKAV-P---------P-------NN--ERQLALAVARQ-PVTVYIDA

OsPap-21 --KPVAASIRG--FQYV-P---------P-------NN--ETALLLAVAHQ-PVSVALDG

OsPap-23 -ASGVAATISG--YQRV-N---------P-------ND--EGSLAAAVASQ-PVSVAIEG

AtPap-31 ---APHTQIRG--FQMV-P---------S-------HN--ERALLEAVRRQ-PVSVLIDA

AtPap-21 SSSFRAATISG--YETV-P---------M-------NN--EEALLQAVSQQ-PVSVGIEG

AtPap-22 ---LAAATISG--YETV-P---------Q-------ND--EEALLKAVSQQ-PVSVAIEG

AtPap-19 ---RPAILIRG--FENV-P---------S-------NN--ERALLEAVSRQ-PVAVAIDA

AtPap-18 ---RPAARISG--FQTV-P---------S-------NN--ERALLEAVSRQ-PVSVSMDA

AtPap-20 -NGKPSAWIRG--FQTV-P---------S-------NN--ERALLEAVSKQ-PVSVSIDA

OlPap-1 -LQLHVATIDG--FEDV-P---------P-------GD--EKELEKAVSQQ-PVSIAIEA

OtPap-1 -Q-LHVATIDG--FKDV-P---------P-------GD--EKELEKAVSQQ-PVSIAIEA

SmPap-4 -LNSRVVAIDG--YEAI-P---------D-------GD--EQALLRAVAKQ-PVSVAIEG

AtPap-17 -SKNKVVTIQG--YQKV-----------A-------QN--EASLQIAAAQQ-PVSVGIDA

CrPap-2 -LLQPAVSIDG--YEDV-P-----------------TS--EPALLKAVAGQ-PVAVAICA

VcPap-2 -TDRPAVSIDG--YEDV-P-----------------QG--EDNLLKAVAHQ-PVAVAICA

AtPap-7 KENNKNVMIDG--YENL-P---------A-------ND--ESALMKAVAHQ-PVTAVIDS

AtPap-8 KEDNKNVMIDG--YENL-P---------A-------ND--EAALMKAVAHQ-PVTAVVDS

AtPap-6 MKTTRVVTING--HEVV-P---------V-------ND--EMSLKKAVAYQ-PISVMISA

AtPap-9 STSNKVITIDS--YEDV-P---------A-------ND--EISLQKAVAHQ-PVSVGVDK

CrPap-1 -K-RRVVTIDS--YEDV-P---------E-------ND--EVALKKAAAHQ-PVAVAIEA

VcPap-1 -KNRRVVTIDG--YVDI-P---------E-------ND--EVALKKAAAHQ-PIAVAIEA

SmPap-2 -VKYHAATING--FQAI-P---------P-------QS--EELLLRAVANQ-PVSVAIEA

PtPap-30 -E-KKVVSIEG--YVDV-D---------P-------SD---SALLCATVQQ-PISVGMDG

PtPap-21 -EEIKVVSIDG--YKDV-----------D-------ET--DSALLCAAAQQ-PISVGIDG

PtPap-23 -EEIKVVSIDG--YTDV-----------D-------ET--DSALLCATVQQ-PISVGMDG

AtPap-16 -TNPKATSITG--YEDV-P---------V-------ND--EQALMKAVAHQ-PVSVGIEG

HvPap-17 ----SAASIRG--YEDV-P---------A-------NN--EAALMAAVAHQ-PVSVAING

OsPap-12 --GRAAASIRG--FQDV-P---------S-------ND--EGALMAAVARQ-PVSVAING

PtPap-5 -TASIEAKITG--YEDV-P---------V-------NN--ENALLQAVAKQ-PVSVAVEG

PtPap-12 -AASIAAEITG--DENA-P---------K-------NN--ENALLQAVAKQ-PVSVGVDG

PtPap-6 -AASTEAQITG--YEDV-P---------Q-------NN--ENALLQAVAKQ-PVSVAVDG

PtPap-9 -AASTEAQITG--YEDV-P---------Q-------NN--ENALLQAVAKQ-PVSVGVDG

OsPap-16 -AGAAAATIKG--YEDV-P---------A-------ND--EAALLKAVANQ-PVSVAIDG

PtPap-18 -EASRIAKITG--YESV-P---------A-------NS--EAALLKAVASQ-PISVSIDA

PtPap-15 -QASHIAKITG--YESV-P---------A-------NS--EAELLKVVANQ-PISVSIDA

PtPap-22 -QASHIAKITG--YESV-P---------A-------NS--EAELLKVVANQ-PISVSIDA

PtPap-11 ----AAAKITG--YEDV-P---------A-------NS--EAALLKAVANQ-PVSVAIDA

PtPap-26 -SAIHAAKITG--FEDV-P---------A-------NS--EAALMKAVAKQ-PVSVAIDA

PtPap-27 -AAIHAAKITG--FEDV-P---------A-------NS--EAALMKAVAKQ-PVSVAIDA

PtPap-28 -EATHAAKITG--FEDV-P---------A-------NS--EAALMKAVAKQ-PVSVAIDA

PtPap-36 -EVSHAAKITG--FQDV-P---------A-------NS--EAALMKAVAKQ-PVSVAIDA

OsPap-25 -AADVAASIRG--YEDV-P---------A-------ND--EPSLMKAVAGQ-PVSVAVDA

OsPap-15 ---NSAATIKG--YEDV-P---------A-------NN--EAALMKAVANQ-PVSVAVDG

OsPap-14 ---NSVASIKG--YEDV-P---------A-------NN--EAALMKAVANQ-PVSVAVDG

OsPap-32 -VSNSVASIKG--YEDV-P---------A-------NN--EAALMKAVANQ-PVSVAVDG

HvPap-8 -LKKHVVTIDG--YKEV-P---------S-------SK--EDLLLQAVAQQ-PISVGICG

OsPap-29 -LKRRVVTIDG--YKDV-P---------A-------NN--EDMLLQAVAQQ-PVSVGICG

AtPap-10 -LKQKVVTIDS--YAGV-K---------S-------ND--EKALMEAVAAQ-PVSVGICG

PtPap-34 -MKRRVVTIDK--YVDV-P---------E-------NN--EKQLLQAVAAQ-PVSVGICG

OsPap-31 -H-SKVITITG--YEDV-P---------A-------NS--ETSLLKALAHQ-PVSVGIAA

SmPap-3 -MNAKVVTIDG--YEDV-P---------V-------ND--ETALLKAVAHQ-PVSVAIDA

PpPap-1 KETSKVVVIDD--YQDV-P---------T-------KS--ESSLLKAVSKN-PVSVAIEA

PpPap-2 -RNSKVVVIDD--YQDV-P---------T-------QS--ESALMKALTKN-PVSVAIEA

PtPap-17 -MKTKAVSIDG--FEDVLP-----------------FD--EKALQKAVAHQ-PVSVAIEA

PtPap-19 -MKTKAVSIDG--FEDVLP-----------------YD--EKALQKAVAHQ-PVSVAIEA

SmPap-1 -RNSHVVTIDG--FEDV-P---------A-------ES--EADLLKAVANQ-PVSVAIEA

AtPap-5 NNNTRVVTIDG--YEDV-P---------R-------DD--EKSLKKAVAHQ-PVSVAIEA

PpPap-4 -MNARVVTIDS--YEDV-P---------E-------ND--EEALKKAVAGQ-PVSVAIEA

PpPap-3 -KNAHVVTIDG--YEDV-P---------E-------ND--EEALKKAVAGQ-PVSVAIEA

PpPap-5 -KNAHVVTIDG--YEDV-P---------E-------ND--EEALKKAVAGQ-PVSVAIEA

AtPap-3 -KNSRVVSIDG--YEDV-P---------T-------KD--ETALKKAISYQ-PVSVAIEA

OsPap-24 QKNAKVVTIDS--YEDV-T---------P-------NS--ETSLQKAVANQ-PVSVAIEA

AtPap-4 -KNTRVVTIDG--YEDV-P---------Q-------ND--EKSLKKALANQ-PISVAIEA

OsPap-19 -RSRKVVSIDG--FEDV-P---------E-------ND--ELSLQKAVAHQ-PVSVAIDA

HvPap-7 -KNAKVVSIDG--FEDV-P---------E-------ND--EKSLQKAVAHH-PVSVAIEA

OsPap-18 -ENAKVVSIDG--FEDV-P---------Q-------ND--EKSLQKAVAHQ-PVSVAIEA

PtPap-25 -KNARVVTIDG--YEDV-P---------Q-------ND--EKSLRKAVANQ-PVSVAIEA

PtPap-31 -KNARVVTIDG--YEDV-P---------Q-------ND--EKSLKKAVANQ-PVSVAIEA

PtPap-29 -KNAKVVSIDS--YEDV-P---------E-------ND--ETALKKAVANQ-PVSVAIEG

AtPap-1 -KNAKVVTIDS--YEDV-P---------T-------YS--EESLKKAVAHQ-PISIAIEA

AtPap-2 -KNAKVVTIDS--YEDV-P---------E-------NS--EASLKKALAHQ-PISVAIEA

HvPap-6 -KNAKVVTIDG--YEDV-P---------V-------NS--EKSLQKAVANQ-PISVAIEA

OsPap-9 ARRAPLVVIDG--HQNV-P---------A-------NS--EAALAKAVANQ-PVSVAIDA

HvPap-30 KSSPVVVHIDG--HQDV-P---------A-------NS--EEALAKAVANQ-PVSVALDA

HvPap-10 QNSPVVVHIDG--HQDV-P---------A-------NS--EEDLARAVANQ-PVSVAVEA

HvPap-11 QNSPVVVHIDG--HQDV-P---------A-------NS--EEDLARAVANQ-PVSVAVEA

OsPap-11 -EAAAAVTISG--YEDV-P---------R-------NN--EQALLKALAHQ-PVSVAIEA

OsPap-20 --GAAVVTISG--YEDV-P---------A-------ND--EQALVKALAHQ-PVSVAIEA

HvPap-4 -AESEAVTISG--YEDV-P---------A-------ND--EQALIKALAHQ-PVSVAIEA

HvPap-5 -SESEAVSISG--YEDV-P---------T-------KD--EQALIKALAHQ-PVSVAIEA

AtPap-15 -EDVERVTISG--YEDV-P---------E-------ND--DESLVKALAHQ-PVSVAIEA

PtPap-33 -EEMEVVTISG--YHDV-P---------R-------ND--EQSLLKALAHQ-PLSVAIDA

AtPap-14 -DESETVTING--HQDV-P---------T-------ND--EKSLLKALAHQ-PLSVAIDA

PtPap-32 -EESEVVTISG--YHDV-P---------Q-------NS--EESLLKALANQ-PLSVAIEA

PtPap-35 -AESEVVTISG--YHDV-P---------Q-------NS--EESLLKALANQ-PLSVAIDA

HvPap-14 ---SAVVTIDG--YEDV-P---------A-------ND--ETALKKAVAAQ-PVAVAIEA

OsPap-30 -ASSPAVTIDG--YEDV-P---------A-------NS--ESALKKAVANQ-PVSVAIEA

AtPap-12 -G-GETVTIDG--HEHV-P---------E-------ND--EEELLKAVAHQ-PVSVAIDA

PtPap-7 -MNSPVVNIDG--YEMV-P---------E-------ND--ENALMKAVANQ-PVAIAMDA

AtPap-11 -DNGVLVTIDG--HEDV-P---------E-------ND--ENALLKAVANQ-PVSVAIDA

HvPap-9 -ENAQAVTIDG--YEDV-P---------A-------ND--ESALQKAVAGQ-PVSVAIDA

OsPap-28 -ASSHDVTIDG--YEDV-P---------A-------ND--ESALQKAVANQ-PVAVAVEA

AtPap-13 -ENAPVVSIDG--HEDV-P---------K-------NS--EDDLMKAVANQ-PVSVAIDA

PtPap-8 -ENNPAVSIDG--YEKV-P---------E-------ND--EDALLKAAANQ-PVSVAIDA

PtPap-10 -ANQPAVSIDG--HEDV-L---------H-------NN--ENALLKAVANQ-PVSVAIDA

VcPap-14 AVACFREF--SDTKRIGRE--------------------IYRDN--PAAVS---------

VcPap-7 HADLYTWS--PASGASRVYVLSAALTSFVAHALTFLNPYVWD-GVSP-QTG---------

CrPap-7 YNDFFNWN--KASA-------------------------PYK-W--DGVSS---------

VcPap-8 YNDFYNWR--KGSA-------------------------PYVWDGKSALSG---------

VcPap-13 YTDFLSWT--ASSP-------------------------PYIWNRASLFLG---------

VcPap-11 YGDFFRWR--ASSP-------------------------PYAWDGISALAG---------

VcPap-9 YNDFFYWP--VSSK-------------------------PYV-W--DGVSP---------

VcPap-10 YNDFFYWSATLTPY-------------------------VWD-GVSP-LAG---------

OlPap-8 VFDEFYKY--KS-G-------------------------VYS-L--SKDVA---------

OtPap-9 VYDEFYQY--ER-G-------------------------VYKLSKDPAARG---------

OlPap-9 PKGDVTHT--GS-G-------------------------VYT-V--PNDAG---------

OtPap-8 VHEDFYGH--KE-G-------------------------VYK-V--TESSG---------

VcPap-12 YENLYDDDVQST-G-------------------------VIK-MPKGDRIG---------

CrPap-9 SSKGVQAY--KG-G-------------------------VYA----EYRER---------

CrPap-6 TD-QLETY--TG-G-------------------------VYA----ERKSN---------

VcPap-4 TD-NMDSY--TG-G-------------------------VYA----EYKEK---------

OlPap-7 DG--LRGY--VG-G-------------------------IYKDT--P-------------

OtPap-7 DG--LRGY--VG-G-------------------------IYK-D-TP--SF---------

CrPap-10 PEDFTWHY--NG-G-------------------------IYK----DTSGD---------

VcPap-6 PDDFTWHY--KG-G-------------------------IYK----DTSGD---------

CrPap-8 SEDWFDYWG-RG-R-------------------------VYD-G--N--CS---------

CrPap-11 GEDWSLYFGQRG-R-------------------------VYD-G--N--CS---------

SmPap-19 YE-DFAHY--KS-G-------------------------VYK-HVFGEVLG---------

PpPap-10 YE-DFAHY--KT-G-------------------------VYK-HLFGGFMG---------

PpPap-9 FE-DFAHY--KT-G-------------------------VYK-HVYGRYIG---------

PpPap-11 FE-DFAHY--KT-G-------------------------VYK-HLYGGYIG---------

AtPap-32 YE-DFAHY--KS-G-------------------------VYK-YITGTKIG---------

AtPap-29 YE-DFAHY--KS-G-------------------------VYK-HITGTNIG---------

AtPap-30 YE-DFAHY--KS-G-------------------------VYK-HITGSNIG---------

PtPap-1 YE-DFAHY--KS-G-------------------------VYK-HITGGMMG---------

PtPap-2 YE-DFAHY--KS-G-------------------------VYK-HITGDAMG---------

PtPap-3 YE-DFAHY--KS-G-------------------------VYK-HITGDVMG---------

HvPap-20 YE-DFAHY--KS-G-------------------------VYK-HITGGVMG---------

HvPap-19 YE-DFAHY--KS-G-------------------------VYK-HITGGVMG---------

OsPap-45 YE-DFAHY--KS-G-------------------------VYK-HITGGMMG---------

OlPap-5 N--KLQAY--GS-G-------------------------VIM----LDDCK---------

OtPap-5 N--RLQAY--DD-G-------------------------VIM----MDDCH---------

OlPap-6 NK-KFQLY--DR-G-------------------------ILR----LKDCP---------

OtPap-6 NS-KFQLY--DS-G-------------------------ILR----MKDCP---------

CrPap-5 ACPVFARY--SG-G-------------------------VFNTD-----CP---------

OlPap-4 NIKPLQLY--GG-G-------------------------IVR----LHDCP---------

OtPap-4 NIKPLQLY--GG-G-------------------------IVR----TRDCP---------

HvPap-31 S--QWNSY--TG-G-------------------------IVS----T--CG---------

OlPap-3 MGEHFRFY--SG-G-------------------------ILT----YDQCQ---------

OtPap-3 LGAPFRFY--SG-G-------------------------ILT----YDDCQ---------

SmPap-13 TTPQFINY--RS-G-------------------------IYT-G--PFADA---------

SmPap-9 SKAEFKSY--AG-G-------------------------IYK-G--P--CA---------

CrPap-3 DPEAFSFY--SE-G-------------------------VFD-E--P-ACT---------

VcPap-3 APESFRFY--SG-G-------------------------VYD-E--P-TCS---------

CrPap-4 ARVPFKFY--SE-G-------------------------VYH----NEECG---------

VcPap-5 ALAPFRFY--SE-G-------------------------VYY-N--E-ECG---------

SmPap-17 SASEFDSY--AG-G-------------------------IYS-G--P--CS---------

HvPap-32 NSPAFLHY--RG-G-------------------------LFD-G--P--CN---------

SmPap-7 SSKDFAKY--TG-G-------------------------IFK-G--P--CH---------

SmPap-11 DADAFKKY--KS-G-------------------------IFK-G--P--CS---------

SmPap-15 NAPEFLRY--KG-G-------------------------IYK-G--P--CK---------

SmPap-16 NAPEFHRY--KG-G-------------------------IYK-G--P--CK---------

HvPap-12 ID-GFRQY--KS-G-------------------------VYT----SDHCG---------

OsPap-40 IN-GFRMY--KS-G-------------------------VYT----SDHCG---------

AtPap-28 VH-EFRFY--KK-G-------------------------VFT----SNTCG---------

AtPap-27 IH-SFRLY--KS-G-------------------------VYT----DSHCG---------

PtPap-4 VG-SFRLY--KE-G-------------------------VYT----TSTCG---------

PpPap-8 VH-DFRLY--NG-G-------------------------VYT----SLNCH---------

SmPap-10 LK-DFRFY--KS-G-------------------------VYT----SNVCG---------

HvPap-22 SGSVLQNY--KS-G-------------------------VYK-G--P--CT---------

HvPap-24 VDMYFHYYSEDM-G-------------------------VYT-G--P--CN---------

OsPap-34 SA-DLQHY--KK-G-------------------------VFT-G--R--CK---------

OsPap-43 SG--WQFY--QG-G-------------------------VFT-G--P--CQ---------

OsPap-42 KN----------------------------------------------------------

OsPap-44 TQ-SLQLY--KE---------------------------VIN------------------

OsPap-35 NS-NFQLY--KQ-G-------------------------VYS-G--P--CG---------

OsPap-37 DT-NFQLY--KS-G-------------------------VYS-G--P--CG---------

OsPap-33 SY-EFMIY--QG-G-------------------------VFS-G--P--CG---------

OsPap-36 SD-RFHSY--HG-G-------------------------VFD-G--P--CG---------

OsPap-38 SD-EFRSY--RG-G-------------------------VFR-G--P--CG---------

HvPap-16 SDPCFQHY--IG-G-------------------------VYNAG-----CSRSGVYIKGA

SmPap-14 N--VLFTY--EG-G-------------------------VAC----PRICP---------

SmPap-18 N--YIMDY--MG-G-------------------------VAC----PRICP---------

OlPap-2 A--WMQSY--IG-G-------------------------VAC----PWLCD---------

OtPap-2 A--WMQTY--IG-G-------------------------VAC----PWLCD---------

HvPap-3 A--FMQTY--VG-G-------------------------VSC----PLICP---------

OsPap-22 A--YMQTY--VG-G-------------------------VSC----PLVCP---------

AtPap-26 V--FMQTY--IG-G-------------------------VSC----PLICS---------

PtPap-24 I--FMQTY--IG-G-------------------------VSC----PLICG---------

PpPap-6 E--FMQTY--IA-G-------------------------VSC----PIFCN---------

PpPap-7 E--FMQTY--VA-G-------------------------VSC----PIFCN---------

SmPap-8 V--FMQTY--IG-G-------------------------VSC----PIICS---------

HvPap-2 V--FMQTY--IG-G-------------------------VSC----PYICG---------

OsPap-17 A--YMQTY--IG-G-------------------------VSC----PFICG---------

HvPap-1 A--YMQTY--IG-G-------------------------VSC----PYICG---------

OsPap-41 A--YMQTY--IG-G-------------------------VSC----PYICG---------

AtPap-25 M--WMQTY--IG-G-------------------------VSC----PYVCS---------

AtPap-23 G--YMQTY--IG-G-------------------------VSC----PYICT---------

AtPap-24 A--YMQTY--IG-G-------------------------VSC----PYICS---------

PtPap-13 V--FMQTY--IG-G-------------------------VSC----PYICS---------

PtPap-14 V--FMQTY--IG-G-------------------------VSC----PYICS---------

PtPap-16 A--FMQTY--VG-G-------------------------VSC----PYICS---------

PtPap-20 V--FMQTY--IG-G-------------------------VSC----PYICS---------

HvPap-21 SSFEFQTY--KD-G-------------------------VFS-G--P--CG---------

OsPap-8 SDPEFRGF--KG-GR------------------------VYR-G--SAGCG---------

SmPap-12 GCPAFKFY--KS-G-------------------------ILT-S--S--CG---------

SmPap-5 SDENFQNY--KS-G-------------------------ILS-G--K--CD---------

SmPap-6 SDQNFQNY--RS-G-------------------------ILS-G--H--CS---------

OsPap-13 GDPLFQNY--RGNG-------------------------VYK-G--GTGCS---------

HvPap-25 GG-GMQFY--RS-G-------------------------VYS-G--P--CG---------

OsPap-27 GS-GMQFY--KG-G-------------------------VYT-G--P--CG---------

OsPap-39 GS-GMQFY--KG-G-------------------------VYT-G--P--CG---------

HvPap-15 HSSHFHHY--KG-G-------------------------IYN-G--P--CST--------

HvPap-13 GGANFQHY--RN-G-------------------------VYN-G--P--CG---------

OsPap-26 GGDNFQHY--RK-G-------------------------VYD-G--P--CG---------

OsPap-7 SEPDFRHY--RS-G-------------------------VYA-G--SAACG---------

OsPap-2 SGPAFQFY--GS-G-------------------------VFP-G--P--RGT------AA

OsPap-10 SGPAFQFY--GS-G-------------------------VFP-G--P--CGSGSGAAAAA

OsPap-5 SGPAFQFY--KS-G-------------------------VFP-G--P--CG---------

OsPap-6 SGPAFQFY--KS-G-------------------------VFP-G--P--CG---------

HvPap-28 STWEFQFY--SG-G-------------------------IFR-G--P--CS---------

OsPap-1 GAREFMFY--KG-G-------------------------VYR-G--P--CS---------

OsPap-3 SAPEFQFY--KG-G-------------------------VYR-G--P--CD---------

OsPap-4 SAQEFQFY--KG-G-------------------------VYK-G--P--CN---------

HvPap-18 SGSAFQFY--SG-G-------------------------IYR-G--P--CS---------

HvPap-27 SAWEFQFY--TG-G-------------------------IYR-G--P--CS---------

HvPap-29 STWEFQFY--SG-G-------------------------IFR-G--P--CS---------

OsPap-21 VGKVSQFF--SS-G-------------------------VFG-AMQNETCT---------

OsPap-23 SGAMFRHY--GS-G-------------------------VFT-A--D-SCG---------

AtPap-31 RADSFGHY--KG-G-------------------------VYA-G--L-DCG---------

AtPap-21 TGAAFRHY--SG-G-------------------------VFN-G--E--CG---------

AtPap-22 SGYEFIHY--SG-G-------------------------IFN-G--E--CG---------

AtPap-19 SEAGFVHY--SG-G-------------------------VYN----ARNCG---------

AtPap-18 TGDGFMHY--SG-G-------------------------VYD-G--P--CG---------

AtPap-20 DGPGFMHY--SG-G-------------------------VYD-E--P-YCG---------

OlPap-1 DTKAFMLY--QG-G-------------------------VFD----SKECG---------

OtPap-1 DTKSFQLY--DG-G-------------------------VYD-S--K-ECG---------

SmPap-4 ASKDFQHY--AS-G-------------------------VFT-G--H--CG---------

AtPap-17 GGFIFQLY--SS-G-------------------------VFTNY-----CG---------

CrPap-2 SA-NMQFY--SS-G-------------------------VIN----S--CC---------

VcPap-2 GA-SMQFY--SR-G-------------------------VIS-T-----CC---------

AtPap-7 SSREFQLY--ES-G-------------------------VFD-G--S--CG---------

AtPap-8 SSREFQLY--ES-G-------------------------VFD-G--T--CG---------

AtPap-6 A--NMSDY--KS-G-------------------------VYK-G--A--CS---------

AtPap-9 KSQEFMLY--RS-C-------------------------IYN-G--P--CG---------

CrPap-1 DAKSFQLY--GG-G-------------------------VYD-D--P-TCG---------

VcPap-1 DAKSFQLY--GG-G-------------------------VYD-D--P-TCG---------

SmPap-2 GGQDFQLY--SS-G-------------------------VFT-G--E--CG---------

PtPap-30 SALDFQLY--TG-G-------------------------IYD-G--D--CSG------DP

PtPap-21 SAIDFQLY--TG-G-------------------------IYD-G--D--CS---------

PtPap-23 SALDFQLY--TG-G-------------------------IYD-G--D--CS---------

AtPap-16 GGFDFQFY--SS-G-------------------------VFT-G--E--CT---------

HvPap-17 GDSVFRFY--DS-G-------------------------VLG----GSGCG---------

OsPap-12 AGYVFRFY--DR-G-------------------------VLG----GAGCG---------

PtPap-5 GGYDFQFY--KS-G-------------------------VFK-G--D--CG---------

PtPap-12 GGNDFQFY--KS-G-------------------------VFN-G--D--CG---------

PtPap-6 GGNDFRFY--KS-G-------------------------VFE-G--D--CG---------

PtPap-9 GGNDFQFY--KS-G-------------------------VFN-G--D--CG---------

OsPap-16 GDRHFQFY--KG-G-------------------------VLS-G--AAGCA---------

PtPap-18 GGSDFQFY--SS-G-------------------------VFT-G--Q--CG---------

PtPap-15 GGSDFQFY--SS-G-------------------------VFT-G--K--CG---------

PtPap-22 GGSDFQFY--SS-G-------------------------VFT-G--K--CG---------

PtPap-11 GGSAFQFY--SS-G-------------------------VFT-G--D--CG---------

PtPap-26 GGSDFQFY--SS-G-------------------------IFT-G--S--CD---------

PtPap-27 GGSDFQFY--SS-G-------------------------IFT-G--S--CD---------

PtPap-28 GGFEFQFY--SS-G-------------------------IFT-G--S--CG---------

PtPap-36 GGFEFQFY--SS-G-------------------------IFT-G--S--CG---------

OsPap-25 S--KFQFY--GG-G-------------------------VMA-G--E--CG---------

OsPap-15 GDMTFQFY--SG-G-------------------------VMT-G--S--CG---------

OsPap-14 GDMTFQFY--KG-G-------------------------VMT-G--S--CG---------

OsPap-32 GDMTFQFY--KG-G-------------------------VMT-G--S--CG---------

HvPap-8 SARAFQLY--SQ-G-------------------------IFD-G--P--CP---------

OsPap-29 SARAFQLY--SK-G-------------------------IFD-G--P--CP---------

AtPap-10 SERAFQLY--SS-G-------------------------IFS-G--P--CS---------

PtPap-34 SERAFQMY--SK-G-------------------------IFT-G--P--CS---------

OsPap-31 GSRDFQFY--KG-G-------------------------IFD-G--E--CG---------

SmPap-3 GGSSMQFY--RS-G-------------------------IFT-G--R--CG---------

PpPap-1 GGRDFQHY--QG-G-------------------------VFT-G--P--CG---------

PpPap-2 GGRDFQHY--QG-G-------------------------VFT-G--P--CG---------

PtPap-17 SGMALQFY--QS-G-------------------------VFT-G--E--CG---------

PtPap-19 SGMALQFY--QS-G-------------------------VFT-G--E--CG---------

SmPap-1 SGRNFQLY--SG-G-------------------------VYT-G--H--CG---------

AtPap-5 SSQAFQLY--KS-G-------------------------VMT-G--T--CG---------

PpPap-4 GGRDFQLY--SG-G-------------------------VFT-G--R--CG---------

PpPap-3 GGRDFQLY--SG-G-------------------------VFT-G--E--CG---------

PpPap-5 GGRDFQLY--AQ-G-------------------------VFS-G--E--CG---------

AtPap-3 GGRIFQHY--QS-G-------------------------IFT-G--S--CG---------

OsPap-24 GGRAFQLY--SS-G-------------------------IFT-G--K--CG---------

AtPap-4 GGRAFQLY--TS-G-------------------------VFT-G--T--CG---------

OsPap-19 GGREFQLY--DS-G-------------------------VFT-G--R--CG---------

HvPap-7 GGREFQLY--HS-G-------------------------VFS-G--R--CG---------

OsPap-18 GGREFQLY--HS-G-------------------------VFS-G--R--CG---------

PtPap-25 GGRAFQLY--QS-G-------------------------VFT-G--S--CG---------

PtPap-31 GGRGFQLY--QS-G-------------------------VFT-G--S--CG---------

PtPap-29 GGRNFQLY--NS-G-------------------------VFT-G--E--CG---------

AtPap-1 GGRAFQLY--DS-G-------------------------IFD-G--S--CG---------

AtPap-2 GGRAFQLY--SS-G-------------------------VFD-G--L--CG---------

HvPap-6 GGRAFQLY--KS-G-------------------------IFT-G--T--CG---------

OsPap-9 GDQSFQFY--SD-G-------------------------VFA-G--D--CG---------

HvPap-30 SGKAFMFY--SE-G-------------------------VFT-G--D--CG---------

HvPap-10 SGKAFMFY--SE-G-------------------------VFT-G--E--CG---------

HvPap-11 SGKAFMFY--SE-G-------------------------VFT-G--D--CG---------

OsPap-11 SGRNFQFY--SG-G-------------------------VFD-G--P--CG---------

OsPap-20 SGRHFQFY--SG-G-------------------------VFD-G--P--CG---------

HvPap-4 SGRHFQFY--SG-G-------------------------VFD-G--P--CG---------

HvPap-5 SGRHFQFY--SG-G-------------------------VFD-G--P--CG---------

AtPap-15 SGRDFQFY--KG-G-------------------------VFN-G--K--CG---------

PtPap-33 SGRDFQFY--SG-G-------------------------VFS-G--P--CG---------

AtPap-14 SGREFQFY--SG-G-------------------------VFD-G--R--CG---------

PtPap-32 SGRDFQFY--SG-G-------------------------VFD-G--H--CG---------

PtPap-35 SGRDFQFY--SG-G-------------------------VFD-G--H--CG---------

HvPap-14 SGSHFQFY--SE-G-------------------------VFA-G--K--CG---------

OsPap-30 GGSHFQFY--SE-G-------------------------VFA-G--K--CG---------

AtPap-12 GSSDFQLY--SE-G-------------------------VFI-G--E--CG---------

PtPap-7 GGKDLQFY--SE-A-------------------------IFT-G--D--CG---------

AtPap-11 GSSDFQFY--SE-G-------------------------VFT-G--S--CG---------

HvPap-9 SGQDFQFY--SE-G-------------------------VFT-G--E--CS---------

OsPap-28 SGQDFQFY--SE-G-------------------------VFT-G--E--CG---------

AtPap-13 GGSDFQFY--SE-G-------------------------VFT-G--R--CG---------

PtPap-8 GGSDFQFY--SE-G-------------------------VFI-G--E--CG---------

PtPap-10 GGSDFQFY--SE-G-------------------------VFT-G--E--CG---------

VcPap-14 R--G---------YHCAQ------------------------------------------

VcPap-7 -------------YHQMA------------------------------------------

CrPap-7 ----------LAGYHQVS------------------------------------------

VcPap-8 -------------YHQVL------------------------------------------

VcPap-13 -------------YHQVL------------------------------------------

VcPap-11 -------------YHQVL------------------------------------------

VcPap-9 ----------LAGYQQVV------------------------------------------

VcPap-10 -------------YHQVV------------------------------------------

OlPap-8 ---ARGE---NHGGHVME------------------------------------------

OtPap-9 KNHG---------GHVME------------------------------------------

OlPap-9 ---E------PLGQHATK------------------------------------------

OtPap-8 RELG---------NHATK------------------------------------------

VcPap-12 -------------GHCVL------------------------------------------

CrPap-9 P--Q------V--SHTVT------------------------------------------

CrPap-6 P--S------I--NHVVS------------------------------------------

VcPap-4 S--Q------I--NHVVS------------------------------------------

OlPap-7 ---D------FEINHIVS------------------------------------------

OtPap-7 ---E------I--NHIVS------------------------------------------

CrPap-10 T--E------L--DHDVE------------------------------------------

VcPap-6 T--E------L--DHDVE------------------------------------------

CrPap-8 S--DPSK---S--THAVV------------------------------------------

CrPap-11 S--DPMK---S--THAVV------------------------------------------

SmPap-19 -------------GHAVK------------------------------------------

PpPap-10 -------------GHAVK------------------------------------------

PpPap-9 -------------GHAVK------------------------------------------

PpPap-11 -------------GHAVK------------------------------------------

AtPap-32 -------------GHAVK------------------------------------------

AtPap-29 -------------GHAVK------------------------------------------

AtPap-30 -------------GHAVK------------------------------------------

PtPap-1 -------------GHAVK------------------------------------------

PtPap-2 -------------GHAVK------------------------------------------

PtPap-3 -------------GHAVK------------------------------------------

HvPap-20 -------------GHAVK------------------------------------------

HvPap-19 -------------GHAVK------------------------------------------

OsPap-45 -------------GHAVK------------------------------------------

OlPap-5 P--LGRGIESI--NHAAL------------------------------------------

OtPap-5 P--LGRGISSI--NHAVL------------------------------------------

OlPap-6 PAPHTSETMYTAINHAAL------------------------------------------

OtPap-6 PAYHTANTMYTSINHAVL------------------------------------------

CrPap-5 E--E---------QQGYL------------------------------------------

OlPap-4 ---PADSDPLLAINHAAV------------------------------------------

OtPap-4 ---PASSDPLLAINHAAV------------------------------------------

HvPap-31 N--D------V--DHCVQ------------------------------------------

OlPap-3 PDWANHP---NLINHAVV------------------------------------------

OtPap-3 PDWNRSP---NLINHAVV------------------------------------------

SmPap-13 V--T---------TTSLL------------------------------------------

SmPap-9 E--S------G--SLSVL------------------------------------------

CrPap-3 ---T--RMRDL--DHTVT------------------------------------------

VcPap-3 ---H--KMRDL--DHTVT------------------------------------------

CrPap-4 EDPDS-----M--DHVVL------------------------------------------

VcPap-5 ---Q--DPDSM--DHAIL------------------------------------------

SmPap-17 DSKA---------SLPVL------------------------------------------

HvPap-32 T--T------L--DHVMT------------------------------------------

SmPap-7 S--TGDT---G--LTAVL------------------------------------------

SmPap-11 E--DK-----P--RLAVL------------------------------------------

SmPap-15 ---DDQATPYL----AVL------------------------------------------

SmPap-16 ---DDQATPYL----AVL------------------------------------------

HvPap-12 T--TPDD---V--NHAVL------------------------------------------

OsPap-40 T--SPMD---V--NHAVL------------------------------------------

AtPap-28 N--TPMD---V--NHAVL------------------------------------------

AtPap-27 S--TPMD---V--NHAVL------------------------------------------

PtPap-4 S--TPMD---V--NHAVL------------------------------------------

PpPap-8 T--GPQT---V--NHAVL------------------------------------------

SmPap-10 T--GPDT---V--NHAVL------------------------------------------

HvPap-22 T--S------Q--NHVVT------------------------------------------

HvPap-24 K--T------T--THTVL------------------------------------------

OsPap-34 T---AP----L--NHGVVVVGYGVNTTPDKTKYWIVKNSWGKGWGEGGYIRMKRDVGTPG

OsPap-43 TPPP------L--NHAVL------------------------------------------

OsPap-42 ------------------------------------------------------------

OsPap-44 ---R------I-------------------------------------------------

OsPap-35 T--S------I--NHAVL------------------------------------------

OsPap-37 T--R------I--NHAVL------------------------------------------

OsPap-33 T--E------L--NHAVL------------------------------------------

OsPap-36 T--ETK----D--NHVVL------------------------------------------

OsPap-38 S--NPNV---D--NHVVL------------------------------------------

HvPap-16 C--K------TAQNHAMA------------------------------------------

SmPap-14 G--E------I--NHGVL------------------------------------------

SmPap-18 GGDN------M--NHAVL------------------------------------------

OlPap-2 A-ES------L--DHGVL------------------------------------------

OtPap-2 S-EA------L--DHGVL------------------------------------------

HvPap-3 R-AM------V--NHGVL------------------------------------------

OsPap-22 R-AW------V--NHGVL------------------------------------------

AtPap-26 K-RN------V--NHGVL------------------------------------------

PtPap-24 K-KW------L--NHGVL------------------------------------------

PpPap-6 K-RN------L--DHGVL------------------------------------------

PpPap-7 K-RN------L--DHGVL------------------------------------------

SmPap-8 K-HH------I--DHGVL------------------------------------------

HvPap-2 R--H------L--DHGVP------------------------------------------

OsPap-17 R--H------L--DHGVL------------------------------------------

HvPap-1 R--H------L--DHGVL------------------------------------------

OsPap-41 R--H------L--DHGVL------------------------------------------

AtPap-25 K--S------Q--DHGVL------------------------------------------

AtPap-23 R--R------L--NHGVL------------------------------------------

AtPap-24 R--R------L--NHGVL------------------------------------------

PtPap-13 R--R------L--DHGVL------------------------------------------

PtPap-14 R--R------L--DHGVL------------------------------------------

PtPap-16 K--R------Q--DHGVL------------------------------------------

PtPap-20 K--H------Q--DHGVL------------------------------------------

HvPap-21 F--Q------V--NHYVA------------------------------------------

OsPap-8 ----------KKRNHAVA------------------------------------------

SmPap-12 T--D------L--NHAVT------------------------------------------

SmPap-5 D--S------L--DHGVL------------------------------------------

SmPap-6 N--S------R--DHAVL------------------------------------------

OsPap-13 T--N------V--NHALT------------------------------------------

HvPap-25 T--A------L--AHAVT------------------------------------------

OsPap-27 T--R------L--AHAVT------------------------------------------

OsPap-39 T--C------L--AHAVT------------------------------------------

HvPap-15 T--K------L--NHAVT------------------------------------------

HvPap-13 T--R------L--NHGVT------------------------------------------

OsPap-26 T--R------L--NHGVT------------------------------------------

OsPap-7 R--R------L--NHAVT------------------------------------------

OsPap-2 P--K------P--NHAVT------------------------------------------

OsPap-10 P--T------T--NHAVT------------------------------------------

OsPap-5 ----------ASSNHAVT------------------------------------------

OsPap-6 ----------ASSNHAVT------------------------------------------

HvPap-28 ---S--DAARV--NHAVT------------------------------------------

OsPap-1 A--E--R---V--NHAVA------------------------------------------

OsPap-3 P--G--R---M--NHAVT------------------------------------------

OsPap-4 P--G--S---V--NHAVT------------------------------------------

HvPap-18 A--N------V--NHAVT------------------------------------------

HvPap-27 S--DAAR---V--NHAVT------------------------------------------

HvPap-29 S--DAAR---V--NHAVT------------------------------------------

OsPap-21 T--D------L--NHAMT------------------------------------------

OsPap-23 T--K------L--DHAVA------------------------------------------

AtPap-31 T--D------V--NHAVT------------------------------------------

AtPap-21 T--D------L--HHAVT------------------------------------------

AtPap-22 T--Q------L--THAVT------------------------------------------

AtPap-19 T--S------V--NHAVT------------------------------------------

AtPap-18 T--S------S--NHAVT------------------------------------------

AtPap-20 T--N------V--NHAVT------------------------------------------

OlPap-1 S--Q------V--DHGVL------------------------------------------

OtPap-1 S--Q------V--DHGVL------------------------------------------

SmPap-4 E--E------I--NHGVL------------------------------------------

AtPap-17 T--N------L--NHGVT------------------------------------------

CrPap-2 E--G------L--NHGVL------------------------------------------

VcPap-2 E--G------L--NHGVL------------------------------------------

AtPap-7 T--N------L--NHGVV------------------------------------------

AtPap-8 T--N------L--NHGVV------------------------------------------

AtPap-6 ---N------LWGDHNVL------------------------------------------

AtPap-9 T--N------L--DHALV------------------------------------------

CrPap-1 T--S------L--NHGVL------------------------------------------

VcPap-1 T--S------L--NHGVL------------------------------------------

SmPap-2 S--N------L--DHGVA------------------------------------------

PtPap-30 N--D------I--DHAIL------------------------------------------

PtPap-21 DDPDD-----I--DHAVL------------------------------------------

PtPap-23 DDPND-----I--DHAVL------------------------------------------

AtPap-16 T--Y------L--DHAVT------------------------------------------

HvPap-17 T--E------L--NHAIT------------------------------------------

OsPap-12 T--E------L--NHAVT------------------------------------------

PtPap-5 T--Y------L--DHAVT------------------------------------------

PtPap-12 T--Q------Q--NHAVT------------------------------------------

PtPap-6 T--N------L--NHGVT------------------------------------------

PtPap-9 T--Q------Q--NHAVT------------------------------------------

OsPap-16 T--E------L--DHAIT------------------------------------------

PtPap-18 T--E------L--DHGVT------------------------------------------

PtPap-15 T--E------L--DHGVT------------------------------------------

PtPap-22 T--E------L--DHGVT------------------------------------------

PtPap-11 T--D------L--DHGVT------------------------------------------

PtPap-26 T--Q------L--DHGVT------------------------------------------

PtPap-27 T--Q------L--DHGVT------------------------------------------

PtPap-28 T--Q------L--DHGVT------------------------------------------

PtPap-36 T--E------L--DHGVT------------------------------------------

OsPap-25 T--S------L--DHGVT------------------------------------------

OsPap-15 T--D------L--DHGIV------------------------------------------

OsPap-14 T--D------L--DHGIV------------------------------------------

OsPap-32 T--D------L--DHGIV------------------------------------------

HvPap-8 T--S------L--DHAVL------------------------------------------

OsPap-29 T--S------L--DHAIL------------------------------------------

AtPap-10 T--S------L--DHAVL------------------------------------------

PtPap-34 T--S------L--DHAVL------------------------------------------

OsPap-31 I--Q------P--DHALT------------------------------------------

SmPap-3 T--D------L--DHGVT------------------------------------------

PpPap-1 T--D------L--DHGVL------------------------------------------

PpPap-2 S--E------L--DHGVL------------------------------------------

PtPap-17 T--A------L--DHGVV------------------------------------------

PtPap-19 T--A------L--DHGVV------------------------------------------

SmPap-1 Y--E------L--DHGVV------------------------------------------

AtPap-5 I--S------L--DHGVV------------------------------------------

PpPap-4 T--D------L--DHGVL------------------------------------------

PpPap-3 T--D------L--DHGVL------------------------------------------

PpPap-5 T--D------L--DHGVL------------------------------------------

AtPap-3 T--N------L--DHAVV------------------------------------------

OsPap-24 T--A------L--DHGVA------------------------------------------

AtPap-4 T--S------L--DHGVV------------------------------------------

OsPap-19 T--N------L--DHGVV------------------------------------------

HvPap-7 T--Q------L--DHGVV------------------------------------------

OsPap-18 T--S------L--DHGVV------------------------------------------

PtPap-25 T--Q------L--DHGVV------------------------------------------

PtPap-31 T--Q------L--DHGVV------------------------------------------

PtPap-29 T--S------L--DHGVA------------------------------------------

AtPap-1 T--Q------L--DHGVV------------------------------------------

AtPap-2 T--E------L--DHGVV------------------------------------------

HvPap-6 T--A------L--DHGVA------------------------------------------

OsPap-9 T--D------L--DHGVA------------------------------------------

HvPap-30 T--E------L--DHGVA------------------------------------------

HvPap-10 T--E------L--DHGVA------------------------------------------

HvPap-11 T--E------L--DHGVA------------------------------------------

OsPap-11 T--R------L--DHGVT------------------------------------------

OsPap-20 E--Q------L--DHGVT------------------------------------------

HvPap-4 A--Q------L--DHGVA------------------------------------------

HvPap-5 A--Q------L--DHGVA------------------------------------------

AtPap-15 T--D------L--DHGVA------------------------------------------

PtPap-33 T--D------L--DHGVA------------------------------------------

AtPap-14 V--D------L--DHGVA------------------------------------------

PtPap-32 T--Q------L--DHGVA------------------------------------------

PtPap-35 T--E------L--DHGVA------------------------------------------

HvPap-14 T--E------L--DHGVA------------------------------------------

OsPap-30 T--E------L--DHGVA------------------------------------------

AtPap-12 T--Q------L--NHGVV------------------------------------------

PtPap-7 T--E------L--NHGVA------------------------------------------

AtPap-11 T--E------L--NHGVA------------------------------------------

HvPap-9 T--D------L--DHGVA------------------------------------------

OsPap-28 T--D------L--DHGVA------------------------------------------

AtPap-13 T--E------L--NHGVA------------------------------------------

PtPap-8 T--E------L--DHGVA------------------------------------------

PtPap-10 K--E------L--DHGVA------------------------------------------

VcPap-14 -------------------------LIGWD------D------------E-A--GYWLFK

VcPap-7 -------------------------VVGYN-----DT------------G----SYWIAK

CrPap-7 -------------------------VVGYN------D---T--------G----AFWIVK

VcPap-8 -------------------------CIGYN-----DT------------G----HYWIAK

VcPap-13 -------------------------VMGYS-----DE------------G----SYWIIK

VcPap-11 -------------------------VVGYN-----DI------------G----SYWIVK

VcPap-9 -------------------------TIGYN-----DT------------G----SYWIAK

VcPap-10 -------------------------TIGYN-----DT------------G----SYWIAK

OlPap-8 -------------------------VIGWG--TT-ES------------G-T--RYWKVY

OtPap-9 -------------------------VIGWG--KS-AE------------G-V--RYWKVY

OlPap-9 -------------------------LIGWG--VS-EE------------G-E--HYWWMV

OtPap-8 -------------------------LIGWG--VT-QE------------G-D--HYWIMV

VcPap-12 -------------------------FVGYD------D------------A-K--KLFKFR

CrPap-9 -------------------------VVGWG--GE-EG------------G-M--EFWVVR

CrPap-6 -------------------------VVGWG--VDPET------------D-V--EYWVIR

VcPap-4 -------------------------VVGWS---VDED------------G-V--EYWIVR

OlPap-7 -------------------------IVGWG---TADD------------G-T--KYWVVR

OtPap-7 -------------------------IVGWG--TA-KD------------G-T--KYWIVR

CrPap-10 -------------------------VVGWG---E-ED------------G-E--KYWIVR

VcPap-6 -------------------------VVGWG----VED------------G-V--KYWVVR

CrPap-8 -------------------------IVGFT---------------------P--DTFIIR

CrPap-11 -------------------------IVGYT------P-----------------DTFIIR

SmPap-19 -------------------------FIGWG--TT-DD------------G-K--DYWIVA

PpPap-10 -------------------------LIGWG--TT-DD------------G-V--DYWTIV

PpPap-9 -------------------------LIGWG--TT-DD------------G-V--DYWTIV

PpPap-11 -------------------------LVGWG--TT-DD------------G-V--DYWSMV

AtPap-32 -------------------------LIGWG---TSDD------------G-E--DYWLLA

AtPap-29 -------------------------LIGWG--TS-DD------------G-E--DYWLLA

AtPap-30 -------------------------LIGWG--TS-SE------------G-E--DYWLMA

PtPap-1 -------------------------LIGWG--TS-ED------------G-E--AYWLLA

PtPap-2 -------------------------LIGWG--TS-ED------------G-E--DYWLLA

PtPap-3 -------------------------LIGWG--TS-DD------------G-E--DYWLLA

HvPap-20 -------------------------LIGWG--TS-DA------------G-E--DYWLLA

HvPap-19 -------------------------LIGWG--TS-DA------------G-E--DYWLLA

OsPap-45 -------------------------LIGWG--TT-DA------------G-E--DYWLLA

OlPap-5 -------------------------VVGWG--TT-DD------------G-V--KYWEIK

OtPap-5 -------------------------VVGWG---VTKD------------G-I--KYWELK

OlPap-6 -------------------------LTGWGEEKM-PN------------G-EVVKYWEVK

OtPap-6 -------------------------LTGWGEETM-PN------------G-EIVKYWKLK

CrPap-5 ---------------------RQVALVGYG--TDAAS------------G-M--PYWLIR

OlPap-4 -------------------------LVGWG--YD-QQ------------SKQ--GYWIMK

OtPap-4 -------------------------LVGWG--YDDES------------K-Q--PYWIMK

HvPap-31 -------------------------AVGVD---A-SA------------G----GYWKVR

OlPap-3 -------------------------AVGFG--YD--K---T----------LDMEYVVIK

OtPap-3 -------------------------AVGYG--HD--D---D----------SDLDYVIIK

SmPap-13 -------------------------VVGYD--TL--N------------G-V--PYWIAK

SmPap-9 -------------------------VVGYG--SE--N------------G-D--KYWIIK

CrPap-3 -------------------------LFGYG--SQ--D------------G-K--DYWLVR

VcPap-3 -------------------------LYGYG--TT-AD------------G-K--DYWLVR

CrPap-4 -------------------------LVGYG---TTAQ------------G-V--DYWLIK

VcPap-5 -------------------------LVGYG--TT-PE------------G-V--DYWLIK

SmPap-17 -------------------------IVGYG--RT--Q---H--------G-V--EYWILK

HvPap-32 -------------------------LVGYG--TT--R---HNDPYGEPAG-V--DFWILK

SmPap-7 -------------------------IVGYG--SD--N------------G-D--DYWILK

SmPap-11 -------------------------IVGYG--SE--K------------G-E--DYWIIK

SmPap-15 -------------------------IVGYG--SE--N------------G-Q--DYWIIK

SmPap-16 -------------------------IVGYG----SEN------------G-Q--DYWIIK

HvPap-12 -------------------------AVGYG--VE--N------------G-V--PYWLIK

OsPap-40 -------------------------AVGYG--VE--N------------G-V--PYWLIK

AtPap-28 -------------------------AVGYG--VE--D------------D-V--PYWLIK

AtPap-27 -------------------------AVGYG--VE--D------------G-V--PYWLIK

PtPap-4 -------------------------AVGYG--VE--N------------G-I--PYWLIK

PpPap-8 -------------------------AVGYG--ED--E------------NGV--PYWIIK

SmPap-10 -------------------------AVGYN--RD--A------------P-V--PYWIIK

HvPap-22 -------------------------VVGYG--VT-GA------------G-E--EYWIAK

HvPap-24 -------------------------VVGYD--ID-AF------------Q-R--KYWILK

OsPap-34 GLCGITTPYGVYNGPCGTSVNHAVTTVGYG--VT--Q---D--------N-I--NYWIAR

OsPap-43 ------------------------------------------------------------

OsPap-42 ------------------------------------------------------------

OsPap-44 ------------------------------------------------------------

OsPap-35 -------------------------AVGYG--ATPDN--------------T--KYWIVK

OsPap-37 -------------------------AVGYG--VTLNN--------------T--KYWIVK

OsPap-33 -------------------------VVGYD--ET--E---D--------G-T--PYWIVK

OsPap-36 -------------------------VVGYG--VT--T---D--------N-I--KYWIVK

OsPap-38 -------------------------VVGYG--VT--T---D--------N-I--KYWIIK

HvPap-16 -------------------------LVGYG--TK-PD------------G-T--KYWIGK

SmPap-14 -------------------------LVGYG---V-EN------------G-L--RYWTFK

SmPap-18 -------------------------LVGYG-----MD------------GDK--PYWILK

OlPap-2 -------------------------IVGYG------S---SGFAPVRWAP-E--PYWIVK

OtPap-2 -------------------------IVGYG------S---SGFAPVRWQQ-E--PYWIVK

HvPap-3 -------------------------LVGYG------A---RGFSALRLGY-R--PYWLIK

OsPap-22 -------------------------LVGYG------E---RGFAALRLGH-R--PYWIIK

AtPap-26 -------------------------LVGYG------S---KGFSILRLSN-K--PYWIIK

PtPap-24 -------------------------LVGYG------A---RGYSILRFGY-K--PYWIIK

PpPap-6 -------------------------LVGYA------E---RGFAPARLAY-K--PYWIIK

PpPap-7 -------------------------LVGYA------E---HGFAPARLAY-K--PYWIIK

SmPap-8 -------------------------LVGYG------A---KGYAPIRFTE-K--PYWIIK

HvPap-2 -------------------------------------------------G----WLWI--

OsPap-17 -------------------------LVGYG------S---AGYAPIRFKE-K--PYWIIK

HvPap-1 -------------------------LVGYG------A---SGFAPSRFKE-K--PYWIIK

OsPap-41 -------------------------LVGYG------A---SGFAPIRLKD-K--AYWIIK

AtPap-25 -------------------------LVGFG------S---SGYAPIRLKE-K--PYWIIK

AtPap-23 -------------------------LVGYG------A---AGYAPARFKE-K--PYWIIK

AtPap-24 -------------------------LVGYG------S---AGFSQARLKE-K--PYWIIK

PtPap-13 -------------------------LVGYG------S---AAYAPVRMKE-K--PYWIIK

PtPap-14 -------------------------LVGYG------S---AGYSPVRMKE-K--PFWIIK

PtPap-16 -------------------------LVGYG------S---AGYAPIRFKE-K--PFWIIK

PtPap-20 -------------------------LVGYG------A---AGYAPIRFKE-K--PFWIIK

HvPap-21 -------------------------IVGYG------K---DAAT-----G-K--KYWIIK

OsPap-8 -------------------------VVGYG--TA-SD------------G-T--PYWLLK

SmPap-12 -------------------------IVGYG--TT--S---D--------G-K--KYWIVK

SmPap-5 -------------------------LIGYG------T---E--G-----G-M--PYWIIK

SmPap-6 -------------------------VIGYG------T---E--G-----G-M--PYWIIK

OsPap-13 -------------------------IVGYG------T---NHPDT----G-E--NYWIAK

HvPap-25 -------------------------VVGYG--VDAAT------------G-V--KYWLVK

OsPap-27 -------------------------VVGYG--TDASS------------G-A--KYWTIK

OsPap-39 -------------------------VVGTA------------------------PTRLPV

HvPap-15 -------------------------VVGYG--QQ--QQNGADSVHASAPG-A--KYWIVK

HvPap-13 -------------------------VVGYG--KD--E---VTRES----------YWIVK

OsPap-26 -------------------------VVGYG--QE--E---AAADGGAAGG-D--KYWIIK

OsPap-7 -------------------------VVGYG------A---AADG-----G-G--EYWLVK

OsPap-2 -------------------------LVGYC------Q---DGAS-----G-K--KYWIAK

OsPap-10 -------------------------LVGYC--QDGAS------------G-K--KYWVAK

OsPap-5 -------------------------LVGYC--QDGAS------------G-K--KYWVAK

OsPap-6 -------------------------LVGYC--QDGAS------------G-K--KYWLAK

HvPap-28 -------------------------IVGYC-----EE-----------FG-E--KFWIAK

OsPap-1 -------------------------IVGYC-----EG--------FG--G-D--KYWIAK

OsPap-3 -------------------------IVGYC-----EN--------IG--G-D--KYWIAK

OsPap-4 -------------------------IVGYC-----EN--------FG--G-E--KYWIAK

HvPap-18 -------------------------IVGYCEGPGE--------------G-N--KYWIAK

HvPap-27 -------------------------IVGYC-----EG--------PGE-D-N--KYWIAK

HvPap-29 -------------------------IVGYC---EE-------------FG-E--KFWIAK

OsPap-21 -------------------------AVGYG--TD-EH------------G-T--KYWLMK

OsPap-23 -------------------------VVGYG--AE--A---DGSG-----G-G--GYWIIK

AtPap-31 -------------------------IVGYG------T---M--S-----G-L--NYWVLK

AtPap-21 -------------------------IVGYG--MS-EE------------G-T--KYWVVK

AtPap-22 -------------------------IVGYG--VS-EE------------G-I--KYWLLK

AtPap-19 -------------------------LVGYG--TS-PE------------G-M--KYWLAK

AtPap-18 -------------------------FVGYG--TS-QD------------G-T--KYWLAK

AtPap-20 -------------------------FVGYG--TS-PE------------G-I--KYWLAK

OlPap-1 ---------------------------------------------------V-----VVK

OtPap-1 -------------------------VVGYG--FD--D---THHNATKHHK-RHRHFWKVK

SmPap-4 -------------------------IVGYG---T-ED------------G-L--DYWIVK

AtPap-17 -------------------------VVGYG-----VE------------GDQ--KYWIVK

CrPap-2 -------------------------AVGYD--TS-DK------------A-Q--PYWIVK

VcPap-2 -------------------------TVGYN--VS-QD------------G-E--KYWIVK

AtPap-7 -------------------------VVGYG----TEN------------G-R--DYWLVK

AtPap-8 -------------------------VVGYG----TEN------------G-R--DYWIVK

AtPap-6 -------------------------IVGYG--TS-SD---E--------G----DYWLIR

AtPap-9 -------------------------IVGYG----SEN------------G-Q--DYWIVR

CrPap-1 -------------------------VVGYG------K---DVTGS----G-S--NYWIVK

VcPap-1 -------------------------VVGYG------K---DPHF-----G----NYWIVK

SmPap-2 -------------------------IVGYG--TD-AG------------G-R--QYWLVK

PtPap-30 -------------------------IVGYG------S---E--N-----D-E--DYWIVK

PtPap-21 -------------------------IVGYG----SEN------------G-E--DYWIVK

PtPap-23 -------------------------IVGYG----SEN------------G-E--DYWIVK

AtPap-16 -------------------------AIGYG---ESTN------------G-S--KYWIIK

HvPap-17 -------------------------AAGYG--TA-SD------------G-T--KYWIMK

OsPap-12 -------------------------AVGYG--TA-SD------------G-T--GYWLMK

PtPap-5 -------------------------AIGYG--TN-SD------------G-T--NYWLVK

PtPap-12 -------------------------AIGYG--TD-SD------------G-T--DYWLVK

PtPap-6 -------------------------AIGYG--TD-SD------------G-T--DYWLVK

PtPap-9 -------------------------AIGYG--TD-ID------------G-T--DYWLVK

OsPap-16 -------------------------AVGYG--VA-SD------------G-T--KYWLMK

PtPap-18 -------------------------AVGYG--ET-SD------------G-T--KYWLVK

PtPap-15 -------------------------AVGYG--ET-SD------------G-T--KYWLVK

PtPap-22 -------------------------AVGYG--ET-SD------------G-T--KYWLVK

PtPap-11 -------------------------AVGYG---MSDD------------G-T--KYWLVK

PtPap-26 -------------------------AVGYG--VS--D------------G-S--KYWLVK

PtPap-27 -------------------------AVGYG--VS--D------------G-S--KYWLVK

PtPap-28 -------------------------AVGYG--IS--D------------G-T--KYWLVK

PtPap-36 -------------------------AVGYG--GS--D------------G-T--KYWLVK

OsPap-25 -------------------------VIGYG---AASD------------G-T--KYWLVK

OsPap-15 -------------------------AIGYG---KDGD------------G-T--QYWLLK

OsPap-14 -------------------------AIGYG---KASD------------G-T--KYWLLK

OsPap-32 -------------------------AIGYG---KASD------------G-T--KYWLLK

HvPap-8 -------------------------IVGYG---S-EG------------G-K--DYWIVK

OsPap-29 -------------------------IVGYG---S-EG------------G-K--DYWIVK

AtPap-10 -------------------------IVGYG---S-QN------------G-V--DYWIVK

PtPap-34 -------------------------IVGYG---S-EN------------G-V--DYWIVK

OsPap-31 -------------------------AVGYG------S---Y--Y-----G-Q--DYIIMK

SmPap-3 -------------------------NVGYG--KE--D------------G-K--AYWIIK

PpPap-1 -------------------------AVGYG---TDDD------------G-V--NYWIVK

PpPap-2 -------------------------AVGYG---TDDD------------G-V--NYWIVK

PtPap-17 -------------------------VVGYG----TEK------------G-L--DYWLVR

PtPap-19 -------------------------VVGYA----SEN------------G-L--DYWLVR

SmPap-1 -------------------------AVGYG--TS-KT----------PDG-VATDYWIVR

AtPap-5 -------------------------VVGYG------S-------TS---G-E--DYWIIR

PpPap-4 -------------------------AVGYG----SEK------------G-L--DYWIVK

PpPap-3 -------------------------AVGYG-----SE------------GSL--DYWIVK

PpPap-5 -------------------------AVGYG----TED------------G-V--DYWIVK

AtPap-3 -------------------------AVGYG------S-------EN---G-V--DYWIVR

OsPap-24 -------------------------AVGYG------T-------EN---G-K--DYWIVR

AtPap-4 -------------------------AVGYG--SE--G------------G-Q--DYWIVR

OsPap-19 -------------------------AVGYG--TDAAT------------G-A--AYWTVR

HvPap-7 -------------------------AVGYG--TE--N------------G-K--DYWIVR

OsPap-18 -------------------------AVGYG--TD--N------------G-K--DYWIVR

PtPap-25 -------------------------AVGYG--TE--N------------G-V--DYWVVR

PtPap-31 -------------------------TVGYG--TE--H------------G-V--DYWIVR

PtPap-29 -------------------------AVGYG--TE--K------------G-K--DYWIVR

AtPap-1 -------------------------AVGYG--TE--N------------G-K--DYWIVR

AtPap-2 -------------------------AVGYG--TE--N------------G-K--DYWIVR

HvPap-6 -------------------------AVGYG--TE--N------------G-K--DYWLVR

OsPap-9 -------------------------VVGYG---ETND------------G-T--EYWIVK

HvPap-30 -------------------------VVGYG---VAED------------G-K--AYWTVK

HvPap-10 -------------------------VVGYG---VAED------------G-K--AYWTVK

HvPap-11 -------------------------VVGYG---VAED------------G-K--AYWTVK

OsPap-11 -------------------------AVGYG--TA-SK------------G-H--DYIIVK

OsPap-20 -------------------------AVGYG----TSK------------G-Q--DYIIVK

HvPap-4 -------------------------AVGYG--SDKGK------------G-H--DYIIVR

HvPap-5 -------------------------AVGYG--SDKGK------------G-H--DYIIVK

AtPap-15 -------------------------AVGYG----SSK------------G-S--DYVIVK

PtPap-33 -------------------------AVGYG----SSS------------G-I--DYIIVK

AtPap-14 -------------------------AVGYG----SSK------------G-S--DYIIVK

PtPap-32 -------------------------AVGYG----STN------------G-L--DYIIVK

PtPap-35 -------------------------AVGYG----SAK------------G-L--DFIVVK

HvPap-14 -------------------------AVGYG--TT-VD------------G-T--KYWIVK

OsPap-30 -------------------------AVGYG------T---------TVDG-T--KYWIVR

AtPap-12 -------------------------IVGYG------E---T--KN----G-T--KYWIVR

PtPap-7 -------------------------LVGYG---TTQD------------G-T--KYWIVK

AtPap-11 -------------------------AVGYG----SER------------G-K--KYWIVR

HvPap-9 -------------------------AVGYG--AT-RD------------G-T--KYWIVK

OsPap-28 -------------------------AVGYG--IT-RD------------G-T--KYWIVK

AtPap-13 -------------------------VVGYG--TT-ID------------G-T--KYWIVK

PtPap-8 -------------------------VVGYG---TTLD------------G-T--KYWIVR

PtPap-10 -------------------------IVGYG---TTVD------------G-T--KYWIVR

VcPap-14 SSWGEDLGDNGFYRDIFAF--

VcPap-7 NSWGTGWGDKGFLLMSYNA--

CrPap-7 NSWG-----------------

VcPap-8 NSWGTSWGDNGFFLMSYTN--

VcPap-13 NSWGTNKGDKGYYRISYNA--

VcPap-11 NSWGTRWGDNGFIRISYSA--

VcPap-9 NSWGTGWGDNGFFRISYSA--

VcPap-10 NSWGTGWGDNGFFRISYSA--

OlPap-8 NSW-LNWGDQGYGKIAVG---

OtPap-9 NSW-LNWGERGYGEIAVGE--

OlPap-9 NSW-RNWGENGVSKVRMGEMN

OtPap-8 NSW-RNWGENGVGKVRMGEMS

VcPap-12 NSWGADWG-------------

CrPap-9 NNWGEAWGERGFMRLVTSA--

CrPap-6 NSWGEPWGESGFLKLVTSA--

VcPap-4 NSWGEPWGEAGFMKLVTSA--

OlPap-7 NSWGQYWGEMGFFRIIRGV--

OtPap-7 NSWGQYWGEMGYFRIIRGV--

CrPap-10 NSWGTYWGERGFFRVRRGD--

VcPap-6 NSWGTYWGEMGFFRVERGV--

CrPap-8 NSWGESWG-------------

CrPap-11 NSWGESWG-------------

SmPap-19 NSWNRSWGEDGFFQISRGS--

PpPap-10 NSWNTNWGEDGLFRIVRGN--

PpPap-9 NSWNTNWGEHGLFRIARGG--

PpPap-11 NSWNTNWGEDGTFRILRGK--

AtPap-32 NQWNRSWGDDGYFKIRRGT--

AtPap-29 NQWNRSWGDDGYFKIRRGT--

AtPap-30 NQWNRGWGDDGYFMIRRGT--

PtPap-1 NQWNRGWGDDGYFKIRRGT--

PtPap-2 NQWNRGWGDDGYFKIKRGT--

PtPap-3 NQWNRGWGDDGYFKIRRGT--

HvPap-20 NQWNRGWGDDGYFKIIRGK--

HvPap-19 NQWNRGWGDDGYFKIIRGK--

OsPap-45 NQWNRGWGDDGYFKIIRGT--

OlPap-5 NSYGPEWGDEGFFRLERGR--

OtPap-5 NSYGPKWGDQGFFKLERGR--

OlPap-6 NSFGSDWGEGGYFRVERGP--

OtPap-6 NSFGSDWGENGYFRLERGP--

CrPap-5 NSWG-----------------

OlPap-4 NSYDSDWGEDGYAKLSMEL--

OtPap-4 NSYDSDWGEEGYAKLSMEL--

HvPap-31 NSWGPDWGENGYIRLAYGK--

OlPap-3 NSWGTQWGENGYARIALQG--

OtPap-3 NSWGENWGEGGYARIAIQG--

SmPap-13 NSWGYGWGEGGYIRIKRQG--

SmPap-9 NSWGTEWGENGYMKLERNS--

CrPap-3 NSWSHFWGDDGYIKIVRGK--

VcPap-3 NSWAKFYGDDGYIRILRGS--

CrPap-4 NSWSKYWGMDGYARITRKG--

VcPap-5 NSWSKYWGMDG----------

SmPap-17 NWWGRSWGEGGYMRLERGK--

HvPap-32 NSWSTAWGEAGYMRLRRGA--

SmPap-7 NSRGTKWGEQGYMKIQRGT--

SmPap-11 NSWGTSWGENGYMRIQRGN--

SmPap-15 NSAGTSWGEQGYMRLQRGN--

SmPap-16 NSAGTNWGEQGYMRLQRGN--

HvPap-12 NSWGADWGDNGYFKMEMGK--

OsPap-40 NSWGADWGDNGYFKMEMGK--

AtPap-28 NSWGGEWGDNGYFKMEMGK--

AtPap-27 NSWGADWGDKGYFKMEMGK--

PtPap-4 NSWGEDWGDNGYFKMEMGK--

PpPap-8 NSWGADWGMNGYFNMEMGK--

SmPap-10 NSWGESFGLDGYFYMEMGK--

HvPap-22 NSWGQTWGQNGFFFMRRGA--

HvPap-24 NSWGRKWGHEGYMYMARDE--

OsPap-34 NSWGPRWGESGYIRMKRDI--

OsPap-43 NSWGANWAESGYIRMKRDV--

OsPap-42 ----TGWQS------------

OsPap-44 ---------------------

OsPap-35 NSWGTGWGEMGYIRMKRDI--

OsPap-37 NSWNTTWGESGYIRMKRDV--

OsPap-33 NSWGAGWGESGYIRMIRNI--

OsPap-36 NSWGEGWGESGYIRMKRDI--

OsPap-38 NSWGKTWGEYGYIRMERDI--

HvPap-16 NSWTAKWGDKGFIYLLRDS--

SmPap-14 NSWTDEFGENGYFRLCRGV--

SmPap-18 NSWSENYGEDGYFRLCRGF--

OlPap-2 NSWSPAWGEGGYYRICKDK--

OtPap-2 NSWSPAWGEGGYYRICKDK--

HvPap-3 NSWGAQWGEGGYYKLCRGR--

OsPap-22 NSWGKAWGEQGYYRLCRGR--

AtPap-26 NSWGKKWGENGYYKLCRGH--

PtPap-24 NSWGNHWGEKGYYRLCRGH--

PpPap-6 NSWGPNWGDNGYYKICRGH--

PpPap-7 NSWGPMWGDKGYYKICRGH--

SmPap-8 NSWGATWGEQGYYKICRGH--

HvPap-2 -SWLRTAPLQGETILDHKE--

OsPap-17 NSWGENWGEKGYYKICRGP--

HvPap-1 NSWGENWGDKGYYKICRGS--

OsPap-41 NSWGENWGEHGYYKICRGS--

AtPap-25 NSWGAMWGEHGYYKICRGP--

AtPap-23 NSWGETWGENGFYKICKGR--

AtPap-24 NSWGESWGENGFYKICKGR--

PtPap-13 NSWGESWGENGFYKICRGR--

PtPap-14 NSWGEKWGENGFYKICRGR--

PtPap-16 NSWGQNWGENGYYKICRGR--

PtPap-20 NSWGENWGENGYYKICRAR--

HvPap-21 NSFGQSWGMDGYMLMERGI--

OsPap-8 NSWATDWGENGYMRIAVDA--

SmPap-12 NSWGTEWGDDGYVYMQRDT--

SmPap-5 NSWGTSWGEDGFMKIERKD--

SmPap-6 NSWGTSWGEDGFMRIKKED--

OsPap-13 NSYGNLWGDNGFVYLAKDT--

HvPap-25 NSWGQTWGESGYIRMRRDI--

OsPap-27 NSWGQSWGERGYIRILRDV--

OsPap-39 PSTGPS---------------

HvPap-15 NSWGTTWGDKGYILMKRGT--

HvPap-13 NSWGEKWGDNGYLRMKKGI--

OsPap-26 NSWGKNWGDQGYIKMKKDV--

OsPap-7 NQWGTWWGEGGYMRVARGG--

OsPap-2 NSWGKTWGQQGYILLEKDV--

OsPap-10 NSWGKTWGEKGYILLEKDV--

OsPap-5 NSWGKTWGQQGYILLEKDV--

OsPap-6 NSWGKTWGQQGYILLEKDI--

HvPap-28 NSWSNDWGDQGYIYLAKDV--

OsPap-1 NSWGSDWGEQGYVYLAKDV--

OsPap-3 NSWSSDWGEQGYVYLAKDV--

OsPap-4 NSWSNDWGEQGYVYLAKDV--

HvPap-18 NSWSNDWGEQGYVYLAKDV--

HvPap-27 NSWSNDWGDQGYIYLAKDV--

HvPap-29 NSWSNDWGDQGYIYLAKDV--

OsPap-21 NSWGTDWGEGGYMKIARDV--

OsPap-23 NSWGTTWGDGGYMKLEKDV--

AtPap-31 NSWGESWGENGYMRIRRDV--

AtPap-21 NSWGETWGENGYMRIKRDV--

AtPap-22 NSWGESWGENGYMRIMRDV--

AtPap-19 NSWGKTWGENGYIRIRRDV--

AtPap-18 NSWGETWGEKGYIRIRRDV--

AtPap-20 NSWGETWGENGYIRIRRDV--

OlPap-1 NSWGNQWGEGGFIRMARRI--

OtPap-1 NSWGGTWGEGGFIRMARRI--

SmPap-4 NSWAATWGDGGFVKMQRNT--

AtPap-17 NSWGTGWGEEGYIRMERGV--

CrPap-2 NSWGGSWGEQGYFRLKMGE--

VcPap-2 NSWGAGWGEQGYFRLKMGV--

AtPap-7 NSRGITWGEAGYMKMARNI--

AtPap-8 NSRGDTWGEAGYMKMARNI--

AtPap-6 NSWGPEWGEGGYLRLQRNF--

AtPap-9 NSWGTTWGDAGYIKIARNF--

CrPap-1 NSWGAEWGDAGYIRLKMGS--

VcPap-1 NSWGPEWGDNGYIRLRMGA--

SmPap-2 NSWGSGWGESGYVRMERQG--

PtPap-30 NSWGTEWGMEGYFYIRRNT--

PtPap-21 NSWGTSWGIEGYFYIKRNT--

PtPap-23 NSWGTEWGMEGYFYIKRNT--

AtPap-16 NSWGTKWGESGYMRIQKDV--

HvPap-17 NSWGGSWGEGGYVRIRRGV--

OsPap-12 NSWGASWGEGGYVRIRRGV--

PtPap-5 NSWGTSWGESGYMRMQRGI--

PtPap-12 NSWGTSWGESGYTRMQRGI--

PtPap-6 NSWGTSWGESGYTRMQRGI--

PtPap-9 NSWGTSWGENGYMRMRRGI--

OsPap-16 NSWGTSWGEDGYVRMERGV--

PtPap-18 NSWGTSWGEEGYIRMQRDT--

PtPap-15 NSWGTSWGEEGYIRMQRDI--

PtPap-22 NSWGTSWGEEGYIRMQRDI--

PtPap-11 NSWGTSWGENGYIRMERDI--

PtPap-26 NSWGAQWGEEGYIRMQKDI--

PtPap-27 NSWGAQWGEEGYIRMQKDI--

PtPap-28 NSWGAQWGEEGYIRMQKDI--

PtPap-36 NSWGAQWGEEGYIRMQKDI--

OsPap-25 NSWGTTWGEAGYLRMEKDI--

OsPap-15 NSWGTTWGENGFLRMEKDI--

OsPap-14 NSWGTTWGENGFLRMEKDI--

OsPap-32 NSWGMTWGENGFLRMEKDI--

HvPap-8 NSWGERWGMKGYMHMHRNT--

OsPap-29 NSWGESWGMKGYMYMHRNT--

AtPap-10 NSWGKSWGMDGFMHMQRNT--

PtPap-34 NSWGTGWGMRGYMHMQRNS--

OsPap-31 NSWGKNWGEQGYFRIRRGT--

SmPap-3 NSWGSNWGEKGYVKMARNT--

PpPap-1 NSWGPSWGEKGYIRMERMG--

PpPap-2 NSWGPGWGEKGYIRMERFG--

PtPap-17 NSWGTEWGEHGYIKMQRNV--

PtPap-19 NSWGTEWGEHGYIKMQRNV--

SmPap-1 NSWGDAWGESGYIRLQRNV--

AtPap-5 NSWGLNWGDSGYVKLQRNI--

PpPap-4 NSWGEYWGESGYLRMQRNL--

PpPap-3 NSWGEYWGESGYLRMQRNI--

PpPap-5 NSWGEYWGESGYLRMKRNM--

AtPap-3 NSWGPRWGEEGYIRMERNL--

OsPap-24 NSWGKSWGESGYVRMERNI--

AtPap-4 NSWGSNWGESGYFKLERNI--

OsPap-19 NSWGPDWGENGYIRMERNV--

HvPap-7 NSWGPNWGEAGYLRMERNI--

OsPap-18 NSWGPKWGESGYVRMERNI--

PtPap-25 NSWGPAWGENGYIRMERNV--

PtPap-31 NSWGPAWGENGYIRMERDV--

PtPap-29 NSWGKSWGESGYIRMERNI--

AtPap-1 NSWGKSWGESGYLRMARNI--

AtPap-2 NSWGNRWGESGYIKMARNI--

HvPap-6 NSWGSVWGEDGYIRMERNI--

OsPap-9 NSWGTAWGEGGYIRMQRDS--

HvPap-30 NSWGPSWGEQGYIRVEKDS--

HvPap-10 NSWGPSWGEQGYIRVEKDS--

HvPap-11 NSWGPSWGEQGYIRVEKDS--

OsPap-11 NSWGSHWGEKGYIRMRRGT--

OsPap-20 NSWGPHWGEKGYIRMKRGT--

HvPap-4 NSWGAQWGEKGYIRMKRGT--

HvPap-5 NSWGGKWGEKGYIRMKRGT--

AtPap-15 NSWGPRWGEKGFIRMKRNT--

PtPap-33 NSWGPKWGERGYLRMKRNT--

AtPap-14 NSWGPKWGEKGYIRLKRNT--

PtPap-32 NSWGSKWGEKGYIRMKRNT--

PtPap-35 NSWGSKWGEKGFIRMKRNT--

HvPap-14 NSWGPEWGEKGYIRMKRDV--

OsPap-30 NSWGADWGEKGYIRMKRDV--

AtPap-12 NSWGPEWGEGGYVRIERGI--

PtPap-7 NSWGTDWGEKGYIRMQRGI--

AtPap-11 NSWGAEWGEGGYIKIEREI--

HvPap-9 NSWGEDWGEKGYIRMQRGV--

OsPap-28 NSWGEDWGERGYIRMQRGV--

AtPap-13 NSWGEEWGEKGYIRMQRGI--

PtPap-8 NSWGPEWGEKGYIRMQRGI--

PtPap-10 NSWGPEWGERGYIRMQRGI--

**Figure 2.** Comparison of the amino acid sequences of the legumain-like cysteine proteinases from algae to angiosperms. The alignment was generated using the MUSCLE program. At, *Arabidopsis thaliana*; Pt, *Populus tricocarpa*; Os, *Oryza sativa*; Hv, *Hordeum vulgare*; Pp, *Physcomitrella patens*; Sm, *Selaginella moellendorffii*; Cr, *Chlamidomonas reinhardtii*; Vc, *Volvox carteri*.

AtLeg-4 ------MSSPLGHFQILVFLHALLIFSAESRKTQLLNDNDVESSDKSAK-----------

OsLeg-5 --------MAMGVLLLMLMLMHLQVGLGL-GNGGLWQEFLRLPTENG-------------

CrLeg-1 -----------MLVAVPLARCLLGA-----WRTATLTPAAKKPTDFADG-----------

VcLeg-1 ------------------------------------------------------------

PtLeg-5 -----------------------------------------DSTTAE-------------

SmLeg-1 -----MGRFRLECVLWLLILAQQACAARKFEWKRDSSASDEFLEDQSAR-----------

OsLeg-1 -------MAARWCFALLLALSAAAAGA---GAKRTWEPVIRMPGEVVEEEVATVPRGSEG

HvLeg-1 -------MAPWWCFGLLLLLCALAGAD---ASKGRWDPVIRMPGEEEPA----TARGDGG

OsLeg-2 ------MAARCWVWGFVVALLAVAAAADGEEEEGKWEPLIRMPTEEGDD----AEAAAPA

HvLeg-2 -MDSRVPMAAWWVCGFLPLLAVAAAAA-----------AAEPPSESGHA-------PAPA

HvLeg-3 -------MAAWWVCGVLPLLEVAAAAA---------EPLIRLPTESGHA---PATVPAPA

AtLeg-3 -----MAKSCYFRPALLLLLVLLVHA----ESRGRFEPKILMPTEEANP-----------

PtLeg-3 --METHKAYFLSAILVLAMLSFLHVQSVQAARLSPVEPRILMPTGKDEP-----------

PtLeg-4 -------MGNLFHLPRKLFL-------------------IFSPEKDGPE-----------

SmLeg-2 -----------------------------------------MPSSEEVA-----------

OsLeg-4 -------MGRGLLCLLLLQLVGLVVAGG--GRWRWQEEFLRLPSSDE-------------

HvLeg-5 -------MARLPCSPLLLLLVLSSQLALLVA-----GEFLRLPSEKDVV-----------

OsLeg-3 MAARARLRLVLPPLAALLLFAHLAAVAVARPRWEEEGSNLRLPSERAVA-------AGAA

HvLeg-4 -----MAMASFRLLPLALLLSVAH------ARTPRLEPTIRLPSQRAAG-----------

PtLeg-1 -----MTGLATGAIFLLISLCGIAAAG-----RDTVGDVLRLPSEASRF--------FHN

PtLeg-2 -----MTRLIAGVIFLLISFCGIAV-----GVRDIVGDVLRLPSEASRF----FRPGKFN

AtLeg-1 --------MTTVVSFLALFLFLVAAVSGDVIKLPSLASKFFRPTENDDD-----------

AtLeg-2 -MATTMTRVSVGVVLFVLLVSLVAVS----AARSGPDDVIKLPSQASRF--------FRP

PpLeg-3 -------MALSVEGIFLILLCSLGAMA---VAAREWDGKIVMPTEEGSK-----------

PpLeg-4 -----------------------------------------MPIDFNSE-----------

PpLeg-1 -----------------------------------------MPTGEGHK-----------

PpLeg-2 -----------------------------------------MPTEEDAE-----------

AtLeg-4 ---------GTRWAVLVAGSNEYYNYRHQADICHAYQILRKGGLKDENIIVFMYDDIAFS

OsLeg-5 ---------GTKWALLIAGSKGYENYRHQADVCHAYQIMKKGGLKDQNIVVMMYDDIAYN

CrLeg-1 ----DDVTVRNHWALLVAGSAGWGNYRHQADVCHAYQVLLRGGLRPAHIVTMMYDDIAHD

VcLeg-1 ---------------------------HQADVCHAYQVLLRGGLRPAHIVVMMYDDIAYD

PtLeg-5 ---------GKQWAVLVAGSAGYENYRHQADVCHAYQILKKGGLKDENIIVFMYDDIAFH

SmLeg-1 ---------GTRWAVLLAGSAGYWNYRHQADVCHAYQLLRRGGMREENIVVFMYDDIANN

OsLeg-1 TEEEEKDGVGTRWAVLVAGSSGYGNYRHQADVCHAYQILRKGGLKEENIVVFMYDDIANN

HvLeg-1 SQEEEEDGVGTRWAVLVAGSSGYGNYRHQADICHAYQILRKGGVKEENIVVFMYDDIAKN

OsLeg-2 PAPAAADYGGTRWAVLVAGSSGYGNYRHQADVCHAYQILQKGGVKEENIVVFMYDDIAHN

HvLeg-2 PGPSAPGEGVTKWAVLVAGSSGYGNYRHQADVCHAYQILKKGGLKDENIVVFMYDDIANN

HvLeg-3 PGPSAPAEEVTKWAVLVAGSSGYENYRHQADVCHAYQILKKGGLKDENIVVFMYDDIANS

AtLeg-3 -ADQDEDGVGTRWAVLVAGSSGYGNYRHQADVCHAYQILRKGGLKEENIVVLMYDDIANH

PtLeg-3 EVDDDGEEIGSRWAVLVAGSSGYGNYRHQADVCHAYQLLRKGGIKEENMVVFMYDDIAMH

PtLeg-4 VDDDDGKEIGTRWAVLVAGSNGYGNYRHQADVCHAYQLLRKGGIKEENIVVFMYDDIAKH

SmLeg-2 --------SGTRWALLVAGSSGFGNYRHQADVCHAYQLLRSNGLKEENIVVMMFDDIANN

OsLeg-4 ---------TTRWAVLIAGSNGFYNYRHQADVCHAYQIMRKGGVEEQNIVVMMYDDIAHN

HvLeg-5 ---------GTRWAVLIAGSNGYYNYRHQADVCHAYQIMKKGGLKDENIIVFMYDDIANN

OsLeg-3 ADDAAEAAEGTRWAVLIAGSNGYYNYRHQADVCHAYQIMKRGGLKDENIIVFMYDDIAHN

HvLeg-4 --QEDDDSVGTRWAVLIAGSNGYYNYRHQADICHAYQIMKKGGLKDENIIVFMYDDIARN

PtLeg-1 DDNSDDDSTGTRWAILLAGSNGYWNYRHQADVCHAYQLLRKGGLKEENIIVFMYDDIAYN

PtLeg-2 DDNSDDDSSGTRWAILLAGSNGYWNYRHQADVCHAYQLLRQGGLKEENIIVFMYDDIADN

AtLeg-1 ---------STKWAVLVAGSSGYWNYRHQADVCHAYQLLKKGGVKEENIVVFMYDDIAKN

AtLeg-2 AENDDDSNSGTRWAVLVAGSSGYWNYRHQADICHAYQLLRKGGLKEENIVVFMYDDIANN

PpLeg-3 --DPQPTEDGQRWAVLVAGSSGYGNYRHQADVCHAYQILKKGGMKDENIVVFMFDDIAHN

PpLeg-4 -------LKGVRWAILIAGSSGYGNYRHQADICHAYQILKRGGLKEENIVVFMYDDIANN

PpLeg-1 ------GEEGTRWAILIAGSAGYWNYRHQADVCHAYQILKRGGLKDENIIVFMHDDIAYH

PpLeg-2 --------KGTRWAILIAGSSGYWNYRHQADVCHAYQILKRGGLKEENIVVFMYDDIAYS

****:*****:: .*: :::.:*.****

AtLeg-4 SENPRPGVIINKPDGEDVYKGVPKDYTKEAVNVQNFYNVLLGNESGVT-G--GNGKVVKS

OsLeg-5 PENPHKGVIINKPNGPNVYAGVPKDYNGNDVNKNNFLAVLLGKKSALT-GA-GSGKVISS

CrLeg-1 PENPYPGHVFNSPGGPDVYGGVRVDYRGSDVSAAVFLAVLEGNASALPPGTRGSGRVLAS

VcLeg-1 TQNPFPGQVFNSPGGPDVYDGVRVDYRGSDVNAATFLAVLEGNASAVPPGN-GTGRVIAS

PtLeg-5 VDNPRPGIIINKPFGHDVYAGVPKDYTGDNCTVDNLFAVLLGNKSALT-G--GSGKVVDS

SmLeg-1 FANPRPGVMINHPNGDNVYAGVPKDYTGDQVTVNNFLAVLRGDKEALQ-G--GSGKVVES

OsLeg-1 ILNPRPGVIVNHPQGEDVYAGVPKDYTGDEVTAKNFYAVLLGNKTAVT-G--GSRKVIDS

HvLeg-1 ALNPRPGVIINHPEGEDVYAGVPKDYTGEAVTAKNFYAVLLGNKTAVT-G--GSKKVIDS

OsLeg-2 ILNPRPGTIINHPKGGDVYAGVPKDYTGHQVTTENFFAVLLGNKTAVT-G--GSGKVIDS

HvLeg-2 PDNPRPGIVINHPKGKDVYAGVPKDYTGDQVTADNFYAVLLGNKTAVT-G--GSRKVINS

HvLeg-3 PENPRRGVVINHPKGKDVYHGVPKDYTGDQVTAKNFYAVLLGNKTAVT-G--GSRKVINS

AtLeg-3 PLNPRPGTLINHPDGDDVYAGVPKDYTGSSVTAANFYAVLLGDQKAVK-G--GSGKVIAS

PtLeg-3 HLNPRPGVIINHPQGDDVYAGVPKDYTGEQVNTENLYAVLLGNKSAVK-G--GSGKVVDS

PtLeg-4 EFNPRPGVIINHPQGDDVYAGVPKDYTGVQVTTENLYAVLLGNKSAVK-G--GSGKVVDS

SmLeg-2 TDNPRPGTIINHPQGSDVYAGVPKDYTGAAVTAENFLAVLLGDKNSTS-G--GTGKVVSS

OsLeg-4 PDNPRPGLIFNHPSGPDVYAGVPKDYTGDDVNVNNFLAVLLGNRSALT-GS-GSGKVVAS

HvLeg-5 RDNPRPGVIINHPKGGDVYAGVPKDYTGADVNTNNFLAALLGDKSKLT-GS-GSGKVVSS

OsLeg-3 PENPRPGVIINHPQGGDVYAGVPKDYTGKEVNVKNLFAVLLGNKTAVK-G--GSGKVLDS

HvLeg-4 PENPRPGVIINHPQGGDVYAGVPKDYTGKEVNVKNFFAVLLGNKTAVN-G--GSGKVVDS

PtLeg-1 SENPRRGVIINSPQGEDVYKGVPKDYTGEDVTVGNFFAAILGNKTALT-G--GSGKVVDS

PtLeg-2 PENPRPGVIINNPQGEDVYKGVPKDYTGPDVTVGNFFAAILGNKTALT-G--GSGKVIDS

AtLeg-1 EENPRPGVIINSPNGEDVYNGVPKDYTGDEVNVDNLLAVILGNKTALK-G--GSGKVVDS

AtLeg-2 YENPRPGTIINSPHGKDVYQGVPKDYTGDDVNVDNLFAVILGDKTAVK-G--GSGKVVDS

PpLeg-3 RHNPRPGVILNHPNGEDVYHGVPKDYTGKNVTVNNLLAVLLGDKKTLK-G--GSGKVVNS

PpLeg-4 EENPHRGKVFNKPYGPDVYPGVPKDYTGENITVSNFYAAILGDADATK-G--GSGKVVAS

PpLeg-1 PENPYPGTIINKPDGPDVYQGVPKDYTGSDVTVSNLYAAILGDKSAIE-G--GTGKVVDS

PpLeg-2 TENPHPGKIINKPDGPDVYQGVPKDYTGADVTVSNFYAALLGDKDAIK-G--GSGKVVNS

** * :.* * * :** ** ** . : .: *. * *. .*: *

AtLeg-4 GPNDNIFIYYADHGAPGLIAMPTG-DEVMAKDFNEVLEKMHKRK-KYNKMVI--YVEACE

OsLeg-5 GPNDHIFVYYSDHGSPGYVCMPSG-GNLHANDLSQALKNKNAAG-AYKNLVV--YVEACE

CrLeg-1 GPYDRLFVFYSDHGAPGVLGMPSG-SFLYADELVGALQRKWRHR-GYKEAVL--YIEACE

VcLeg-1 GPYDRVFMFYSDHGSPGVLGMPSG-DFLYADQLVGALVRKYGRG-GYKEAVL--YVE---

PtLeg-5 GPNDNIFIYYADHGAPGLVGMPIG-KDLYAKDLIQVLKKQQEAN-SYKSMVF--YLEACE

SmLeg-1 GPNDHIFVFYSDHGGPGVLGMPVT-PYLYAVDLVTTLQDMHDNN-KYKEMVL--YIEACE

OsLeg-1 KPNDHIFIFYSDHGGPGVLGMPNL-PYLYAADFMKVLQEKHASN-TYAKMVI--YVEACE

HvLeg-1 KSNDHIFIYYSDHGGPGVLGMPNL-PYLYAADFIKVLQEKHASN-TYAKMVI--YVEACE

OsLeg-2 KPEDHIFIYYSDHGGPGVLGMPNL-PYLYAGDFIKVLQKKHASN-SYSKMVI--YVEACE

HvLeg-2 KPNDHIFIYYTDHGAAGLLGMPNP-PDVYADNFIEVLRQKHASK-SYSKMII--YVEACE

HvLeg-3 KPEDHIFIYYTDHGGAGSLGMPNV-PFVYAGDFIKVLRQKHASK-SYSKMVV--YVEACE

AtLeg-3 KPNDHIFVYYADHGGPGVLGMPNT-PHIYAADFIETLKKKHASG-TYKEMVI--YVEACE

PtLeg-3 KPNDRIFLYYSDHGGPGVLGMPNM-PFLYAMDFIEVLKKKHASG-SYKEMVM--YIEACE

PtLeg-4 MPNDRIFLYYSDHGGPGVLGMPTM-PFLYAMDFIEVLKKKHASG-SYKEMVM--YIEACE

SmLeg-2 GPEDHVFLFYSDHGGPGVLGMPGE-SNLYANDLIDVIKKKHASG-GYREMVI--YIEACE

OsLeg-4 GPNDHVFVYYADHGGPGVLSMPADGEYLYADDLVKALKKKHAGG-GYKSLVV--YVEACE

HvLeg-5 GPDDHIFVYYADHGGPGILGMPEDEEYLYANDLVRTLEKKHAGGAGYKSLVF--YLEACE

OsLeg-3 GPNDHIFIFYSDHGGPGVLGMPTY-PYLYGDDLVDVLKKKHAAG-TYKSLVF--YLEACE

HvLeg-4 GPNDHIFVFYSDHGGPGVLGMPTY-PYLYGDDLVDVLKKKHAAG-TYKSLVF--YLEACE

PtLeg-1 GPNDHIFIYYTDHGGPGVLGMPTN-PYLYADDLIDVLKKKHASG-TYKSLVF--YLEACE

PtLeg-2 GPNDHIFIYYTDHGGPGVLGMPTN-PYLYADDLIDVLKKKHASG-TYKSLVF--YLEACE

AtLeg-1 GPNDHIFIYYSDHGGPGVLGMPTS-PNLYANDLNDVLKKKYASG-TYKSLVF--YLEACE

AtLeg-2 GPNDHIFIFYSDHGGPGVLGMPTS-PYLYANDLNDVLKKKHALG-TYKSLVF--YLEACE

PpLeg-3 GPNDHIFIYYSDHGGPGVLGMPTN-PNLYADDLLKTFKKMHEAK-TYKEMVFVVYIEACE

PpLeg-4 GPNDHVFIYYADHGGAGVLGMPND-PILYADEFVDTLKKKAAAG-TFKKMVI--YVEACE

PpLeg-1 GPNDHIFIYYSDHGGPGVLGMPNP-PNLYADDFVGILKKKAAAG-TFKELVI--YLEACE

PpLeg-2 GPNDHIFIYYTDHGGAGVLGMPTS-PNLYADDFVDTLKKKAAAG-TFKELVI--YLEACE

. *.:*::*:***..* : ** : . :: : : . :. *:*

AtLeg-4 SGSMFEG-ILKKNLNIYAVTAANSKESSWGVYCPESYP-PPPSEIGTCLGDTFSISWLED

OsLeg-5 SGSMFEGQLLPSNIGVYAMTASNATENSWATYC-------DTPEYNTCLGDLFSVAWMED

CrLeg-1 SGSMFEG-LLPPDIGAYATTASNAMESSWGTYCPGMSP-GPPPLFSTCLGDLYSVAWMEN

VcLeg-1 ------------------------------------------------------------

PtLeg-5 SGSMFEG-LLPSNWSIYAITAANGEESSYGIYCPGYYP-APPPEFLTCLGDVFSISWMED

SmLeg-1 SGSIFEG-LLPKNLNIFVTTASNAVESSWGTYCPGMEP-SPPPEYDTCIGDLYSVAWMED

OsLeg-1 SGSIFEG-LMPEDLNIYVTTASNAEESSWGTYCPGMEP-SPPSEYITCLGDLYSVSWMED

HvLeg-1 SGSIFEG-LMPADLNIYVTTASNAEESSWGTYCPGMEP-SPPSEYITCLGDLYSISWMED

OsLeg-2 SGSIFEG-LMPENLNIYVTTASNAVENSWGTYCPGEEP-SPPPEYITCLGDMYSVAWMED

HvLeg-2 SGSIFEG-LLPQDHNIYVTTAANAVEDSWAAYCPKMEI-PPPPEYCTCLGDAYSVSWMED

HvLeg-3 SGSIFEG-LMPRDHNIYVTTAANAEESSWAAYCPGMEI-PPPSEYYTCLGDAYSVSWMED

AtLeg-3 SGSIFEG-IMPKDLNIYVTTASNAQESSYGTYCPGMNP-SPPSEYITCLGDLYSVAWMED

PtLeg-3 SGSIFEG-IMPKDLNIYVTTASNAEESSWGTXCPGMDP-SPPSEYVTCLGDLYSVAWMED

PtLeg-4 SGSIFEG-IMPKDINIYVTTASNAEENSWGTYCPGMEP-SPPPEYFTCLGDLYSVSWMED

SmLeg-2 SGSMVEG-LLPLGLGLYVTTASNAIESSWGTYCPGMVP-SAPPEYDTCLGDLYSVAWMED

OsLeg-4 SGSIFEG-LLPSDISVYATTASNAEESSWGTYCPGDDHDAPAAEFDTCLGDLYSVAWMED

HvLeg-5 SGSIFEG-LLPGNISVYATTAANAEESSWGTYCPGDDEGAPPPEYDTCLGDLYSVAWMED

OsLeg-3 SGSIFEG-LLPNGINVYATTASNADESSWGTYCPGEYP-SPPPEYDTCLGDLYSVAWMED

HvLeg-4 SGSIFEG-LLPNDIGVYATTASNAEESSWGTYCPGEYP-SPPPEYDTCLGDLYSISWMED

PtLeg-1 SGSIFEG-LLPQGLNIYATTASNAEESSWGTYCPGENP-SPPPEYETCLGDLYSVAWMED

PtLeg-2 SGSIFEG-LLPQGLNIYATTASNAEESSWGTYCPGEYP-SPPPEYETCLGDLYSVAWMED

AtLeg-1 SGSIFEG-LLPEGLNIYATTASNAEESSWGTYCPGEDP-SPPSEYETCLGDLYSVAWIED

AtLeg-2 SGSIFEG-LLPEGLNIYATTASNAEESSWGTYCPGEEP-SPPPEYETCLGDLYSVAWMED

PpLeg-3 SGSIFQG-LLPKDLNIYATTAANAEESSWGTYCPGMFP-APLEEFDTCLGDLYSVAWMED

PpLeg-4 SGSIFDG-LLPTGLNIYVTTASDPDENSWGTYCPTMIP-PPPPEFGTCLGDLYSVSWMED

PpLeg-1 SGSIFEG-LLPEGLNIYVTTASNAVESSWGTYCPGMYP-SPPSEYGTCLGDLYSVAWMED

PpLeg-2 SGSIFEG-LLPEGLNIYVTTASNAEESSWGTYCPGMYP-PPPPEYDTCLGDLYSVAWMED

AtLeg-4 SDLHDMS--KETLEQQYHVVKRRVG--SDVPETSHVCRFGTEKMLKDYLSS-YIGRNPEN

OsLeg-5 ADARRPGD-PETLGQLYDIVAKRTNL-------SHVSRYGDLSLSSQPVSLYYLPPGPGT

CrLeg-1 ADVCDLT--QETLMAQYSIIRNRTSNNYTYSMGSHVMQYGSLAITREVAGD-YQGMHNRG

VcLeg-1 ------------------------------------------------------------

PtLeg-5 SDLHDMS--QETLQQQYEVVRRRTGFD--YEDRSHVMQYGNMELSKELLSS-YLGTNAAN

SmLeg-1 SEVHNLD--HERLKDQYNTVKARTSDANTYRMGSHVMKYGDTNMDKERLSL-YLGFDPAN

OsLeg-1 SETHNLK--EESIKKQYEVVKKRTSDMNSYGAGSHVMEYGDRTFKDDKLYL-YQGFDPAN

HvLeg-1 SETNNLK--EETIKKQYEVVKKRTSDMNSYSAGSHVMEYGDKTFKDEKLYL-YQGFNPAN

OsLeg-2 SETHNLK--KETIEDQYELVKKRTSNANKLNEGSHVMEYGDKTFKDEKLFL-YQGFNPAN

HvLeg-2 SETQDLK--KESIKQQYEVVKARTAPRNESSIGSHVMEYGDKTFKEDMLFL-YQGFDPAK

HvLeg-3 SETHNLK--KETIKQQYEVVKARTAPRNKSSIGSHVMEYGDKTFKDEMLFL-YQGFDPAK

AtLeg-3 SETHNLK--KETIKQQYHTVKMRTSNYNTYSGGSHVMEYGNNSIKSEKLYL-YQGFDPAT

PtLeg-3 SETHNLK--KETIKQQYHSVKERTSNYNAFTSGSHVMQYGNESLKGEKLFL-YQGFDPAS

PtLeg-4 SGKHNLR--RETIEQQYHSVKERTSNYNTFTSGSHVMQYGNKSIKGEKLYL-YQGFNPAS

SmLeg-2 SEVHNLK--RETLLQQYLDVKDRTSNHNTYEAGSHVMQYGDVELNSNPLSM-FLGFDPAI

OsLeg-4 AEAHQEGRLAETLRQQYRTVKNRTSDEGTYTLGSHVMQYGDMALAPQSLDLYYMDTSPAT

HvLeg-5 SDAHNLN--AESLKQQYERVRNRTSADGTYSLGSHVMQYGDLGLNDQSLFQ-YIGTNPAN

OsLeg-3 SDVHNLR--TESLKQQYNLVKERTSVQHTYYSGSHVMEYGSLELNAHHVFM-YMGSNPAN

HvLeg-4 SDVHNLR--TESLKQQYNLVKKRTAAQDSYSYGSHVMQYGSLDLNAEHLFS-YIGSNPAN

PtLeg-1 SDIHNLQ--TETLHQQYELVKRRTSNDNS-PYGSHVMQYGDVGLSKDNIFL-YMGTNPAN

PtLeg-2 SDIHNLR--TETLHQQYELVKRRTSYDNS-PYGSHVMQYGDVGLSKDDLFQ-YMGTNPAN

AtLeg-1 SEKHNLQ--TETLHEQYELVKKRTAGSGK-SYGSHVMEFGDIGLSKEKLVL-FMGTNPAD

AtLeg-2 SGMHNLQ--TETLHQQYELVKRRTAPVG-YSYGSHVMQYGDVGISKDNLDL-YMGTNPAN

PpLeg-3 TEVENLK--KETLRDQYMIVKSRTSNHNTYKSGSHVLEFGDLKMKPEELDQ-YLGYDPAN

PpLeg-4 AEMENLK--KETLNDQYRIVKSRTSDNDTYMTGSHVMQYGDIEIDAEEVER-YLGFDPAN

PpLeg-1 TEKENLK--KETLEDQYLIVKSRTSNHNTYRSGSHVMQYGDLKIDVEELER-YLGFDPAN

PpLeg-2 TEIENLK--KETLEDQYVIVKSRTSNHNTYRTGSHVMQYGDVKLDVEELAR-YLGYDPAN

AtLeg-4 -DNFT------FTESFSSPISNSGLVNPRDIPLLYLQRKIQKAPMGSLESKEAQKKLLDE

OsLeg-5 -STAS--------AVIDDEGR-VGGVNQRDAGLVYLWRKYYE-----EKSVEAWERLLRE

CrLeg-1 EEGGH----ISDPWSSAAAAQPHPHMPQRDADLAPLRHAATHAVT-SERRAAAAEALARE

VcLeg-1 ------------------------------------------------------------

PtLeg-5 -DNYA-----TNINIEEYPSMIPRAFDQREATLLHFWHKYQEAPDGSDKKAEAHKDLLRI

SmLeg-1 -ANLT-----------SYNKP-ASSIGQRDADLLHFWQKYKNSKENSLEKSKALQEFLDV

OsLeg-1 -AEVK--------NKLSWEGP-KAAVNQRDADLLFLWRRYELLHDKSEEKLKALREISDT

HvLeg-1 -TNIT--------NKLFWQAR-KAAINQRDADLLFLWRRYELLHEKSKEKVNVLREISET

OsLeg-2 -GNIT--------NELIWPVP-KATVNQRDADLLFMWKRYEQLNGVSEDKLRALREIEDT

HvLeg-2 -SSIR-------NRPLPMPSL-KGAINQRDADILFMWRKYEKLNAGSEEKQRALREVKET

HvLeg-3 -SSIT--------NRLPLPIL-KGAINQRDADVLFMWKKYEQLNGGSEEKHRALRDIKET

AtLeg-3 -VNLP-------LNELPVKSK-IGVVNQRDADLLFLWHMYRTSEDGSRKKDDTLKELTET

PtLeg-3 -VNFP-------PNNGHIGAR-MDVVNQRDAELVFLWQMYKRAEGGSEKKTQILNQIKET

PtLeg-4 -VNFP-------PNNVHIGGR-MDVVNQRDAELVFLWQMYKRSEDGSEKKTQILNQIKET

SmLeg-2 -ADGN--------GDLIIPSS-ANGVSQRDADLLHLWSKYRRAKDGSDSKREARERMMNA

OsLeg-4 ANDHK----LAAAGAKGSHSY-TVSVNQRDADLLYLWRKYRRAGEGTAEKVEARERLVQE

HvLeg-5 -DNAT----FVQSSSSSRQLP-GARVNQRDADLVHFWHKYRRSAEGSAEKVEARRRLVET

OsLeg-3 -DNAT----FVEDNSLPSFSR---AVNQRDADLVYFWQKYRKLPESSPEKNEARKQLLEM

HvLeg-4 -ENTT----FVEDNALPSLSR---AVNQRDADLVYFWQKYRKLAESSPAKNNARKQLLEM

PtLeg-1 -DNFT----FMDENLLRPRSK---AVNQRDADLVHFWDKYRKAPEGSSRKVEAQKQFVEA

PtLeg-2 -DNYT----FVEENSLRPHSK---VVNQRDADLVHFWTKYRKAPEGSSRKVEAQKQFVEA

AtLeg-1 -ENFT----FVNENSIRPPSR---VTNQRDADLVHFWHKYQKAPEGSARKVEAQKQVLEA

AtLeg-2 -DNFT----FADANSLKPPSR---VTNQRDADLVHFWEKYRKAPEGSARKTEAQKQVLEA

PpLeg-3 -ENVTGPIFLREYLAIRLGGVEERHINQRDADLVHYWHRYHKSKVGSTAKAEAELDLMRI

PpLeg-4 -ENVT---RPELPVSKAPATASGMHVMQREAELLHLWHKYHKAVDGSKKESAGM-ELTRT

PpLeg-1 -ENVT---KPGLSELSPVNSDIVTHVPQREADLVHLKHKFYNAKKGSLREANAASELAKT

PpLeg-2 -ENVT---KPELPEFLSAHTEILTHVDQREADLIHLRYKFRNAVKGSLREANAATELAKT

AtLeg-4 KNHRKQIDQSITDI--LRLSVKQT-NVLNLLTSTRTTG--QPLVDDWDCFKTL--VNSFK

OsLeg-5 MERRSRVDSSVDLI--GDILLGDS-SKKKLLHIRRPAG--QPLVDDWDCLKSM--VRTFE

CrLeg-1 QARRQSLDQSAVLASSGPMAGGVS-TGQAAVGMEQEQGAAQPLVDDWDCLRAM--VAAWS

VcLeg-1 ------------------------------------------------------------

PtLeg-5 HSHIRHVDRSLSHI--ASTLFGDE-NAANAMKHVRPSG--QPLVDDWDCLKGL--VEAYE

SmLeg-1 IGRRTQIDRSVELV--GSVLLGSE-SASQILNSVRPEG--HPLVDNWDCLKEM--VRVFE

OsLeg-1 VMHRKLLDSSVDLV--GKLLFGFG-NGPSVLQAVRPSG--QPLVDDWDCLKRM--VRIFE

HvLeg-1 VTHRKHLDSSIDFI--GKLLFGFE-NGPSMLETVRPSG--IPLVDDWDCLKRM--VRIFE

OsLeg-2 IAHRKHLDSSIDFI--GKLVFGFE-NGPLALEAARSSG--QPLVDNWDCLKKM--VRIFE

HvLeg-2 VLHRKHLDSSIDFI--GKLVFGFD-KGPSVLQAARGSG--QPLVDDWDCLRTM--VRVFE

HvLeg-3 VLHRKHLDSSIDFI--GKLVFGFD-KGPSMLQAARGSG--QPLVDDWDCLKRM--VRVFE

AtLeg-3 TRHRKHLDASVELI--ATILFGPT---MNVLNLVREPG--LPLVDDWECLKSM--VRVFE

PtLeg-3 MRHRTHLDSSMELI--GTLLLGPK-KGSTILKSVREPD--SPLVDDWRCLKSM--VRLFE

PtLeg-4 MRHRTQLDSSMELI--GTLLFGRK-KGSAILKSVREPG--SPLVDDWICLKSM--VRRFE

SmLeg-2 LAHRQHVDESVDRV--GERLFGSKAAASKVLSTVRGSG--LALVDDWTCLKSLASVQAFE

OsLeg-4 MGRRSRVDRSVEMI--GGLLLGGA-KHKQQVVRERAA-----LVEDWECLRSM--VRTFE

HvLeg-5 MARRSRVDSSVELI--GGLLFGSE-EGAKVLGTVRPAG--QPVVDDWGCLKSV--VRRFE

OsLeg-3 MAHRSHVDNSVELI--GNLLFGSE-EGPRVLKAVRATG--EPLVDDWSCLKSM--VRTFE

HvLeg-4 MGHRSHIDSSVELI--GNLLFGSA-GGPMVLKTVRPAG--EPLVDDWSCLKST--VRTFE

PtLeg-1 MSHRMHIDHSIKLI--GKLLFGIE-KASEVLNAIRPAG--QPLVDDWDCLKTL--VRTFE

PtLeg-2 MSHRMHIDHSIKLI--GKLLFGIE-KASEALNTVRPAG--QPLVDDWVCLKTL--VRTFE

AtLeg-1 MSHRLHVDNSILLI--GILLFGLE--GHAVLNKVRPSG--EPLVDDWDCLKSL--VRAFE

AtLeg-2 MSHRLHIDNSVILV--GKILFGIS-RGPEVLNKVRSAG--QPLVDDWNCLKNQ--VRAFE

PpLeg-3 LSHRMYIDKSVDLV--GRLLFGVE-AGPTTLSAVRPDG--LPLTDDWACLKSM--VSAFE

PpLeg-4 IAHRMHVDNSIKLI--GDHMFGLD-TSLLRLKAVRPAG--QVLVDDWSCLKAM--VRTFE

PpLeg-1 ILHRRHLDDSVRLI--GELLFAGE-DALQKLGAVRPAG--SVVVDDWACLKNM--VRIFE

PpLeg-2 IVHRKHLDDSVQLI--GEILFAGE-NALEKLTAVRPAG--SVVVDDWACLKTM--VRTFE

AtLeg-4 NHCGATV--HYGLKYTGALANICNMGVDVKQTVSA-IEQAC--------------SM---

OsLeg-5 AHCGPLG--QYGMKHTRAFANMCNAALDHNHMAKA-ASKAC---MHPPVITY--------

CrLeg-1 DSCGPMAADQYVMRHTRLLARLCNAQVPPALVAEALRGSGC---SGANTV----------

VcLeg-1 ------------------------------------------------------------

PtLeg-5 KQCGGLS--WYGKKYTRVIANMCNAGINVEQMIGA-STRAC---SS--------------

SmLeg-1 TKCGPLG--QYGMKHMRAFANLCNAGVDPERMKSA-AGATC-GGISYVDGHDT--ASHSS

OsLeg-1 SHCGPLT--QYGMKHMRAFANICNNGISGASMKEA-SIATC---SSHNSGRWS--SLVQG

HvLeg-1 SHCGSLT--QYGMKHMRAFANICNNGISGTSMKEA-SISTC--GGGHNSARLS--TLIQG

OsLeg-2 SQCGSLT--QYGMKYMRAFANICNNGVSEAKMMEA-SINAC---GRYNSARWS--PMTEG

HvLeg-2 SQCGSLT--QYGTRHMRAFANICNNGVSEAEMKEA-SISAC---DGYDMGSWG--SGTLW

HvLeg-3 SQCGSLT--QYGMKHMRAFANICNNGVSEAEMKEA-SISAC---GGYDMGRWN--PLVLG

AtLeg-3 EHCGSLT--QYGMKHMRAFANVCNNGVSKELMEEA-STAAC---GGYSEARYTVHPSILG

PtLeg-3 THCGSLT--QYGMKHMRAFANICNGGVSLASMEEA-CVAAC---SGHDAGELH--PSNQG

PtLeg-4 THCGSLT--QYGMKHMRAFANICNGGVSQASMEEA-CIAAC---SGHEFGDLR--PSDQG

SmLeg-2 TSCGLLG--QYGMKHMRAFANLCNEGVDVPRMAAA-SAEAC---SSTAQAL---------

OsLeg-4 DQCGSLG--QYGIKHMRSFANICNAGVPHHAMAKA-ASLAC---PSPPPLHL--------

HvLeg-5 ERCGPLT--QYGMKHMRSLANICNAGVREEVMDKA-ASQAC---AASPSSLII-------

OsLeg-3 AQCGSLA--QYGMKHMRSFANICNAGISAEAMAKV-AAQAC---TSIPSNPWS--STHRG

HvLeg-4 SQCGSLA--QYGMKHMRSFANMCNAGIVPEAMAKV-AAQAC---TSFPTNPWS--ATHKG

PtLeg-1 THCGSVS--QYGMKHMRSLANLCNAGIGKEQMAEA-SAQAC---VSFPSGPWS--TLHKG

PtLeg-2 THCGSIS--QYGMKHMRSLANLCNAGIVKEQMAEA-SAQAC---VSFPSGSWS--SLHKG

AtLeg-1 RHCGSLS--QYGIKHMRSIANMCNAGIQMRQMEEA-AMQAC---PTIPTSPWS--SLDRG

AtLeg-2 RHCGSLS--QYGIKHMRSFANICNAGIQMEQMEEA-ASQAC---TTLPTGPWS--SLNRG

PpLeg-3 LSCGELS--EYGMKHMRAFANICNAGVEPSKMSGV-AAEAC-AVSAFGSGTLQ--IPTTG

PpLeg-4 ASCGPLT--QYGMKHMRAFASICNAGIDLDTMKKA-TSQAC-GFSETDTDLRT--ADSPR

PpLeg-1 ASCGPLT--QYGMKHMRAFANICNAGINSSRMSLA-SLEVCKISTSVDLGIWS--PVTSG

PpLeg-2 ASCGPLT--QYGMKHMRAFANICNARIDPAKMAVA-SSEACKLSTSAGSGIWS--PVTSG

AtLeg-4 --------------

OsLeg-5 --------------

CrLeg-1 --------------

VcLeg-1 --------------

PtLeg-5 --------------

SmLeg-1 F-------------

OsLeg-1 YSA-----------

HvLeg-1 YSA-----------

OsLeg-2 GHSA----------

HvLeg-2 F-------------

HvLeg-3 HSA-----------

AtLeg-3 YSA-----------

PtLeg-3 YSA-----------

PtLeg-4 YSA-----------

SmLeg-2 --------------

OsLeg-4 --------------

HvLeg-5 --------------

OsLeg-3 FSA-----------

HvLeg-4 FSA-----------

PtLeg-1 FSA-----------

PtLeg-2 FSA-----------

AtLeg-1 FSA-----------

AtLeg-2 FSA-----------

PpLeg-3 FSA-----------

PpLeg-4 FSASAKEFVKTIEF

PpLeg-1 FSA-----------

PpLeg-2 FSA-----------

**Figure 3.** Comparison of the amino acid sequences of the cystatin proteins from algae to angiosperms. The alignment was generated using the MUSCLE program. At, *Arabidopsis thaliana*; Pt, *Populus tricocarpa*; Os, *Oryza sativa*; Hv, *Hordeum vulgare*; Pp, *Physcomitrella patens*; Sm, *Selaginella moellendorffii*; Cr, *Chlamidomonas reinhardtii*; Vc, *Volvox carteri*.

AtCYS2 ----------MATMLKVSLV--------LSLL----GFLVIAVVTPSAANPFR------K

PtCPI-7 -------MAKLMKSSVPFLV--------CFLV----LSTLLVSGVSGY-----------R

PtCPI-8 ------MARLVKSSAPLFVF--------LVLY----TLLVSGVSGN-------------G

HvCPI-9 ------MATSTAAAVSCLLL--------LATL----SIAAAEIGHPQAPAA--------A

OC-X -------MATSPMLFLVSLL--------LVLV----AAATGDEASPSNAAAPA------A

OC-V --MASKLYYAVAPLVLVLLL--------LAPL----SSARLAAAAAADDDGQWPAGGG-R

HvCPI-5 ------MARVIGASGACALL--------VVLL--VACAASAARTGPGAARQLWD-----D

OC-IV ------MAARCPVGVASVLL--------LIVLVTVASAASGARSGGGGGGGIRELRGGGA

HvCPI-3 --------MARRSGCSVGAA--------LLAL----AALLAASAVPGA-----------A

OC-III MLRRRGFCCCSGAPAAAAAA--------LLLL----AVAAAAPRA--------------A

SmCPI-2 -----------------------------------------------------------M

SmCPI-1 -----------------------------------------------------------M

PpCPI-5 ----------MAPNHKVSVL--------LAAV----CLFGIYLVGA-------------Q

AtCYS1 ------------------------------------MADQQ------------------A

PpCPI-2 ------------------------------------------------------------

PpCPI-1 ------------------------------------------------------------

PpCPI-3 ------------------------------------------------------------

PpCPI-4 ------------------------------------------------------------

HvCPI-2 -------MWKYRVLGSVAAL--------LLLL----AVVVPFTQTWTQSARDKAAMAEDA

OC-I -----MRKYRVAGLVAALLV--------LHSL----ATPSAQAEAHRAGGEGEEKMSSDG

HvCPI-1 ------------------------------------MAEAAHGGGLRG-----------R

OC-II ---MRASSLFAESVFTTSAAAGRRRCPRLAAV----PVTLFFSTGRGSPAMAEEAQQP-R

PtCPI-4 ------------------------------------------------------------

PtCPI-5 ------------------------------------------------------------

HvCPI-4 ----MRVAATRPVSSAPVAL--------LAAL----ALLFLVGSASLAIGTM-------A

OC-XII MRVAATTRPASSSAAAPLPL--------FLLLAVAAAAAALFLVGSASLAM--------A

AtCYS3 --------MESKTFWIVTLL--------LCGT----IQLAICRSEEKSTE---------K

AtCYS6 --------MRSRFLLFIVFF--------SLSL----FISSLIASDLGFCNE--------E

AtCYS7 -------MDMRRASMCMMLI--------CVSL----VLLSGFGQFVICSEEKGTYNDNVV

PtCPI-3 ------------------------------------------------------------

PtCPI-1 ----------VSPFLLLSLT--------FTVT----TTTPFHFSDD-------------K

PtCPI-2 --------MISPSFISPLLT--------LAVV--LAVTITPQISASGGFCQE-------K

OC-XI ------------MARHPGLL--------LILL----AAVAAVATTS-------------R

AtCYS4 --------MMMKSLICLSLI--------LLPL----VSVVEGLG---------------G

HvCPI-7 --------------MRTSLL--------LLIV----AGTLYAVTKP-------------G

HvCPI-12 -------------MRTTNFL--------CVIV----VAIIYAISAPATGCGELKGNKLWN

HvCPI-10 -------------MRTSSFL--------VIIV----VVFLYAISSPATGCGERIGNQLWS

HvCPI-13 -------------MRTSSFL--------VIIV----GVFLYAISSPATGCGERIGNQLWS

PtCPI-6 ---------MKRQILTLSLL--------FIAV----TVVVV------------------D

HvCPI-11 --------------MRTTLF--------LAAI----VIIVHVVATP-------------T

AtCYS5 -----------MTSKVVFLL--------LLSL----VVVLLPLYAS-------------A

OC-IX -------------MRTSSLV--------LFAA----VAVFGAACTAAAGDE---------

HvCPI-6 -------MQKNSTMGRPLLL--------LALL----ATALAATSALGR-----------R

HvCPI-8 ------------MARGRLLL--------LALL----AATVAAAAAAAGLGG--------R

OC-VIII ------------MARIPLLL--------ALLL----AVSAAAAAQVGGNRG--------H

OC-VII -----------MTMRTSSLL--------LAAV----AVVAIVAGA--------------T

OC-VI -----------MAMTTRTLL--------LAAV----CAAAALPR----------------

VcCPI-1 --------MKHYFLIFCTLI--------LFSS----AWVKATADTGD------------Q

CrCPI-1 ------------MAKRVLIA--------LAAF----VMLNAA-----------------T

AtCYS2 SVVLGG------KSGVPNIRTNR-EIQQLGRYCVEQFNQQAQNEQGNIGSIAKTDTAISN

PtCPI-7 GGMVGG------RSEVSDVKTNK-QVQELGRFSVKEFNSHRSL---------YWKGGGVG

PtCPI-8 GGMVGG------RAKVSDVKTNK-EIQELGRFSVKEFNNHRST---------YGKGGEVG

HvCPI-9 AGAVGG------RTEISDVGKNK-LVQSLGRFAVAEHNRRLGH-----GGSGNNGDPVQV

OC-X PVLVGG------RTEIRDVGSNK-AVQSLGRFAVAEHNRRLRH-----GGSGGPADPVPV

OC-V GRKVGG------RTDVEDVEGNR-EVQELGLFCVVEHNRRGGS------------ATRGR

HvCPI-5 GRKVGG------WTEVRDVEGDR-EVQELGRYSVEEHNRRREE-------GCEGGGAVCG

OC-IV GRRVGG------RTEVRDVEGDR-EVQELGRFSVEEHNRRRRS-----------RDCGDV

HvCPI-3 GFHLGGDESGLVRGMLAAVRERA-EAEDAARFAVAEHNRKQGS-----------------

OC-III GFHLGGDESVLVRGMLAAIRREQAEAEDAARFAVAEYNKNQGA-----------------

SmCPI-2 TTLVGA------PKPLKE--ANSLEAEEHAKFAVEEHNRQAN------------------

SmCPI-1 TTLVGA------PKPLKE--ANSLEAEEHAKFAVEEHNRQNPE----------------A

PpCPI-5 AMSVGG------PKDISNFPNSV-EIDELANFAVDQYKSRQNS---------------IA

AtCYS1 GTIVGG------VRDIDANANDL-QVESLARFAVDEHNKNENL-----------------

PpCPI-2 -MLSGG------KQEVDLQNSNNLEIDEAAKFAVAEHNDRENS---------------LE

PpCPI-1 --MLGG------KKEVDVQDTNSLEIDELANFAVAEHLKSQNS---------------LE

PpCPI-3 --MLGG------KKEVDVQDTNSLEIDELANFAVSEHNNRQNS---------------LE

PpCPI-4 --MLGG------KKEVDVQDTNSLEIDELANFAVSEHNARQNS---------------LE

HvCPI-2 GPLMGG------IEDSPMGQENDLDVIALARFAVSEHNKKANA-----------------

OC-I GPVLGG------VEPVGN--ENDLHLVDLARFAVTEHNKKANS-----------------

HvCPI-1 GVLLGG------VQDAPAGRENDLETIELARFAVAEHNAKANA-----------------

OC-II GVKVGG------IHDAPAGRENDLTTVELARFAVAEHNSKANA-----------------

PtCPI-4 MATVGG------ITEVEGTANSL-EIDSLARFAVDDYNKKQNS-----------------

PtCPI-5 -MATGG------IKEVGGSANSL-EIESLARFAVDDYNKKQNS-----------------

HvCPI-4 SHVLGG------KSENPAAANSL-ETDGLARFAVDEHNKRENA-----------------

OC-XII GHVLGG------AHDAPSAANSV-ETDALARFAVDEHNKRENA-----------------

AtCYS3 TMMLGG------VHDLRGNQNSG-EIESLARFAIQEHNKQQNK-----------------

AtCYS6 MALVGG------VGDVPANQNSG-EVESLARFAVDEHNKKENA-----------------

AtCYS7 KMKLGG------FSDSKNDWNGGKEIDDIALFAVQEHNRRENA-----------------

PtCPI-3 ---LGG------VHDCKGSQNSA-EIDSLARFAVQEHNKKENA-----------------

PtCPI-1 MATLGG------VHDSEASQNSV-EIENLARFAVDEHNKKENA-----------------

PtCPI-2 MATLGG------VHDSQSSQNSA-EIDSLARFAVDEHNKKENA-----------------

OC-XI AQWVGG------WNVIEDVAGNN-QIQRVGAWAVGKHNQLGTN----------------D

AtCYS4 GGGLGS------RKPIKNV-SDP-DVVAVAKYAIEEHNKESKE-----------------

HvCPI-7 VALDGP------WTPIMHT-SDP-HVHDLGNWTVVKHGKVAND-----------------

HvCPI-12 TIIENG------WEPIINI-NDQ-HIQGLGSWAVIEFSKRMNC-----------------

HvCPI-10 TTIDNG------WEPIGNI-NEK-HIQELGSWAVLEFIKHVNC-----------------

HvCPI-13 TTIDNG------WEPIRNI-NDK-HIQELGSWAVLEFIKHVNC-----------------

PtCPI-6 AALLGG------WSPIKDL-KDK-HVVEIAEFAVAEHNKEAKS-----------------

HvCPI-11 TAIPGG------WVNIKNI-ADP-HIQELGKWAVLEHTQLGGN----------------D

AtCYS5 AARVGG------WSPISNV-TDP-QVVEIGEFAVSEYNKRSES-----------------

OC-IX -----S------WKTIDA--NDR-HVQDVALWAVAETDWASAT----------------G

HvCPI-6 GVLLGG------WSPVKDV-NDP-HVQELGGWAVAQHASLAKD-----------------

HvCPI-8 GALVGG------WGPIPDV-KDA-HIQELGGWAVEQHASLASD-----------------

OC-VIII GPLVGG------WSPITDV-GDP-HIQELGGWAVERHASLSSD-----------------

OC-VII AATVGS------WEPVDI--NDP-HVQELGRWAVAEEDRGVAA----------------G

OC-VI -----G------WSPIKNI-DDP-HIQELGRWAITENNRVSPS-----------------

VcCPI-1 HHGLGA------VVEADV--DNP-AIRDAADYVTRTANTNNCN-------GLCASLKRTG

CrCPI-1 ATIVGG------SSKAAV--SDP-DVVHAANFVVSSANTNACS-------GLCAGLQKEG

. :

AtCYS2 P-LQFSRVVSAQKQVVA-GLKYY-LRIEVTQPN-------GS-TRMFDSVVVIQP-W-LH

PtCPI-7 K-LMFSEVVEAHKQVVS-GLKYY-LNIVATTQN-------GE-KRMFDSVVVVQP-G-LR

PtCPI-8 E-LMFSEVVEAQTQVVS-GVKYY-LKIEATTQS-------EE-KLMFDSVLVVKP-W-LR

HvCPI-9 Q-LAFTAVAAAQKQVIS-GVVYY-LKVIARAPAG------GGGDRPFDAVVVVKA-W-AK

OC-X K-LAFARVVEAQKQVVS-GVAYY-LKVAASARDPRGGAAAGG-DRVFDAVVVVKA-W-LK

OC-V G-LVFSRVVAAQTQVVS-GIKYY-LRIAAQEA--------DD-ELVFDAVVVVKA-W-VP

HvCPI-5 R-LEFARVVSAQRQVVS-GVKYY-LRVAAAEANGAGSNGVSD-GRVFDAVVVVKP-W-LQ

OC-IV R-LEFGRVVAAQRQVVS-GLKYY-LRVAAAEEGAAGQNG-GE-PRVFDAVVVVKP-W-LE

HvCPI-3 A-LEFTRVVDAKRQVVA-GTLHN-LMVEVVD-S-------GK-KSMYKAKVWVKP-W-QN

OC-III -ELEFARIVKAKRQVVT-GTLHD-LMLEVVD-S-------GK-KSLYSAKVWVKP-W-LD

SmCPI-2 --LCFKRVVSAQTQVVS-GTMFH-LSIEAHSEQ-------HG-TGVYDAKVWTKP-W-ES

SmCPI-1 N-LCFKRVVSAQTQVVS-GTMFH-LSIEAHSEQ-------HG-TGVYDAKVWTKP-W-ES

PpCPI-5 V-ITFSKVLSAKEQVVQ-GKMYY-FTIEVME-N-------GV-PKNYDAKVWVKP-W-EG

AtCYS1 T-LEYKRLLGAKTQVVA-GTMHH-LTVEVAD-G-------ET-NKVYEAKVLEKA-W-EN

PpCPI-2 K-LTFSKVVSCHMQVVA-GSMYY-LVIEVEE-G-------SS-IKLYEAKVWVKP-W-QN

PpCPI-1 G-MTFSKVVSCRKQVVQ-GTMYH-LVIEVEE-S-------GK-LSQYEAKVWVKP-W-EN

PpCPI-3 K-MNLSKVISCHKQVVS-GLMYH-FVLEVEQ-G-------SQ-PKQYEAKVWVKP-G-GA

PpCPI-4 K-MNFSKVVSCHKQVVS-GLMYH-FVIEVEE-G-------SQ-LKQYEAKVWVKP-G-GS

HvCPI-2 L-LEFENVVKLKKQTVA-GTMYY-ITIRVTE-G-------GT-KKLYEAKVWEKL-W-EN

OC-I L-LEFEKLVSVKQQVVA-GTLYY-FTIEVKE-G-------DA-KKLYEAKVWEKP-W-MD

HvCPI-1 L-LEFERLVKVRQQVVA-GCMHY-FTIEVKE-G-------GA-KKLYEAKVWEKA-W-EN

OC-II M-LELERVVKVRQQVVG-GFMHY-LTVEVKEPG-------GA-NKLYEAKVWERA-W-EN

PtCPI-4 V-LEFKRVLNAKQQVVA-GTIYY-ITFEVTE-G-------GH-KKVYEAKVWVKP-W-LN

PtCPI-5 L-LEFKRVVKTKQQVVS-GTMYY-ITFEVTD-G-------GH-RKVYEAKVWEKP-W-MN

HvCPI-4 L-LEFVRVVEAKEQTVA-GTLHH-LTLEALE-A-------GR-KKVYEAKVWVKP-W-LD

OC-XII L-LEFVRVVEAKEQVVA-GTLHH-LTLEALE-A-------GR-KKVYEAKVWVKP-W-LD

AtCYS3 I-LEFKKIVKAREQVVA-GTMYH-LTLEAKE-G-------DQ-TKNFEAKVWVKP-W-MN

AtCYS6 L-LEFARVVKAKEQVVA-GTLHH-LTLEILE-A-------GQ-KKLYEAKVWVKP-W-LN

AtCYS7 V-LELARVLKATEQVVA-GKLYR-LTLEVIE-A-------GE-KKIYEAKVWVKP-W-MN

PtCPI-3 I-LEFVRVLKAKEQVVA-GKLYH-LTVEATD-A-------GN-NKMYEVKVWVKP-W-MN

PtCPI-1 I-LEFARVVKAKEQVVA-GTMHH-LTIEAIE-A-------GK-KKIYEAEVWVKP-W-LN

PtCPI-2 I-LEFARVVKAKEQVVA-GTMHH-LTIEAVE-A-------GK-KKLYEAKVWVKP-W-LN

OC-XI R-LQFVRVVAAEEQVVQ-GSNYL-VVIDAASSR-------KKTRELYVAVVADLV-G-AT

AtCYS4 K-LVFVKVVEGTTQVVS-GTKYD-LKIAAKDGG-------GK-IKNYEAVVVEKL-W-LH

HvCPI-7 G-LRFQKVVCGAVQIVAEGMNYR-LTIQALQVG-------VK-VGMYKAEVFEKESPFIT

HvCPI-12 V-LKFNKVVSGRQQLVS-SMNYE-LIIDVTHFE-------GK-EGKYKAEVYEQE-W-TK

HvCPI-10 V-LKFNKVVSGRQQLVS-GMNYE-LIINASDFD-------GK-DGKYKAEVYEQK-W-AS

HvCPI-13 V-LKFNKVVSGRQQLVS-GMNYE-LIINASDFG-------GK-DGKYKAEVYEQK-W-TS

PtCPI-6 N-LMLESIVKGESQVVS-GTNYR-LVLAVK----------GRANATYQAVVYEKP-W-EN

HvCPI-11 G-LRFVKVVSAEEQIVN-GVNYQ-LVIDALRLD-------GS-HRTYKAVLFEKDSSNAK

AtCYS5 G-LKFETVVSGETQVVS-GTNYR-LKVAANDGD-------GV-SKNYLAIVWDKP-W-MK

OC-IX G-LTLNTVDGAEKRFEA-GVNYYRLTLEASSRV-------VAKYLRFQAVVYEE----GD

HvCPI-6 G-LLFRRVTRGEQQVVS-GMNYR-LFVVAADGS-------GK-RVTYLAQIYEH--W-SR

HvCPI-8 G-LRFRRVTRGEQQVVS-GMNYR-LFVDAADGS-------GR-SAPYVAEVYEQS-W-TN

OC-VIII G-LRFRRVTSGEQQVVS-GMNYR-LVVSASDPA-------GA-TASYVAVVYEQS-W-TN

OC-VII G-LTFERVTDGEKQVVA-GVNYR-LTLEASSSG-------AK-DGRYEAVVYEQDPR-SN

OC-VI DELTFHRVTGGEQQVVS-GMNYR-LEIEAASGG-------GDVTGSYGAVVFEQE-W-SN

VcCPI-1 K-LKLLEILSAKTQVVA-GILYK-MELLLEDEK-------GQ-QVLFTCSVWNRP-W-NT

CrCPI-1 E-LKLVKVLSASTQVVA-GVNVH-LELLMADDT-------GK-QTVVTSTVWSRP-W-LA

: : . . . . :

AtCYS2 SKQ----------LLGFTPVVSPVY---------------------

PtCPI-7 TTE----------LLTFEPSAKLMVRK-------------------

PtCPI-8 SKE----------LLAFEPSIGLRV---------------------

HvCPI-9 SKE----------LVSFMPSPK------------------------

OC-X SKE----------LVSFTPASSTK----------------------

OC-V SRE----------MVSFVPAAELPGY--------------------

HvCPI-5 SRS----------LVRFAPADA------------------------

OC-IV SRT----------LLTFAPAADSPNES-------------------

HvCPI-3 FKA----------VVEFRHAGDFQSESSVAS---------------

OC-III FKG---SRSSSVTLGDYHSQVGHCC---------------------

SmCPI-2 FKK----------LEDFK----------------------------

SmCPI-1 FKK----------LEEFKPSKQGNECSGSRA---------------

PpCPI-5 YKE----------LESFLPSAPSHYDSAVDSDAKSGDCYEKSEIAT

AtCYS1 LKQ----------LESFNHLHDV-----------------------

PpCPI-2 FKK----------LEEFKLKDAGV----------------------

PpCPI-1 FKK----------LEDFKPKEQEVF---------------------

PpCPI-3 SKK----------LEEFKPKDA------------------------

PpCPI-4 SRK----------LEEFKSVDGGL----------------------

HvCPI-2 FKQ----------LEEFKPVQDAAIA--------------------

OC-I FKE----------LQEFKPVDASANA--------------------

HvCPI-1 FKQ----------LQEFKPAA-------------------------

OC-II FKQ----------LQDFKPLDDATA---------------------

PtCPI-4 FKE----------VQEFKLVADAPCDSSA-----------------

PtCPI-5 FKE----------VQEFKLVGDAPSDS-------------------

HvCPI-4 SKE----------LQEFRHTGDA-----------------------

OC-XII FKE----------LQEFRNTGDA-----------------------

AtCYS3 FKQ----------LQEFKESSS------------------------

AtCYS6 FKE----------LQEFKPASDAPAI--------------------

AtCYS7 FKQ----------LQEFKNII-------------------------

PtCPI-3 FKQ----------LQEFKHVEGG-----------------------

PtCPI-1 FME----------LKEFKHAGDV-----------------------

PtCPI-2 FKE----------LHEFKDAGDVPVF--------------------

OC-XI TYQ----------LSSFKLATK------------------------

AtCYS4 SKS----------LESFKAL--------------------------

HvCPI-7 IRK----------LISFRRANQAIDLEH------------------

HvCPI-12 KRQ----------LLSFTKVN-------------------------

HvCPI-10 KRE----------LHSFTKVN-------------------------

HvCPI-13 KRE----------LLSFTKVN-------------------------

PtCPI-6 LKS----------LTSFQPFKG------------------------

HvCPI-11 TRK----------FISFTPAN-------------------------

AtCYS5 FRN----------LTSFEPANNGRFL--------------------

OC-IX EHK----------LVSFVPIH-------------------------

HvCPI-6 TRK----------LTSFKPAAGG-----------------------

HvCPI-8 TRQ----------LRSFKPAAN------------------------

OC-VIII TRQ----------LTSFKPAAAH-----------------------

OC-VII ARK----------LVSFEPIH-------------------------

OC-VI TRK----------LISFDKNHNF-----------------------

VcCPI-1 GQNGGDDHHNHITKFHYQYIDP------------------------

CrCPI-1 SKNDAAQPATQITALTFKPLDGTLE---------------------

:

**Figure 4.** Comparison of the amino acid sequences of the carboxy terminal domain of long cystatins from the moss to angiosperms. The alignment was generated using the MUSCLE program. At, *Arabidopsis thaliana*; Pt, *Populus tricocarpa*; Os, *Oryza sativa*; Hv, *Hordeum vulgare*; Pp, *Physcomitrella patens*; Sm, *Selaginella moellendorffii*.

SmCPI-2 PSKQGSDLFFLR-------------------LPVDDPVVINEAAEHALKGLQQRSNSLIP

PpCPI-1 EVFTSADLGVRPGPFMVLG-GNSAPPTDKQSVPTDDPVV-QEAAEHVIKTLQMGSNSLST

PpCPI-2 AGVTSADLGVRTGGPHSTGRGISAPPSGKQSWPTDDLVV-QEAAEHAMKMLQQGSNSLAS

PpCPI-4 GGLTSADTGVKTEG------------WCDQIVPTEAPIV-QEAAEHAIKMLQQGSNSLSS

AtCYS7 PSFTISDLGFKPDG--------NGFDW--RSVSTNNPEV-QEAAKHAMKSLQQKSNSLFP

PtCPI-3 EGGTSSDLGVKPDD--------HGSGW--QPVPTNDLEV-QDAANHAVKSIQKRSNSLSP

AtCYS6 PAITSSDLGCKQGE--------HESGW--REVPGDDPEV-KHVAEQAVKTIQQRSNSLFP

HvCPI-4 TSFTISDLGAKRGG--------HEPGW--RDVPVHDPVV-KDAASHAVKSIQERSNSLFP

OC-XII TTFTNADLGAKKGG--------HEPGW--RDVPVHDPVV-KDAADHAVKSIQQRSNSLFP

PtCPI-1 PVFTSSDLGVKRDG--------HGPGW--QSVPVHDPSV-QDAANHALKSIQQRSNSLFP

PtCPI-2 PVFTSSDLGVKRDG--------HAPGW--RAVPVHDPSV-QDAAVHALKSIQQRSNSLFP

:* . * : .* :.:* :* **** .

SmCPI-2 YELDHVVKAQAEEVEIDTHRPSDKVLMKVKRGSREEHVNAKLHRGDTG-WTLTSAHVL--

PpCPI-1 YELNEILSAEAELND---ETAQFDLLLKTKLGAKEQVFKAEVSRTGDGDWTVKHATIQ--

PpCPI-2 YELSEIVSADAELSD---ESADFELLLKIKRGAKEEHFKSEIHRTGDGDWSVKHVTLQ--

PpCPI-4 YELREIVSAEAQLKE---GSAVFDLLLKTKRGSKEENFKSEVHRAEDGSWSVKHATLE--

AtCYS7 YKLIDIILARAKVVE---ERVKFELLLKLERGNKLEKFMVEVMKDQTGKYE---------

PtCPI-3 YELVEILLAKAKVIE---DYAKFNLLLKLRRGIKEENFKVEVIKNMEGKFHVNLM-----

AtCYS6 YELLEVVHAKAEVTG---EAAKYNMLLKLKRGEKEEKFKVEVHKNHEGALHLNHAEQHHD

HvCPI-4 YELIEIVRAKAEVVE---DFAKFDIVMKLKRGTKEEKMKAEVHKNLEGAFVLNQMQPEHD

OC-XII YELLEIVRAKAEVVE---DFAKFDILMKLKRGNKEEKFKAEVHKNLEGAFVLNQMQQEHD

PtCPI-1 YELQEVVDANAEVED---DSAKFDMLLKVKRGSAEEKLKVVVHKNSEGSYHLNRMEPHV-

PtCPI-2 YELQEVVHANAEVVD---DSAKFDMLLKVKRGSTEEKFKVLVHKNNEGNYHLNQMEPHA-

*:* :: * *: .:::* * : . : . *

SmCPI-2 -----

PpCPI-1 -----

PpCPI-2 -----

PpCPI-4 -----

AtCYS7 -----

PtCPI-3 -----

AtCYS6 -----

HvCPI-4 ESSSQ

OC-XII ESSSQ

PtCPI-1 -----

PtCPI-2 -----

**Figure 5.** Comparison of the amino acid sequences of the carboxy terminal domain of long cystatins deduced from transcript assemblies of different Viridiplantae clades. The alignment was generated using the MUSCLE program. At, *Arabidopsis thaliana*; Pt, *Populus tricocarpa*; Os, *Oryza sativa*; Hv, *Hordeum vulgare*; Pp, *Physcomitrella patens*; Sm, *Selaginella moellendorffii*.

Helicosporidium_sp --GAHSMSPVFPS------------------QRKNSDDSDM-NDAIAFAVRSISAQSNSI

Scenedesmus_obliquus -------------AGD----------DQW-------------SKAAQQGVAQLNQRSNSL

Adiantum_capillus-veneris --GSTQPSHMEPNSGE--------AGSA---QQLSPNDAGV-HEAAEQALKTLQQRSNSL

Pseudotsuga_menziesii -LSVNSRSSRALSQGK---LD-GAQAPLG--DAVGAQDPIV-KEAAENAVKIIQQRSNSL

SmCPI-2 --PSKQGSDLFF-------------------LRLPVDDPVVINEAAEHALKGLQQRSNSL

Ceratodon_purpureus -ESSFTTADLGAHIGD------GSNHMGL--RVVPSEDPAV-KEAAKEALKHIQARSNSL

Marchantia_polymorfa1 SLEHFKPQESSANDSP------NTEVAGM--RSVPVGDPVI-KEAAEHALKGLNDRSNSL

Marchantia_polymorfa2 --PQESS------------------ATGM--RSVPVGDPVI-KEAAEHALKGLNDRSNSL

PpCPI-1 --EVFTSADLGVRPGPFMVLG-GNSAPPTDKQSVPTDDPVV-QEAAEHVIKTLQMGSNSL

PpCPI-2 --AGVTSADLGVRTGGPHSTGRGISAPPSGKQSWPTDDLVV-QEAAEHAMKMLQQGSNSL

PpCPI-4 --GGLTSADTGVKTEG------------WCDQIVPTEAPIV-QEAAEHAIKMLQQGSNSL

AtCYS7 --PSFTISDLGFKPDG--------NGFDW--RSVSTNNPEV-QEAAKHAMKSLQQKSNSL

Pinus_taeda -QPSISSADLGVKQVH-------AEGLGW--RTVPVHDPVV-QEAAQHAVKNIQQRSNSL

Picea_glauca --PSVSRADLGVKQVH-------AEGSGW--RTVPVHDPVV-QEAAQHAVKNIQQQSNSL

Chamaecyparis_obtusa -----------VKREG--------LGSGW--REVPVHDPAV-KEAAEHAVSNIQQRSNSL

Chryptomeria_japonica ---SITTADLGVKHEG--------LGSGW--REVPVHDPVV-KEAAEHAVSNIQQRSNSL

Zamia_fischeri -----TAADLGVKQEG-------LLGTGW--RTVSVHDPVI-QEAAELAVKNIEQKSNSL

Ginkgo_biloba --NSVTPADLGVKHEG-------LQGSGW--RTVPAHDPVI-QEAAQHAVKNIQQKSNSL

PtCPI-3 --EGGTSSDLGVKPDD--------HGSGW--QPVPTNDLEV-QDAANHAVKSIQKRSNSL

AtCYS6 --PAITSSDLGCKQGE--------HESGW--REVPGDDPEV-KHVAEQAVKTIQQRSNSL

HvCPI-4 --TSFTISDLGAKRGG--------HEPGW--RDVPVHDPVV-KDAASHAVKSIQERSNSL

OC-XII --TTFTNADLGAKKGG--------HEPGW--RDVPVHDPVV-KDAADHAVKSIQQRSNSL

PtCPI-1 --PVFTSSDLGVKRDG--------HGPGW--QSVPVHDPSV-QDAANHALKSIQQRSNSL

PtCPI-2 --PVFTSSDLGVKRDG--------HAPGW--RAVPVHDPSV-QDAAVHALKSIQQRSNSL

Liriodendron_tulipifera --STFTRSDLGAKRGD--------HEPGW--KTVSAHDPVV-QDAANHAVKTIQQRSNSL

. : :. ***:

Helicosporidium_sp TPYALKSVANSRE---GDGPT---YYLTLVLSNPTLPDMRVNAEVSGKNRN-FKLTKWKQ

Scenedesmus_obliquus FPYQLLRVLSA-TPLNEGSSN---TELLVEVKRGDKQE-KFALTVKPAADAHFQLVKFHQ

Adiantum_capillus-veneris FPYELKKVTSFSAQVNGMQTC---YNLTLNVQRGSKEE-QLEARVARTNDGHWTVDNLHI

Pseudotsuga_menziesii MPYKLQEIVSAKEKVVNALKI---FDLLLKIKWENDVK-NYKVEMERTLEGKWTMKHMQS

SmCPI-2 IPYELDHVVKAQAEEVEIDTHRPSDKVLMKVKRGSREE-HVNAKLHRGDTG-WTLTSAHV

Ceratodon_purpureus IPYELKEVMQAHAEVNDEHTK---FHILLNVLRGPKEE-QFKAELTRTVLGEWSLKDLQQ

Marchantia_polymorfa1 VPYELRQVMTAHAEATDEHTN---FDLHIKVARGAKEE-EMKAELHRTADGKWSLKHAGP

Marchantia_polymorfa2 VPYELRQVMTAHAEATDEHTN---FDLHIKVARGAKEE-EMKAELHRTADGKWSLKHAGP

PpCPI-1 STYELNEILSAEAELNDETAQ---FDLLLKTKLGAKEQ-VFKAEVSRTGDGDWTVKHATI

PpCPI-2 ASYELSEIVSADAELSDESAD---FELLLKIKRGAKEE-HFKSEIHRTGDGDWSVKHVTL

PpCPI-4 SSYELREIVSAEAQLKEGSAV---FDLLLKTKRGSKEE-NFKSEVHRAEDGSWSVKHATL

AtCYS7 FPYKLIDIILARAKVVEERVK---FELLLKLERGNKLE-KFMVEVMKDQTGKYE------

Pinus_taeda ATYELQEVLSAKAEIVDASSK---VDLHLKTKRGSKVE-EHKVEMHCNPDGKWILRSHLT

Picea_glauca AAYELQEVLSAKAEVVDASSK---VDLHLKTKRGSKVE-EHKVEMHCNPDGKWILRSLLT

Chamaecyparis_obtusa AAYMLQEILLAKAEVIDGFAK---FDLLLKTKRGVKEE-QHKVEMHRNLEGGWMLQSHST

Chryptomeria_japonica AAYMLQEILLAKAEVIDGFAK---FDLLLKTKRGVKEE-QHKVEMHRNLEGDWMLKSHST

Zamia_fischeri ASYELQEILLAKAEVIEDWTK---FDLLLKIKRGTKHE-RYKVEMLRRYDGRWALNDVQQ

Ginkgo_biloba ASYELQEVLLAKAEVIEESAK---FDLLLKIKRGGKEE-KHKVEMQQGHDGRWIMNHAKH

PtCPI-3 SPYELVEILLAKAKVIEDYAK---FNLLLKLRRGIKEE-NFKVEVIKNMEGKFHVNLM--

AtCYS6 FPYELLEVVHAKAEVTGEAAK---YNMLLKLKRGEKEE-KFKVEVHKNHEGALHLNHAEQ

HvCPI-4 FPYELIEIVRAKAEVVEDFAK---FDIVMKLKRGTKEE-KMKAEVHKNLEGAFVLNQMQP

OC-XII FPYELLEIVRAKAEVVEDFAK---FDILMKLKRGNKEE-KFKAEVHKNLEGAFVLNQMQQ

PtCPI-1 FPYELQEVVDANAEVEDDSAK---FDMLLKVKRGSAEE-KLKVVVHKNSEGSYHLNRMEP

PtCPI-2 FPYELQEVVHANAEVVDDSAK---FDMLLKVKRGSTEE-KFKVLVHKNNEGNYHLNQMEP

Liriodendron_tulipifera APYELLEILLAKAEVIEDSAK---FDMLLKVKRGSKEE-KLKVEVHKNTEGNFHLNQVQP

.* * : : : . :

Helicosporidium_sp IS-------

Scenedesmus_obliquus HAAEGPATS

Adiantum_capillus-veneris HS-------

Pseudotsuga_menziesii ---------

SmCPI-2 L--------

Ceratodon_purpureus HHEGAL---

Marchantia_polymorfa1 M--------

Marchantia_polymorfa2 M--------

PpCPI-1 Q--------

PpCPI-2 Q--------

PpCPI-4 E--------

AtCYS7 ---------

Pinus_taeda ---------

Picea_glauca ---------

Chamaecyparis_obtusa VH-------

Chryptomeria_japonica DLGH-----

Zamia_fischeri DIVY-----

Ginkgo_biloba DTVV-----

PtCPI-3 ---------

AtCYS6 HHD------

HvCPI-4 EHDESSSQ-

OC-XII EHDESSSQ-

PtCPI-1 HV-------

PtCPI-2 HA-------

Liriodendron_tulipifera HEHAD----
