# Supplementary material for: The origin and evolution of plant cystatins and their target cysteine proteinases indicate a complex functional relationship
Source: BMC Evol Biol. 2008 Jul 10;8:198. doi: 10.1186/1471-2148-8-198 (PMC2474614; doi:10.1186/1471-2148-8-198)

Additional file 2. Complete cladogram of cysteine proteinases C1A from algae to angiosperms. The amino acid sequences were analysed with the PhyML method. Approximated likelihood-ratio test values >80% are indicated. F, cathepsin F-like; H, cathepsin H-like (Al, algae); B, cathepsin B-like; L, cathepsin L-like (in brackets groups A to E; O means other); Al, algae cathepsins (in brackets groups A to D). At, *Arabidopsis thaliana*; Pt, *Populus tricocarpa*; Os, *Oryza sativa*; Hv, *Hordeum vulgare;* Pp, *Physcomitrella patens*; Sm, *Selaginella moellendorffii*; Cr, *Chlamidomonas reinhardtii*; Vc, *Volvox carteri*; Ol, *Ostreococcus lucimarinus*; Ot, *Ostreococcus tauri*. The cladogram is fragmented in three consecutive parts from left to right.

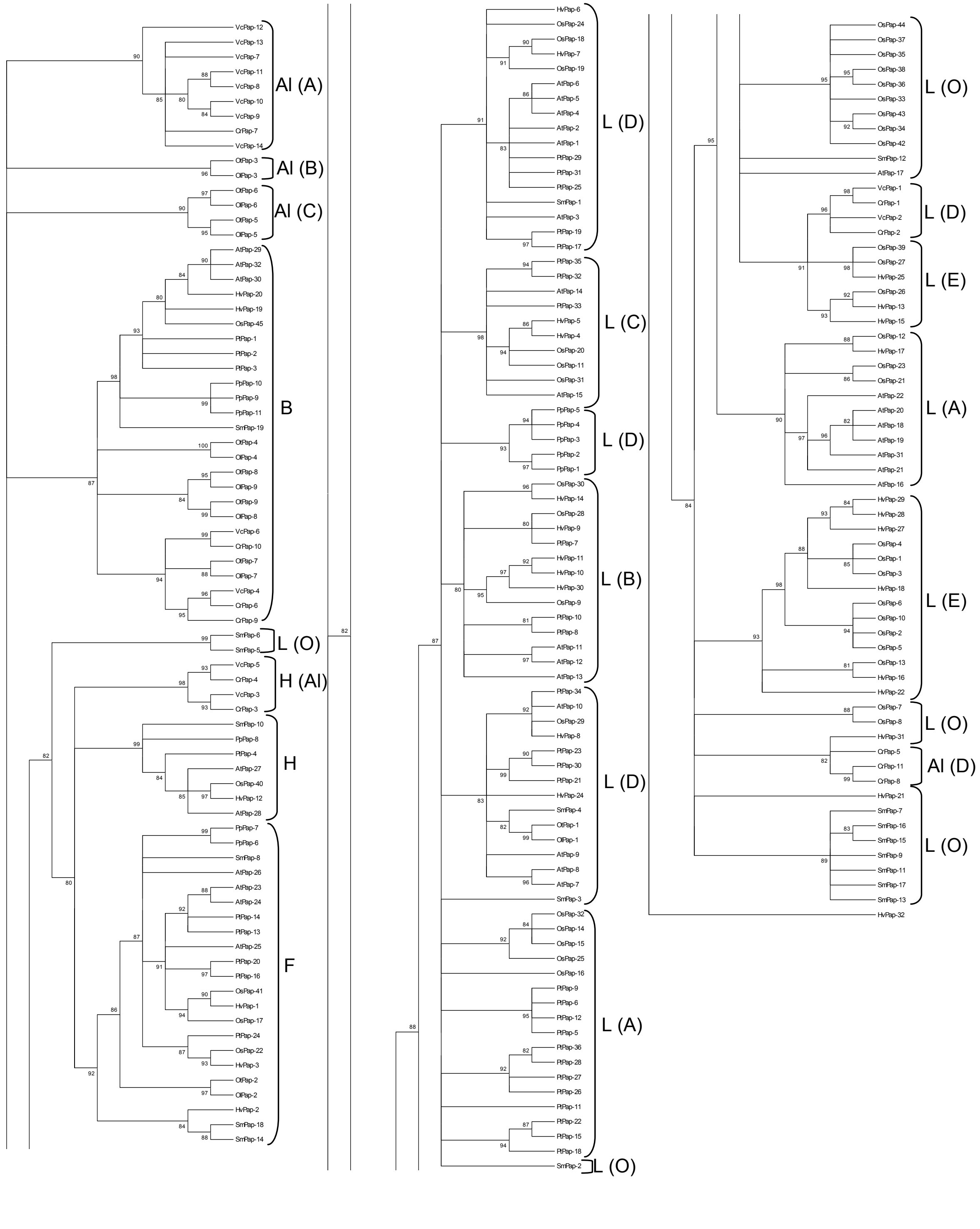

Supplement: Additional File 2 — Complete cladogram of cysteine proteinases C1A from algae to angiosperms from which Figure 1 is derived. The amino acid sequences were aligned by MUSCLE and analysed with the PhyML method. Approximate likelihood-ratio test values >80% are indicated. [file 1471-2148-8-198-S2.pdf]
