# Supplementary material for: The origin and evolution of plant cystatins and their target cysteine proteinases indicate a complex functional relationship
Source: BMC Evol Biol. 2008 Jul 10;8:198. doi: 10.1186/1471-2148-8-198 (PMC2474614; doi:10.1186/1471-2148-8-198)
Supplement: Additional File 3 — Information about gene models and accession numbers corresponding to the proteins used in this study. [file 1471-2148-8-198-S3.doc]

**Additional file 3.**

**Table I.** Information about the cystatin genes from *Volvox carteri,* *Chlamydomonas reinhardtii*, *Physcomitrella patens*, *Selaginella moegendorffii*, *Populus trichocarpa* *and Hordeum vulgare*. For cystatins from *Arabidopsis thaliana*, *Oryza sativa* and *Hordeum vulgare* (HvCPI-1 to 7) see Martinez et al [43].

| **Protein** | **Organism** | **Gene model/Accession number** |
| --- | --- | --- |
| VcCPI-1 | *V. carteri* | fgenesh4_pg.C_scaffold_109000020 |
| CrCPI-1 | *C. reinhardtii* | estExt_fgenesh2_kg.C_150044 |
| PpCPI-1 | *P. patens* | e_gw1.66.151.1 |
| PpCPI-2 | *P. patens* | e_gw1.212.78.1 |
| PpCPI-3 | *P. patens* | estExt_fgenesh1_kg.C_2380002 |
| PpCPI-4 | *P. patens* | estExt_gwp_gw1.C_2380025 |
| PpCPI-5 | *P. patens* | e_gw1.142.58.1 |
| SmCPI-1 | *S. moellendorffii* | fgenesh2_pg.C_scaffold_36000294 |
| SmCPI-2 | *S. moellendorffii* | gw1.79.98.1 |
| PtCPI-1 | *P. tricocarpa* | grail3.0100004601 |
| PtCPI-2 | *P. tricocarpa* | estExt_Genewise1_v1.C_LG_III0367 |
| PtCPI-3 | *P. tricocarpa* | gw1.1454.5.1 |
| PtCPI-4 | *P. tricocarpa* | eugene3.00011145 |
| PtCPI-5 | *P. tricocarpa* | gw1.IX.4482.1 |
| PtCPI-6 | *P. tricocarpa* | fgenesh4_pg.C_LG_VI000133 |
| PtCPI-7 | *P. tricocarpa* | grail3.0012015001 |
| PtCPI-8 | *P. tricocarpa* | grail3.0021016001 |
| HvCPI-8 | *H. vulgare* | AJ748343 |
| HvCPI-9 | *H. vulgare* | AJ748339 |
| HvCPI-10 | *H. vulgare* | AJ748342 |
| HvCPI-11 | *H. vulgare* | AJ748346 |
| HvCPI-12 | *H. vulgare* | AJ748347 |
| HvCPI-13 | *H. vulgare* | AJ748348 |

**Table II.** Information about the cystatin 3’ extended genes derived from EST Transcript Assemblies.

| **Organism/Protein** | **Transcript Assembly Accession** |
| --- | --- |
| Liriodendrom tulipifera | TA1347_3415 |
| Chamaecyparis obtusa | TA719_13415 |
| Gingko biloba | CB094462 |
| Zamia fischeri | TA392_34342 |
| Pinus taeda | TA2082_3352 |
| Picea glauca | TA14426_3330 |
| Pseudotsuga menziesii | CN636797 |
| Ceratodon purpureus | TA539_3225 |
| Marchantia polymorfa1 | TA397_3197 |
| Marchantia polymorfa2 | BJ864003 |
| Adiantum capillus-veneris | BP912121 |
| Scenedesmus obliquus | TA629_3088 |
| Helicosporidium sp | TA218_145475 |

**Table III.** Information about the legumain-like genes from *Volvox carteri,* *Chlamydomonas reinhardtii*, *Physcomitrella patens*, *Populus trichocarpa,* *Arabidopsis thaliana*, *Oryza sativa* *and Hordeum vulgare*.

| **Protein** | **Organism** | **Gene model/Accession number** | **Common name** |
| --- | --- | --- | --- |
| VcLeg-1 | *V. carteri* | gw1.36.243.1 |  |
| CrLeg-1 | *C. reinhardtii* | e_gwW.88.9.1 |  |
| PpLeg-1 | *P. patens* | e_gw1.114.190.1 |  |
| PpLeg-2 | *P. patens* | e_gw1.16.167.1 |  |
| PpLeg-3 | *P. patens* | estExt_gwp_gw1.C_360138 |  |
| PpLeg-4 | *P. patens* | fgenesh1_pm.scaffold_406000002 |  |
| SmLeg-1 | *S. moellendorffii* | e_gw1.0.1197.1 |  |
| SmLeg-2 | *S. moellendorffii* | e_gw1.84.202.1 |  |
| PtLeg-1 | *P. tricocarpa* | estExt_Genewise1_v1.C_LG_XVIII0730 |  |
| PtLeg-2 | *P. tricocarpa* | grail3.0013022501 |  |
| PtLeg-3 | *P. tricocarpa* | gw1.127.139.1 |  |
| PtLeg-4 | *P. tricocarpa* | estExt_fgenesh4_pg.C_LG_III0908 |  |
| PtLeg-5 | *P. tricocarpa* | gw1.VIII.2629.1 |  |
| HvLeg-1 | *H. vulgare* | AM941111 |  |
| HvLeg-2 | *H. vulgare* | AM941112 |  |
| HvLeg-3 | *H. vulgare* | AM941113 |  |
| HvLeg-4 | *H. vulgare* | AM941114 |  |
| HvLeg-5 | *H. vulgare* | AM941115 |  |
| OsLeg-1 | *O. sativa* | Os02g43010 |  |
| OsLeg-2 | *O. sativa* | Os04g45470 |  |
| OsLeg-3 | *O. sativa* | Os01g37910 |  |
| OsLeg-4 | *O. sativa* | Os05g51570 |  |
| OsLeg-5 | *O. sativa* | Os06g01610 |  |
| AtLeg-1 | *A. thaliana* | At2g25940 | AtVPEa |
| AtLeg-2 | *A. thaliana* | At1g62710 | AtVPEb |
| AtLeg-3 | *A. thaliana* | At3g20210 | AtVPEd |
| AtLeg-4 | *A. thaliana* | At4g32940 | AtVPEg |

**Table IV.** Information about the papain-like genes from *Ostreococcus tauri*, *Ostreococcus lucimarinus*, *Volvox carteri,* *Chlamydomonas reinhardtii*, *Physcomitrella patens*, *Populus trichocarpa,* *Arabidopsis thaliana*, *Oryza sativa* *and Hordeum vulgare*.

| **Protein** | **Organism** | **Gene model/Accession number** | **Common name** |
| --- | --- | --- | --- |
| OtPap-1 | *O. tauri* | gw1.12.00.194.1 |  |
| OtPap-2 | *O. tauri* | fgenesh1_pm.C_Chr_10.0001000033 |  |
| OtPap-3 | *O. tauri* | gw1.02.00.400.1 |  |
| OtPap-4 | *O. tauri* | gw1.01.00.538.1 |  |
| OtPap-5 | *O. tauri* | 0700010158 |  |
| OtPap-6 | *O. tauri* | 0700010155 |  |
| OtPap-7 | *O. tauri* | gw1.01.00.404.1 |  |
| OtPap-8 | *O. tauri* | estExt_fgenesh1_pg.C_Chr_06.00010106 |  |
| OtPap-9 | *O. tauri* | 1400010149 |  |
| OlPap-1 | *O. lucimarinus* | fgenesh1_pm.C_Chr_12000034 |  |
| OlPap-2 | *O. lucimarinus* | eugene.1000010116 |  |
| OlPap-3 | *O. lucimarinus* | gwEuk.2.352.1 |  |
| OlPap-4 | *O. lucimarinus* | gwEuk.1.659.1 |  |
| OlPap-5 | *O. lucimarinus* | fgenesh1_pg.C_Chr_7000132 |  |
| OlPap-6 | *O. lucimarinus* | fgenesh1_pg.C_Chr_7000129 |  |
| OlPap-7 | *O. lucimarinus* | e_gwEuk.1.880.1 |  |
| OlPap-8 | *O. lucimarinus* | gwEuk.20.217.1 |  |
| OlPap-9 | *O. lucimarinus* | C_Chr_6000057 |  |
| CrPap-1 | *C. reinhardtii* | e_gwW.35.77.1 |  |
| CrPap-2 | *C. reinhardtii* | e_gwW.6.71.1 |  |
| CrPap-3 | *C. reinhardtii* | e_gwH.10.83.1 |  |
| CrPap-4 | *C. reinhardtii* | e_gwW.95.7.1 |  |
| CrPap-5 | *C. reinhardtii* | Chlre2_kg.scaffold_21000087 |  |
| CrPap-6 | *C. reinhardtii* | e_gwH.78.33.1 |  |
| CrPap-7 | *C. reinhardtii* | Chlre2_kg.scaffold_62000057 |  |
| CrPap-8 | *C. reinhardtii* | estExt_fgenesh2_pg.C_170056 |  |
| CrPap-9 | *C. reinhardtii* | e_gwW.37.45.1 |  |
| CrPap-10 | *C. reinhardtii* | estExt_fgenesh2_kg.C_790010 |  |
| CrPap-11 | *C. reinhardtii* | estExt_fgenesh2_pg.C_170055 |  |
| VcPap-1 | *V. carteri* | estExt_Genewise1.C_420056 |  |
| VcPap-2 | *V. carteri* | estExt_fgenesh4_pg.C_50017 |  |
| VcPap-3 | *V. carteri* | fgenesh4_pg.C_scaffold_110000007 |  |
| VcPap-4 | *V. carteri* | estExt_fgenesh5_synt.C_80043 |  |
| VcPap-5 | *V. carteri* | estExt_Genewise1Plus.C_60031 |  |
| VcPap-6 | *V. carteri* | estExt_fgenesh4_pg.C_530079 |  |
| VcPap-7 | *V. carteri* | fgenesh4_pg.C_scaffold_65000015 |  |
| VcPap-8 | *V. carteri* | estExt_fgenesh4_pg.C_210128 |  |
| VcPap-9 | *V. carteri* | estExt_fgenesh4_pg.C_650017 |  |
| VcPap-10 | *V. carteri* | estExt_fgenesh4_pg.C_650020 |  |
| VcPap-11 | *V. carteri* | estExt_Genewise1.C_260103 |  |
| VcPap-12 | *V. carteri* | estExt_fgenesh4_pg.C_10088 |  |
| VcPap-13 | *V. carteri* | fgenesh4_pg.C_scaffold_65000022 |  |
| VcPap-14 | *V. carteri* | fgenesh4_pg.C_scaffold_70000005 |  |
| PpPap-1 | *P. patens* | estExt_Genewise1.C_490023 |  |
| PpPap-2 | *P. patens* | estExt_Genewise1.C_2850006 |  |
| PpPap-3 | *P. patens* | estExt_Genewise1.C_190224 |  |
| PpPap-4 | *P. patens* | estExt_Genewise1.C_2920028 |  |
| PpPap-5 | *P. patens* | fgenesh1_kg.scaffold_369000003 |  |
| PpPap-6 | *P. patens* | estExt_Genewise1.C_520042 |  |
| PpPap-7 | *P. patens* | estExt_gwp_gw1.C_3150037 |  |
| PpPap-8 | *P. patens* | e_gw1.199.86.1 |  |
| PpPap-9 | *P. patens* | estExt_Genewise1.C_790115 |  |
| PpPap-10 | *P. patens* | estExt_fgenesh1_pm.C_70048 |  |
| PpPap-11 | *P. patens* | estExt_Genewise1.C_580031 |  |
| SmPap-1 | *S. moellendorffii* | fgenesh2_pg.C_scaffold_104000045 |  |
| SmPap-2 | *S. moellendorffii* | fgenesh1_pm.C_scaffold_6000087 |  |
| SmPap-3 | *S. moellendorffii* | estExt_Genewise1.C_440151 |  |
| SmPap-4 | *S. moellendorffii* | e_gw1.20.119.1 |  |
| SmPap-5 | *S. moellendorffii* | e_gw1.33.84.1 |  |
| SmPap-6 | *S. moellendorffii* | estExt_Genewise1.C_51149 |  |
| SmPap-7 | *S. moellendorffii* | e_gw1.2.383.1 |  |
| SmPap-8 | *S. moellendorffii* | e_gw1.12.308.1 |  |
| SmPap-9 | *S. moellendorffii* | gw1.44.210.1 |  |
| SmPap-10 | *S. moellendorffii* | fgenesh1_kg.C_scaffold_33000014 |  |
| SmPap-11 | *S. moellendorffii* | gw1.17.925.1 |  |
| SmPap-12 | *S. moellendorffii* | e_gw1.29.325.1 |  |
| SmPap-13 | *S. moellendorffii* | estExt_Genewise1.C_01526 |  |
| SmPap-14 | *S. moellendorffii* | e_gw1.37.551.1 |  |
| SmPap-15 | *S. moellendorffii* | gw1.3.1656.1 |  |
| SmPap-16 | *S. moellendorffii* | e_gw1.0.638.1 |  |
| SmPap-17 | *S. moellendorffii* | e_gw1.45.552.1 |  |
| SmPap-18 | *S. moellendorffii* | e_gw1.40.124.1 |  |
| SmPap-19 | *S. moellendorffii* | estExt_fgenesh1_pm.C_1500002 |  |
| PtPap-1 | *P. trichocarpa* | eugene3.00021714 |  |
| PtPap-2 | *P. trichocarpa* | gw1.XIV.2019.1 |  |
| PtPap-3 | *P. trichocarpa* | grail3.0039026901 |  |
| PtPap-4 | *P. trichocarpa* | grail3.0028002001 |  |
| PtPap-5 | *P. trichocarpa* | fgenesh4_pm.C_LG_IV000141 |  |
| PtPap-6 | *P. trichocarpa* | fgenesh4_pm.C_LG_IV000139 |  |
| PtPap-7 | *P. trichocarpa* | eugene3.00081713 |  |
| PtPap-8 | *P. trichocarpa* | grail3.0005019101 |  |
| PtPap-9 | *P. trichocarpa* | eugene3.00040397 |  |
| PtPap-10 | *P. trichocarpa* | eugene3.00150664 |  |
| PtPap-11 | *P. trichocarpa* | fgenesh4_pg.C_LG_XI000811 |  |
| PtPap-12 | *P. trichocarpa* | eugene3.06990003 |  |
| PtPap-13 | *P. trichocarpa* | estExt_fgenesh4_pm.C_LG_IX0224 |  |
| PtPap-14 | *P. trichocarpa* | grail3.0045008602 |  |
| PtPap-15 | *P. trichocarpa* | eugene3.14770001 |  |
| PtPap-16 | *P. trichocarpa* | estExt_fgenesh4_pg.C_LG_II0263 |  |
| PtPap-17 | *P. trichocarpa* | fgenesh4_pm.C_LG_VII000314 |  |
| PtPap-18 | *P. trichocarpa* | fgenesh4_pm.C_scaffold_41000008 |  |
| PtPap-19 | *P. trichocarpa* | fgenesh4_pg.C_LG_V000555 |  |
| PtPap-20 | *P. trichocarpa* | grail3.0002061802 |  |
| PtPap-21 | *P. trichocarpa* | estExt_Genewise1_v1.C_LG_IV2709 |  |
| PtPap-22 | *P. trichocarpa* | fgenesh4_pm.C_scaffold_142000027 |  |
| PtPap-23 | *P. trichocarpa* | estExt_fgenesh4_pg.C_LG_XI0788 |  |
| PtPap-24 | *P. trichocarpa* | gw1.X.2843.1 |  |
| PtPap-25 | *P. trichocarpa* | estExt_Genewise1_v1.C_LG_IX2282 |  |
| PtPap-26 | *P. trichocarpa* | fgenesh4_pg.C_scaffold_14737000001 |  |
| PtPap-27 | *P. trichocarpa* | fgenesh4_pg.C_scaffold_3747000001 |  |
| PtPap-28 | *P. trichocarpa* | fgenesh4_pg.C_LG_V000289 |  |
| PtPap-29 | *P. trichocarpa* | fgenesh4_pg.C_scaffold_40000333 |  |
| PtPap-30 | *P. trichocarpa* | estExt_fgenesh4_pg.C_LG_XI0786 |  |
| PtPap-31 | *P. trichocarpa* | grail3.0017022102 |  |
| PtPap-32 | *P. trichocarpa* | fgenesh4_pg.C_LG_II000045 |  |
| PtPap-33 | *P. trichocarpa* | fgenesh4_pm.C_scaffold_66000095 |  |
| PtPap-34 | *P. trichocarpa* | eugene3.00051401 |  |
| PtPap-35 | *P. trichocarpa* | estExt_Genewise1_v1.C_LG_V2210 |  |
| PtPap-36 | *P. trichocarpa* | eugene3.00050275 |  |
| HvPap-1 | *H. vulgare* | BN000093 | SF42 |
| HvPap-2 | *H. vulgare* | AM941116 |  |
| HvPap-3 | *H. vulgare* | AM941117 |  |
| HvPap-4 | *H. vulgare* | AM941118 |  |
| HvPap-5 | *H. vulgare* | AM941119 |  |
| HvPap-6 | *H. vulgare* | AM941120 |  |
| HvPap-7 | *H. vulgare* | AM941121 |  |
| HvPap-8 | *H. vulgare* | AM941122 |  |
| HvPap-9 | *H. vulgare* | U94591 | EPA |
| HvPap-10 | *H. vulgare* | U19384 | EPB2 |
| HvPap-11 | *H. vulgare* | U19359 | EPB1 |
| HvPap-12 | *H. vulgare* | X05167 | Aleurain |
| HvPap-13 | *H. vulgare* | AM941123 |  |
| HvPap-14 | *H. vulgare* | AM941124 |  |
| HvPap-15 | *H. vulgare* | AM941125 |  |
| HvPap-16 | *H. vulgare* | AM941126 |  |
| HvPap-17 | *H. vulgare* | Z97022 |  |
| HvPap-18 | *H. vulgare* | AK251286 |  |
| HvPap-19 | *H. vulgare* | AJ310426 | HvCATH |
| HvPap-20 | *H. vulgare* | AM941127 |  |
| HvPap-21 | *H. vulgare* | AM941128 |  |
| HvPap-22 | *H. vulgare* | AM941129 |  |
| HvPap-23 | *H. vulgare* | AM941130 |  |
| HvPap-24 | *H. vulgare* | AM941131 |  |
| HvPap-25 | *H. vulgare* | AM941132 |  |
| HvPap-26 | *H. vulgare* | AM941133 |  |
| HvPap-27 | *H. vulgare* | AK251383 |  |
| HvPap-28 | *H. vulgare* | AM941134 |  |
| HvPap-29 | *H. vulgare* | AK248562 |  |
| HvPap-30 | *H. vulgare* | AK248416 |  |
| HvPap-31 | *H. vulgare* | AK249908 |  |
| HvPap-32 | *H. vulgare* | AK250687 |  |
| OsPap-1 | *O. sativa* | Os01g11840 |  |
| OsPap-2 | *O. sativa* | Os01g22670 |  |
| OsPap-3 | *O. sativa* | Os01g22680 |  |
| OsPap-4 | *O. sativa* | Os01g24550 |  |
| OsPap-5 | *O. sativa* | Os01g24560 |  |
| OsPap-6 | *O. sativa* | Os01g24570 |  |
| OsPap-7 | *O. sativa* | Os01g42780 |  |
| OsPap-8 | *O. sativa* | Os01g42790 |  |
| OsPap-9 | *O. sativa* | Os01g67980 | REP1 |
| OsPap-10 | *O. sativa* | Os01g11830 |  |
| OsPap-11 | *O. sativa* | Os01g73980 |  |
| OsPap-12 | *O. sativa* | Os03g54130 |  |
| OsPap-13 | *O. sativa* | Os04g01710 |  |
| OsPap-14 | *O. sativa* | Os04g12930 |  |
| OsPap-15 | *O. sativa* | Os04g13090 |  |
| OsPap-16 | *O. sativa* | Os04g13140 |  |
| OsPap-17 | *O. sativa* | Os04g24600 |  |
| OsPap-18 | *O. sativa* | Os04g57440 | Oryzain b |
| OsPap-19 | *O. sativa* | Os04g57490 | OsCP1 |
| OsPap-20 | *O. sativa* | Os05g01810 |  |
| OsPap-21 | *O. sativa* | Os06g38450 |  |
| OsPap-22 | *O. sativa* | Os07g29760 |  |
| OsPap-23 | *O. sativa* | Os07g01800 |  |
| OsPap-24 | *O. sativa* | Os04g55650 | Oryzain a |
| OsPap-25 | *O. sativa* | Os12g17540 |  |
| OsPap-26 | *O. sativa* | Os09g38920 |  |
| OsPap-27 | *O. sativa* | Os09g21370 |  |
| OsPap-28 | *O. sativa* | Os11g14900 | REPA |
| OsPap-29 | *O. sativa* | Os05g43230 |  |
| OsPap-30 | *O. sativa* | Os08g44270 |  |
| OsPap-31 | *O. sativa* | Os02g48450 |  |
| OsPap-32 | *O. sativa* | Os04g12660 |  |
| OsPap-33 | *O. sativa* | Os09g39160 |  |
| OsPap-34 | *O. sativa* | Os09g39090 |  |
| OsPap-35 | *O. sativa* | Os09g39070 |  |
| OsPap-36 | *O. sativa* | Os09g39110 |  |
| OsPap-37 | *O. sativa* | Os09g39120 |  |
| OsPap-38 | *O. sativa* | Os09g39100 |  |
| OsPap-39 | *O. sativa* | Os11g23770 |  |
| OsPap-40 | *O. sativa* | Os09g27030 | Oryzain c |
| OsPap-41 | *O. sativa* | Os02g27030 |  |
| OsPap-42 | *O. sativa* | Os09g39140 |  |
| OsPap-43 | *O. sativa* | Os09g39170 |  |
| OsPap-44 | *O. sativa* | Os09g39060 |  |
| OsPap-45 | *O. sativa* | Os05g24550 |  |
| AtPap-1 | *A. thaliana* | At1g47128 | RD21A |
| AtPap-2 | *A. thaliana* | At5g43060 |  |
| AtPap-3 | *A. thaliana* | At4g36880 |  |
| AtPap-4 | *A. thaliana* | At3g19390 |  |
| AtPap-5 | *A. thaliana* | At3g19400 |  |
| AtPap-6 | *A. thaliana* | At3g43960 |  |
| AtPap-7 | *A. thaliana* | At4g11310 |  |
| AtPap-8 | *A. thaliana* | At4g11320 |  |
| AtPap-9 | *A. thaliana* | At4g23520 |  |
| AtPap-10 | *A. thaliana* | At1g09850 | XBCP3 |
| AtPap-11 | *A. thaliana* | At3g48340 | CEP3 |
| AtPap-12 | *A. thaliana* | At3g48350 | CEP2 |
| AtPap-13 | *A. thaliana* | At5g50260 | CEP1 |
| AtPap-14 | *A. thaliana* | At1g20850 | XCP2 |
| AtPap-15 | *A. thaliana* | At4g35350 | XCP1 |
| AtPap-16 | *A. thaliana* | At5g45890 | SAG12 |
| AtPap-17 | *A. thaliana* | At1g06260 |  |
| AtPap-18 | *A. thaliana* | At2g34080 |  |
| AtPap-19 | *A. thaliana* | At1g29080 |  |
| AtPap-20 | *A. thaliana* | At1g29090 |  |
| AtPap-21 | *A. thaliana* | At2g27420 |  |
| AtPap-22 | *A. thaliana* | At3g49340 |  |
| AtPap-23 | *A. thaliana* | At4g39090 | RD19A |
| AtPap-24 | *A. thaliana* | At2g21430 |  |
| AtPap-25 | *A. thaliana* | At4g16190 |  |
| AtPap-26 | *A. thaliana* | At3g54940 |  |
| AtPap-27 | *A. thaliana* | At5g60360 | AtALEU |
| AtPap-28 | *A. thaliana* | At3g45310 |  |
| AtPap-29 | *A. thaliana* | At1g02305 |  |
| AtPap-30 | *A. thaliana* | At4g01610 |  |
| AtPap-31 | *A. thaliana* | At1g29110 |  |
| AtPap-32 | *A. thaliana* | At1g02300 |  |
